# Semitransparent Perovskite Solar Cells: Strategies, Prospects, and Challenges

**DOI:** 10.1007/s40820-026-02147-2

**Published:** 2026-04-07

**Authors:** Mohamad Firdaus Mohamad Noh, Nurul Affiqah Arzaee, Siti Naqiyah Sadikin, Muhammad Idzdihar Idris, Chien Fat Chau, Boon Kar Yap, Jingsong Huang, Ryousuke Ishikawa, Ahmad Wafi Mahmood Zuhdi

**Affiliations:** 1 Institute of Sustainable Energy (ISE), Universiti Tenaga Nasional (UNITEN), Jalan IKRAM-UNITEN, 43000 Kajang, Selangor Malaysia; 2 UNITEN R&D Sdn. Bhd., Universiti Tenaga Nasional (UNITEN), 43000 Kajang, Selangor Malaysia; 3 Faculty of Electronic and Computer Technology and Engineering, Universiti Teknikal Malaysia Melaka (UTeM), Jalan Hang Tuah Jaya, Durian Tunggal, 76100 Melaka, Malaysia; 4 Department of Electrical and Electronics Engineering, College of Engineering (COE), Universiti Tenaga Nasional (UNITEN), Jalan IKRAM-UNITEN, 43000 Kajang, Selangor Malaysia; 5 Oxford Suzhou Centre for Advanced Research, University of Oxford, Suzhou, 215123 People’s Republic of China; 6 Advanced Research Laboratories, Tokyo City University, Setagaya-ku, Tokyo , 158-8557 Japan

**Keywords:** Semitransparent perovskite solar cells, Light utilization efficiency, Average transmittance, Tandem, Building-integrated photovoltaics

## Abstract

Perovskite solar cells (PSCs) have achieved efficiencies comparable to established photovoltaic technologies, positioning them as strong contenders for next-generation solar energy systems. Amid these advances, research efforts are now directed toward extending PSCs functionality by combining efficient power generation with optical transparency, leading to the emergence of semitransparent PSCs (ST-PSCs). Advancing this technology requires deeper understanding of its design principles, applications, economic viability, and performance limitations. Therefore, this review examines key strategies for achieving an optimal balance between efficiency and optical transparency in ST-PSCs across small-, large-scale, and flexible devices, with emphasis on the functional roles and optimization approaches of individual components. Particular attention is devoted to stability challenges, including degradation mechanisms and recent strategies for improving their durability. The versatility of ST-PSCs is further illustrated through their integration into advanced architectures such as tandem and bifacial configurations, as well as broader applications including building-integrated photovoltaics, agrivoltaic systems, and photovoltaic–photoelectrochemical hybrids for hydrogen production. A technoeconomic perspective is also provided, with emphasis on manufacturing cost and levelized cost of electricity for ST-PSCs. Finally, the remaining challenges, particularly related to long-term stability, scalability, cost, and area constraints, are critically discussed, along with potential pathways toward commercial realization of ST-PSC technology.

## Highlights

- The development of semitransparent perovskite solar cells is analyzed from multiple optimization perspectives, focusing on the interdependence of efficiency, optical transparency, and stability.
- Application-oriented performance considerations are discussed to relate laboratory-scale demonstrations to realistic deployment scenarios.
- Critical bottlenecks limiting the development and deployment of semitransparent devices are identified, and practical optimization directions for next-generation applications are outlined.

## Graphical abstract

**Figure:**
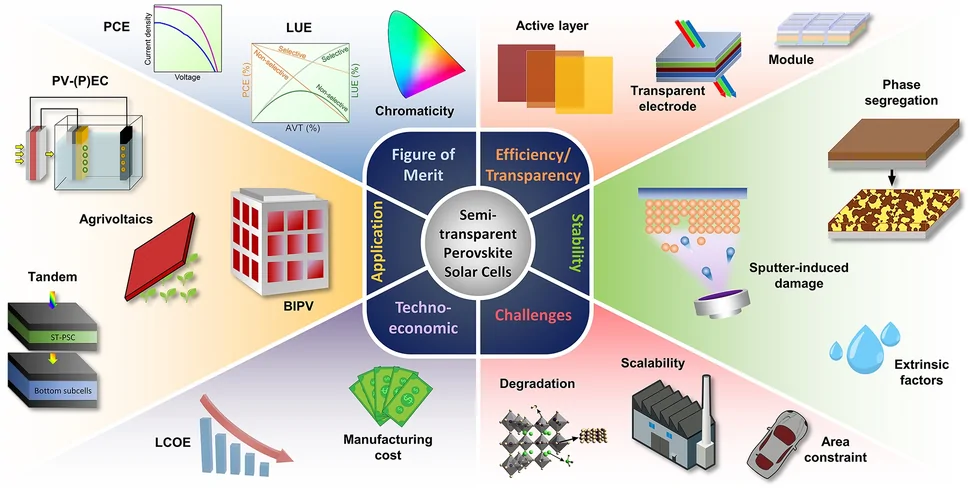

## Introduction

The transition to clean energy remains a central focus of the United Nations Sustainable Development Goals (SDGs). Among the available renewable energy options, solar power plays a crucial role in reducing CO_2_ emissions and mitigating climate change. Over the past decade, organic–inorganic metal halide perovskite solar cells (PSCs) have emerged as one of the most promising photovoltaic (PV) technologies due to their exceptional performance combined with low-cost and facile solution-processable fabrication [1, 2]. Within just a few years of development, the power conversion efficiency (PCE) of PSCs has soared to 27% [3] owing to their outstanding optoelectronic properties such as low exciton binding energy, long carrier diffusion length, strong light absorption, and high defect tolerance [4–7]. Recently, the development of PSCs in a semitransparent configuration, known as semitransparent PSCs (ST-PSCs), has opened new possibilities for sustainable applications such as building-integrated photovoltaics (BIPV), portable electronics, and tandem solar cells.

At present, semitransparency in conventional PV systems is typically realized through the spatial segmentation of opaque crystalline silicon solar cells, where gaps or micro-holes are introduced between adjacent cells to allow light transmission. However, while this approach enhances transparency, it substantially reduces the overall PCE of the system [8]. III–V semiconductor solar cells such as GaAs and GaInP can achieve both high PCE and partial transparency without spatial segmentation, yet their high fabrication cost originating from epitaxial growth and expensive substrates limits large-scale adoption [9]. Thin-film technologies such as Cu(In,Ga)Se_2_ (CIGS) and cadmium telluride (CdTe) can also be made flexible and semitransparent, but the scarcity of constituent elements and costly processing remain obstacles to commercialization [10]. Emerging alternatives including organic solar cells, dye-sensitized solar cells, and antimony chalcogenide-based devices offer low-cost fabrication and inherently thin active layers, which are suitable for semitransparent applications. However, their efficiencies still lag behind those of perovskite-based devices [11–13].

**Fig. 1:**
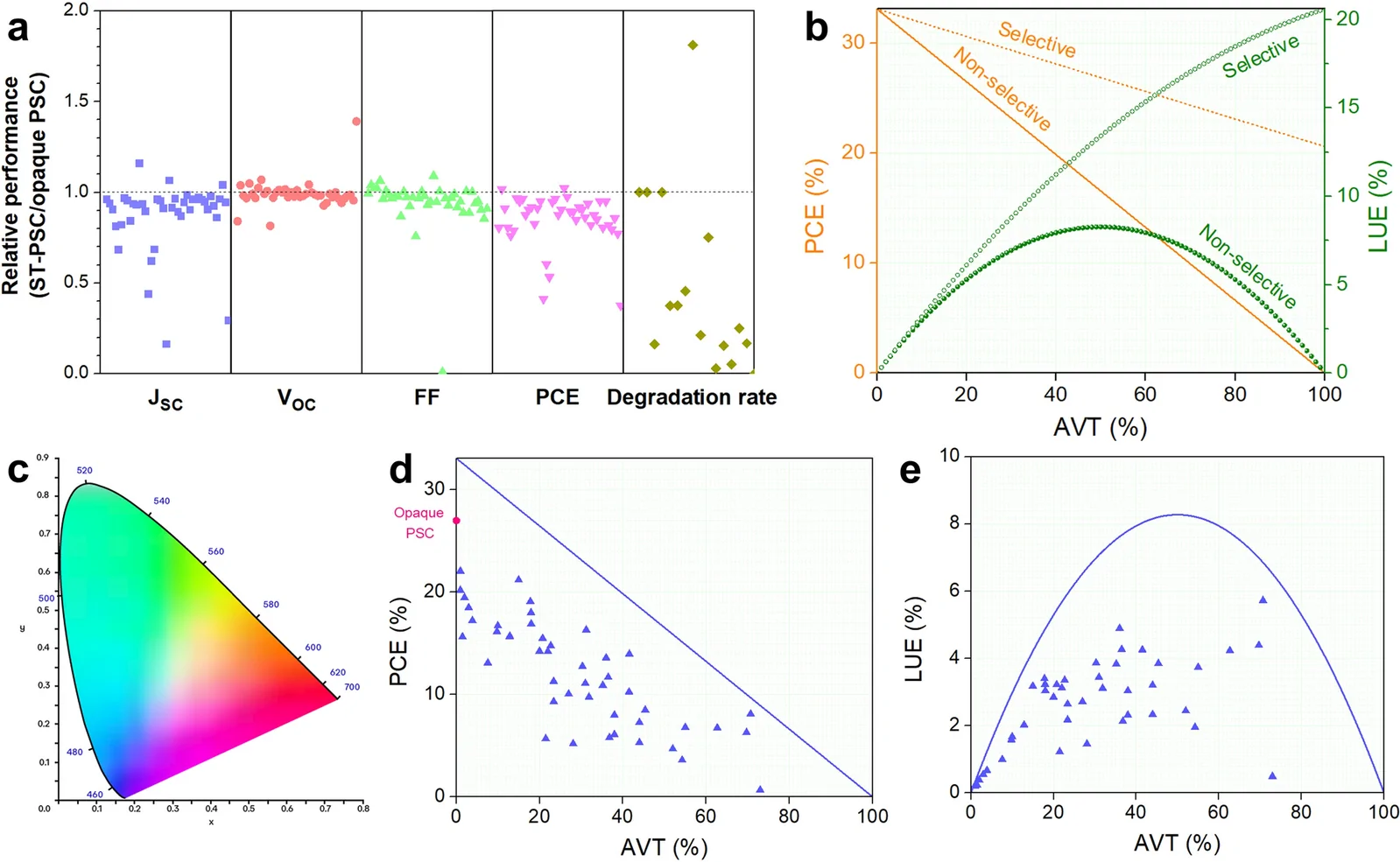
**a** Relative photovoltaic performance parameters and degradation rates of ST-PSCs compared with opaque PSCs, based on a comprehensive analysis of literature reports. **b** Shockley–Queisser limit of PCE and LUE as a function of AVT for non-wavelength-selective and UV/NIR wavelength-selective semitransparent PV technologies. **c** CIE 1931 color space chromaticity diagram. **d** PCE and **e** LUE as functions of AVT for representative ST-PSCs reported in recent literature, together with their corresponding theoretical limits

ST-PSCs, in contrast, combine several superior attributes, including high PCE, tunable bandgap and transparency, simple fabrication, low processing cost, compatibility with tandem architectures, aesthetic versatility, and suitability for flexible designs. Given these distinct advantages, major PV manufacturers such as LONGi and Trina Solar have begun exploring the potential of ST-PSCs, particularly for the purpose of enhancing the performance of their products through tandem integration [14, 15]. Currently, perovskite-based tandem devices have reached certified PCEs as high as 34.9% [16], while standalone ST-PSCs has achieved up to 23.3% in bifacial configurations [17]. Based on comparisons across numerous literature reports, ST-PSCs generally exhibit lower PCE than opaque devices. However, they often show improved operational stability, as reflected by their lower degradation rates (Fig. 1a). Motivated by these advancements and growing industrial interest, this review provides a comprehensive overview of the recent developments and future prospect of ST-PSCs.

The discussion begins with an introduction to several key figures of merit that are essential for evaluating ST-PSC performance from both electrical and optical perspectives. The core section highlights the key strategies employed in optimizing the trade-off between efficiency and transparency by systematically examining each critical component of the device architecture, including the perovskite active layer, electron, and hole transport layers (ETL and HTL), transparent electrodes, and other layers. Recent advances in the fabrication of flexible ST-PSCs and scaling up of semitransparent perovskite minimodules are also elaborated. A dedicated discussion focusing on the critical issues related to the stability of ST-PSCs and their mitigation strategies is also presented. Subsequently, the diverse potential applications of ST-PSCs in areas such as tandem solar cells, bifacial PV, BIPV, and agrivoltaic systems are explored. A technoeconomic overview is further provided, focusing on manufacturing cost and levelized cost of electricity (LCOE) as key metrics for assessing the economic viability of ST-PSC technologies. Finally, the remaining challenges and future research directions necessary to achieve highly efficient, durable, and commercially viable ST-PSC technology are highlighted. The insights summarized in this review are expected to serve as a valuable reference for guiding continued innovation and large-scale deployment of next-generation transparent PV technologies.

## Figure of Merit for ST-PSC

ST-PSCs allow partial transmission of visible light while harvesting part or all of the photons with energies equal to or greater than the bandgap of the light-absorbing layer. Since both absorption and transmission occur within the same spectral region, a fundamental trade-off exists between the PCE and average transmittance (AVT). In addition to these two parameters, the overall performance of ST-PSCs is also evaluated using metrics such as light utilization efficiency (LUE), CIELab chromaticity coordinates, and color rendering index (CRI).

PCE is the ultimate figure of merit for any solar cells because it directly represents the effectiveness of the device in converting sunlight into electrical power. Similar to other solar cell technologies, the PCE (*η*) of ST-PSCs is calculated using the widely known equation as follows [18]:

1 $$\eta =\frac{{J}_{\mathrm{SC}}\times {V}_{\mathrm{OC}}\times \mathrm{FF}}{{P}_{\mathrm{in}}}\times 100\%$$

where *J*_SC_, *V*_OC_, and FF represent the short-circuit current density (mA cm^−2^), open-circuit voltage (V), and fill factor, respectively, and *P*_in_ denotes the incident light power density (mW cm^−2^). The PCE usually reflects the behavior of the device under standard illumination conditions (AM 1.5).

Another crucial factor determining the performance of ST-PSCs is their optical transparency. A common approach to quantify transparency is through the calculation of the AVT across the visible spectrum. The AVT parameter accounts for the human eye’s varying sensitivity to different wavelengths and is expressed as follows [19]:

2 $$\mathrm{AVT}=\frac{\int T(\lambda )V(\lambda )S(\lambda )d\lambda }{\int V(\lambda )S(\lambda )d\lambda }$$

where *λ* represents the wavelength, *T(λ)* is the transmittance of the device, *V(λ)* denotes the photopic response of the human eye (dataset provided by the International Commission on Illumination) [20], and *S(λ)* corresponds to the solar photon flux under AM 1.5 illumination. The boundaries of the visible spectrum are not strictly defined, as they depend on both the intensity of light perceived by the human eye and the sensitivity of the observer. Although the human eye can detect wavelengths from approximately 310 nm in the ultraviolet (UV) until 1100 nm in the near infrared (NIR), the visible range is generally considered to span from about 360–400 nm at the lower end to about 760–830 nm at the upper end [21]. For this reason, the wavelength range used in AVT evaluation varies among literatures. However, a standard range of 380–780 nm can be adopted, as defined in ISO 9050:2003 [22, 23].

Since the PCE and AVT are typically inversely correlated, a combined performance indicator is needed to fairly assess the overall advancement of ST-PSC. To this end, Traverse et al. introduced the LUE metric, which is defined as [24]:

3 $$\mathrm{LUE}=\mathrm{PCE}\times \mathrm{AVT}$$

This metric provides a practical means of comparing ST-PSC performance relative to the theoretical limits. Enhancing transparency (higher AVT) generally leads to a reduction in PCE and vice versa, which directly influences the overall LUE value. The optimal balance between transparency and efficiency for single-junction ST-PSC with optimum bandgap occurs at an AVT of 50%, corresponding to a maximum LUE of around 8.2% (Fig. 1b). This is because perovskite is a non-wavelength-selective absorber, where it absorbs a broad spectrum of photons with energies above their bandgap. Theoretically, solar cells that can selectively absorb UV and NIR photons and completely transmit the visible light can reach a maximum LUE of 20.6% [25].

Another important aspect in the development of ST-PSCs is color perception, which influences their aesthetic appeal. The color of a ST-PSC can be quantitatively described using several models, among which the International Commission on Illumination (CIE) 1931 chromaticity diagram is the most widely adopted (Fig. 1c). In this system, the perceived color is expressed as chromaticity coordinates (*x*, *y*) derived from a measured spectrum [12]. A neutral color appears near the coordinates (0.3333, 0.3333), while the standard daylight AM 1.5G illumination corresponds to (0.3202, 0.3324) [26]. Alternatively, color can be assessed using the CIELAB color space, where color is represented by three parameters: lightness (L*) and two chromaticity coordinates (a* and b*), which correspond to the red–green and yellow–blue axes, respectively [27, 28]. Another important figure of merit used to evaluate the visual appearance of ST-PSCs is the CRI value. The CRI measures how accurately the light transmitted through a semitransparent solar cell reproduces the true colors of illuminated objects compared to those observed under a standard white light source [28]. A CRI value approaching 100 indicates that the transmitted light closely matches the reference illumination, implying minimal color distortion.

## Key Strategies for Efficiency and Transparency Optimization

Recent achievements in the PCE and LUE of ST-PSCs are presented in Fig. 1d, e, highlighting steady progress toward the theoretical maximum. Interestingly, relatively high LUE values have been reported at AVT levels above 50%, despite the limited number of studies. As shown in Fig. 1a, the *V*_OC_ of ST-PSCs is comparable to that of opaque counterparts, suggesting that electrical recombination is not the primary factor behind performance losses. In contrast, a noticeable difference is observed in *J*_SC_, indicating that optical losses arising from high transmittance, parasitic absorption, and undesired reflection are the main contributors. Reduced FF, caused by series resistance of the transparent electrode, also plays a significant role. Key strategies reported in the literature to simultaneously improve efficiency and transparency, along with their corresponding figures of merit, are summarized in Table 1. Understanding these strategies, as discussed in this section, is essential for approaching the theoretical limit of LUE.

**Table 1:** Summary of key strategies employed in ST-PSC development and their corresponding figures of merit

Key strategies	Approach	Buffer layer/Electrode	Active area (cm^2^)	ST-PSC PCE (%)	Opaque PSC PCE (%)	AVT (%)	LUE (%)	CRI	Refs
Thickness control	Spin speed control	Au	0.17	10.1	12.5	27.0	2.72	–	[29]
Thickness control	Precursor concentration control	Ag	1.00	5.7	–	21.5	1.23	–	[30]
Thickness control	Precursor concentration control	NiO/ITO	–	19.5	19.2	2.0	0.39	–	[31]
Thickness control	Precursor concentration control	NiO/ITO	–	12.8	-	30.3	3.87	–	[31]
Thickness control	Evaporation-solution method	ITO/Cu	0.13	18.5	20.3	–	–	–	[32]
Thickness control	Co-evaporation	Cu/Ag	0.05	9.3	11.6	23.4	2.18	–	[33]
Thickness control	Co-evaporation + material control	Al/Ag	0.05	3.6	4.7	54.3	1.95	–	[19]
Thickness control	Co-evaporation + material control	Al/Ag	0.05	9.8	12.4	31.9	3.12	–	[19]
Bandgap tuning (improve AVT)	Halide substitution	ITO	0.42	6.3	–	69.7	4.39	–	[34]
Bandgap tuning (improve AVT)	Halide substitution	ITO	48.00	5.5	–	59.4	3.24	–	[34]
Bandgap tuning (improve AVT)	Halide substitution	MoO_3_/ITO	0.06	0.7	0.7	73.0	0.49	95.0	[35]
Bandgap tuning (improve V_OC_)	Passivation (surface)	MoO_3_/ITO	0.10	18.6	19.6	–	–	–	[36]
Bandgap tuning (improve V_OC_)	Passivation (surface)	ITO	1.00	8.1	–	70.7	5.73	60.4	[37]
Bandgap tuning (morphology)	Solvent modification	SnO_2_/IZO	0.08	17.6	18.3	–	–	–	[15]
Bandgap tuning (phase segregation)	A-site cation engineering	MoO_3_/Au/MoO_3_	0.16	15.5	17.8	20.8	3.22	–	[38]
Bandgap tuning (phase segregation)	Additive in perovskite	Ag/MoO_3_	0.10	14.2	–	22.0	3.13	–	[39]
Bandgap tuning (phase segregation)	Passivation (buried interface)	SnO_2_/IZO/Ag	0.12	18.5	20.5	3.0	0.56	–	[40]
Bandgap tuning (phase segregation)	Passivation (grain boundary)	SnO_2_/IZO	0.06	17.9	19.5	–	–	–	[41]
Microstructure engineering	Porous film	PEDOT:PSS/Ni	0.06	5.2	-	28.1	1.46	–	[42]
Microstructure engineering	Porous film + blocking layer	Ni	0.09	6.1	7.4	38.0	2.32	–	[43]
Microstructure engineering	Porous film	Au	0.08	11.7	–	36.5	4.27	–	[44]
Microstructure engineering	Porous film + nanopillar	MoO_3_/ITO	0.06	8.5	9.3	45.4	3.85	–	[45]
Microstructure engineering	Porous film + nanopillar	MoO_3_/ITO	0.06	10.3	–	41.5	4.26	–	[45]
Microstructure engineering	Micropatterning	Au	4.00	13.1	13.8	7.6	1.00	–	[46]
Microstructure engineering	Micropatterning	Au	4.00	5.8	14.1	36.8	2.13	–	[46]
Microstructure engineering	Micropatterning	Au	0.08	11.1	18.4	31.0	3.44	95.5	[47]
Microstructure engineering	Micropatterning	Au	0.08	8.0	–	38.0	3.04	–	[47]
ETL (mesoporous)	Perovskite thickness control	ITO	–	16.9	–	18.0	3.04	–	[48]
ETL (oxygen vacancies)	Surface passivation	ITO	0.30	6.8	–	55.0	3.74	–	[49]
ETL (oxygen vacancies)	Surface passivation	ITO	2.90	4.7	–	52.0	2.44	45.0	[49]
HTL (film quality)	Solvent modification	SnO_2_/ITO	0.16	19.1	–	17.8	3.40	–	[50]
HTL (film quality)	Solvent modification	SnO_2_/ITO	0.16	10.9	20.5	35.2	3.84	–	[50]
HTL (ion diffusion)	Prolong oxidation of spiro-OMeTAD	MoO_3_/IZO	0.07	22.0	23.3	–	–	–	[51]
HTL (UV stability)	Cross-linkable small molecules	MoO_3_/IZO/Au	–	18.8	19.5	–	–	–	[52]
HTL (thermal stability)	Cross-linkable small molecules	MoO_3_/Au/MoO_3_	0.16	16.7	19.1	10.0	1.67	–	[53]
TCO (conductivity)	Metal grid deployment	SnO_2_/ITO/Ag	21.70	20.2	22.3	1.0	0.20	–	[54]
TCO (conductivity)	Metal grid deployment	SnO_2_/ITO/Ag	0.08	22.1	–	1.0	0.22	–	[54]
TCO (indium scarcity)	ITO replacement with AZO	SnO_2_/AZO/Cu	0.05	18.9	18.5	–	–	–	[55]
TCO (carrier mobility)	ITO replacement with ICO	MoO_3_/ICO/Ag	0.14	19.0	–	–	–	–	[56]
TCO (carrier mobility)	ITO replacement with ICO	ICO/Ag	0.14	17.6	–	–	–	–	[57]
TCO (ion bombardment)	Increasing SnO_2_ thickness	SnO_2_/ITO	0.16	18.3	18.8	–	–	–	[58]
TCO (ion bombardment)	V_2_O_5_ buffer layer	V_2_O_5_/ITO	–	15.7	17.5	1.5	0.23	-	[59]
TCO (ion bombardment)	Facing target sputtering	ZnO/IZTO	1.08	15.7	17.6	12.9	2.03	-	[60]
TCO (ion bombardment)	Isolated plasma soft deposition	IGTO/LiF	0.19	18.7	22.1	–	–	–	[61]
TCO (ion bombardment)	Isolated plasma soft deposition	ZnO/ITO	0.30	15.5	–	–	–	–	[62]
TCO (humidity/oxygen)	Metal electrode replacement	ZnO/ITO	–	16.3	17.8	–	–	–	[63]
Metal-based (humidity/oxygen)	Compact SnO_2_ by ALD	SnO_2_/AgNW	–	17.5	19.4	–	–	–	[64]
Metal-based (conductivity)	Metal seed layer	Cr/Au	1.00	19.8	23.0	–	–	–	[65]
Metal-based (conductivity)	DMD with metal seed layer	MoO_3_/Au/Ag/MoO_3_	0.52	18.0	19.2	18.0	3.23	–	[66]
Metal-based (conductivity)	AgNW + binder	PVP + AgNW	–	17.9	21.8	–	–	–	[67]
Metal-based (conductivity)	Chemical welding	rGO + AgNW + PDMS	–	16.2	18.5	9.8	1.58	–	[68]
Metal-based (AVT)	Asymmetric DMD	MoO_3_/Au/MoO_3_	–	11.3	13.3	23.4	2.64	11.1	[69]
Metal-based (tilted angle)	DMD thickness control	WO_3_/Ag/WO_3_	0.12	21.2	–	15.0	3.18	80	[70]
Metal-based (Cu diffusion)	Inserting ITO under Cu	SnO_2_/ITO/Cu	0.16	17.8	18.8	–	–	–	[71]
Metal-based (Au diffusion)	PANI doping in HTL	Au	0.18	15.7	19.5	12.9	2.01	–	[72]
Carbon-based	MWCNT	CNT/ITO	0.09	22.2	23.4	–	–	–	[73]
Carbon-based	SWCNT	CNT/ITO	0.09	21.4	23.4	–	–	–	[73]
Carbon-based	spiro-OMeTAD embedded SWCNT	CNT	0.09	17.2	–	–	–	–	[74]
Carbon-based	SWCNT via FCCVD	CNT	0.09	19.1	23.4	–	–	–	[75]
Carbon-based	SWCNT via FCCVD	CNT	0.09	17.1	21.6	–	–	–	[75]
Carbon-based	SWCNT via FCCVD	CNT	0.08	13.8	16.1	–	–	–	[76]
Antireflective coating	ARC on both sides	SnO_2_/IZO	0.11	19.3	–	–	–	–	[77]
Antireflective coating	ARC on both sides	ITO	0.05	20.1	–	–	–	–	[78]
Photon conversion	Up-conversion layer	Au/MoO_3_	–	4.1	–	16.0	0.66	–	[79]
Photon conversion	Down-conversion layer	SnO_2_/ICO	0.09	13.4	–	–	–	–	[80]
Photon conversion	Down-conversion layer	MoO_3_/Au/MoO_3_	0.12	14.3	18.5	20.0	2.85	–	[81]
Doping	Cation doping	NiO/ITO	0.15	7.3	–	44.0	3.20	–	[82]
Doping	Cation doping	NiO/ITO	10.00	5.3	–	44.0	2.34	–	[82]
Doping	Polymer-perovskite blend	Au	0.04	13.6	–	36.0	4.90	–	[83]
Additional layer	Additional functional layer	ITO/Ag	0.09	14.8	–	22.7	3.36	–	[84]
Encapsulation	Encapsulation using PIB	ITO	1.00	6.8	18.0	62.7	4.23	–	[85]
Encapsulation	Lamination via TCA	SnO_2_/ITO	–	20.6	–	–	–	–	[86]
Encapsulation	Thermocompresssion	ITO	0.09	17.2	–	3.9	0.67	–	[87]
Encapsulation	Halide diffusion lamination	ITO	–	18.9	–	–	–	–	[88]

### Active Layer

Strategies to achieve a balance between efficiency and transparency by adjusting the perovskite layers can be categorized into four main approaches: controlling the film thickness, tuning the band gap, engineering the microstructure, and employing other complementary methods. Among these, the first and second strategies are the most widely used. Each approach offers a distinct trade-off between transparency and PV performance, and their precise optimization is essential for the realization of high-efficiency ST-PSCs.

#### Thickness Control

**Fig. 2:**
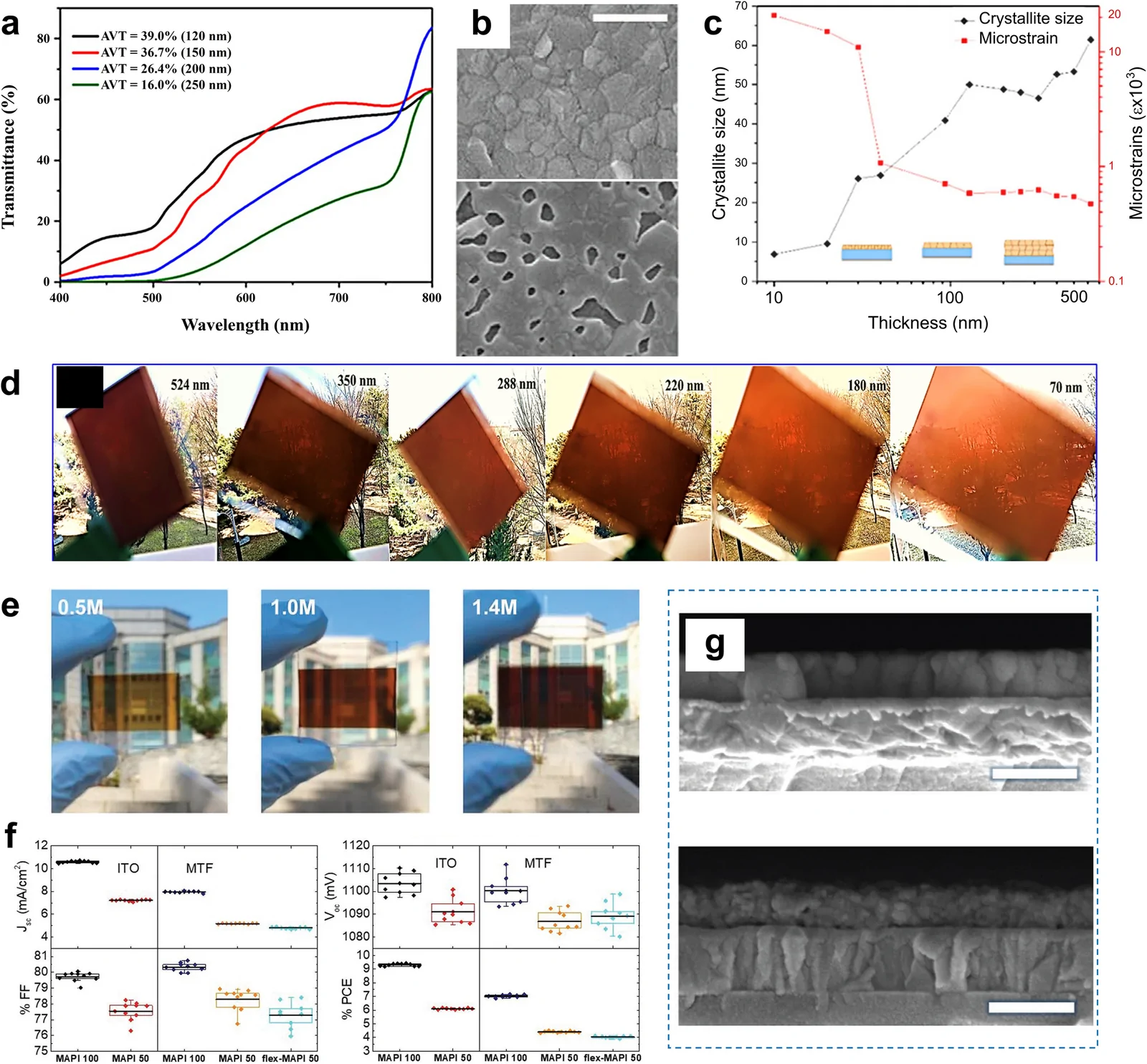
**a** Transmittance spectra of the perovskite films with different thickness. Reproduced under terms of the CC-BY license [81]. Copyright 2024, The Authors, published by Wiley. **b** SEM images of perovskite films with thickness of (top) 450 nm and (bottom) 100 nm deposited on PTAA. The SEM scale bar is 500 nm. Reproduced with permission [90]. Copyright 2020, Wiley. **c** Microstrain and crystallite size of perovskite film with varying thickness. Reproduced under terms of the CC-BY license [30]. Copyright 2024, The Authors, published by American Chemical Society. **d** Photographs of ST-PSCs prepared using different concentrations of perovskite solution. Reproduced with permission [31]. Copyright 2022, Wiley. **e** Photographs of ST-PSCs with different perovskite thickness prepared using different spin speed. Reproduced with permission [29]. Copyright 2018, Elsevier. **f** PV parameters of ten ST-PSCs fabricated using co-evaporation with various thicknesses and substrate types. Reproduced under terms of the CC-BY-NC-ND license [33]. Copyright 2022, The Authors, published by Wiley. **g** Cross-sectional SEM images of thin perovskite films prepared by co-evaporation method, showing (top) columnar growth of PEACsPbI_3_ and (bottom) disordered growth of FACsPbIBr. The SEM scale bar is 200 nm. Reproduced under terms of the CC-BY license [19]. Copyright 2023, The Authors, published by Wiley

Reducing the perovskite layer thickness is the most common strategy to impart semitransparency. In conventional opaque PSCs, the perovskite layer is usually about 400–800 nm thick to ensure strong light absorption, as perovskites already have a high absorption coefficient [89]. For ST-PSCs, the thickness is generally reduced to below 300 nm [81], and, in some cases, ultrathin layers as low as 10 nm have been demonstrated [30]. As shown in Fig. 2a, thinner perovskite films exhibit higher transmittance due to limited volume of photoactive layer to absorb the light. The improved transparency comes at the expense of a reduced number of photogenerated charges, leading to a lower J_SC_.

Film thickness not only affects optical absorption but also strongly influences film morphology, where the thin perovskite layer typically shows poor surface coverage. As reported by Jung et al., a 450-nm-thick perovskite deposited on a PTAA HTL by one-step spin coating with anti-solvent treatment yielded compact, pinholes-free films, whereas a 100-nm-thick layer exhibited numerous randomly distributed pinholes (Fig. 2b) [90]. This problem originates from poor wettability, since the surface energy mismatch between the hydrophilic perovskite precursor solution and the hydrophobic surface of the charge transport layer reduces nucleation density and hinders rapid mass transfer during crystallization [91]. As a consequence, the ultrathin films fail to achieve complete coverage and cause direct contact between the HTL and ETL, which amplifies the charge recombination rate [92].

Excessive thinning also introduces lattice strain that degrades the ST-PSCs performance. Strain in perovskite originates from the disrupted crystal growth particularly during annealing as a result of both thermal expansion coefficient mismatch and lattice mismatch between the perovskite and underlying charge transport layer [93, 94]. Such strain can induce defect formation and ion migration, which creates recombination centers that impair charge transport and deteriorates long-term stability [95]. As shown in Fig. 2c, microstrain increases markedly for thickness below 30–40 nm, which indicates the appearance of lattice distortion [30, 33].

Thus, a central challenge is to fabricate ultrathin perovskite layers that combine high optical transparency with uniform coverage and minimal defects. The minimum practical thickness depends on deposition method, fabrication environment, and material system. In wet processing, spin coating can offer straightforward thickness control by tuning the spin speed or precursor concentration. Przypis et al. varied MAPbI_3_ thickness between 3 and 625 nm and identified ~ 30 nm as the threshold for maintaining reasonable performance. Using this thickness, they fabricated flexible ST-PSCs (1 cm^2^) with 5.7% PCE and 21.5% AVT [30]. Similarly, Wang et al. obtain higher transparency by decreasing the thickness of MAPbI_3-*x*_Cl_*x*_-based perovskite from 524 to 70 nm through an increased spin speed (Fig. 2d). However, excessive speeds induced pinholes and island-like growth, with the optimal ~ 350 nm thickness balancing 27% AVT and 10.0% PCE [29]. Reducing the precursor concentration from 1.4 to 0.5 M also reduced the perovskite thickness and raised the AVT from 2% to 30% (Fig. 2e), as shown by Jeong et al. [31]. While thicker films achieved higher PCEs (18.4%) due to increased absorption, thinner films yielded superior LUE owing to the balance between relatively high PCE (12.8%) and high transparency.

For vapor-based deposition, co-evaporation technique can provide precise thickness and stoichiometry control by monitoring the flux of inorganic (e.g., PbI_2_, CsI) and organic (e.g., MAI, FAI) precursors during deposition [32]. Besides, evaporation process encourages slow crystal growth, which leads to the formation of numerous crystal nuclei and small grain size, enabling the fabrication of much thinner films. Paliwal et al. fabricated 50-nm MAPbI_3_ layers with uniform, pinholes-free morphology across different substrates and obtained ST-PSCs with highly reproducible *J*_SC_ and PCEs due to the consistent thickness (Fig. 2f) [33]. However, similar to solution methods, evaporated perovskites also require a minimum thickness for continuous coverage, which depends on the growth behavior of particular material. For instance, PEACsPbI_3_ layer forms compact ultrathin films at 10 nm due to preferential (001) columnar growth, whereas FACsPbIBr requires at least 50 nm thickness to obtain pinhole-free structure owing to disordered growth orientation (Fig. 2g) [19]. Consequently, ST-PSCs based on the ultrathin PEACsPbI_3_ reached an impressive AVT of 54% with PCE of 3.6%.

#### Bandgap Tuning

**Fig. 3:**
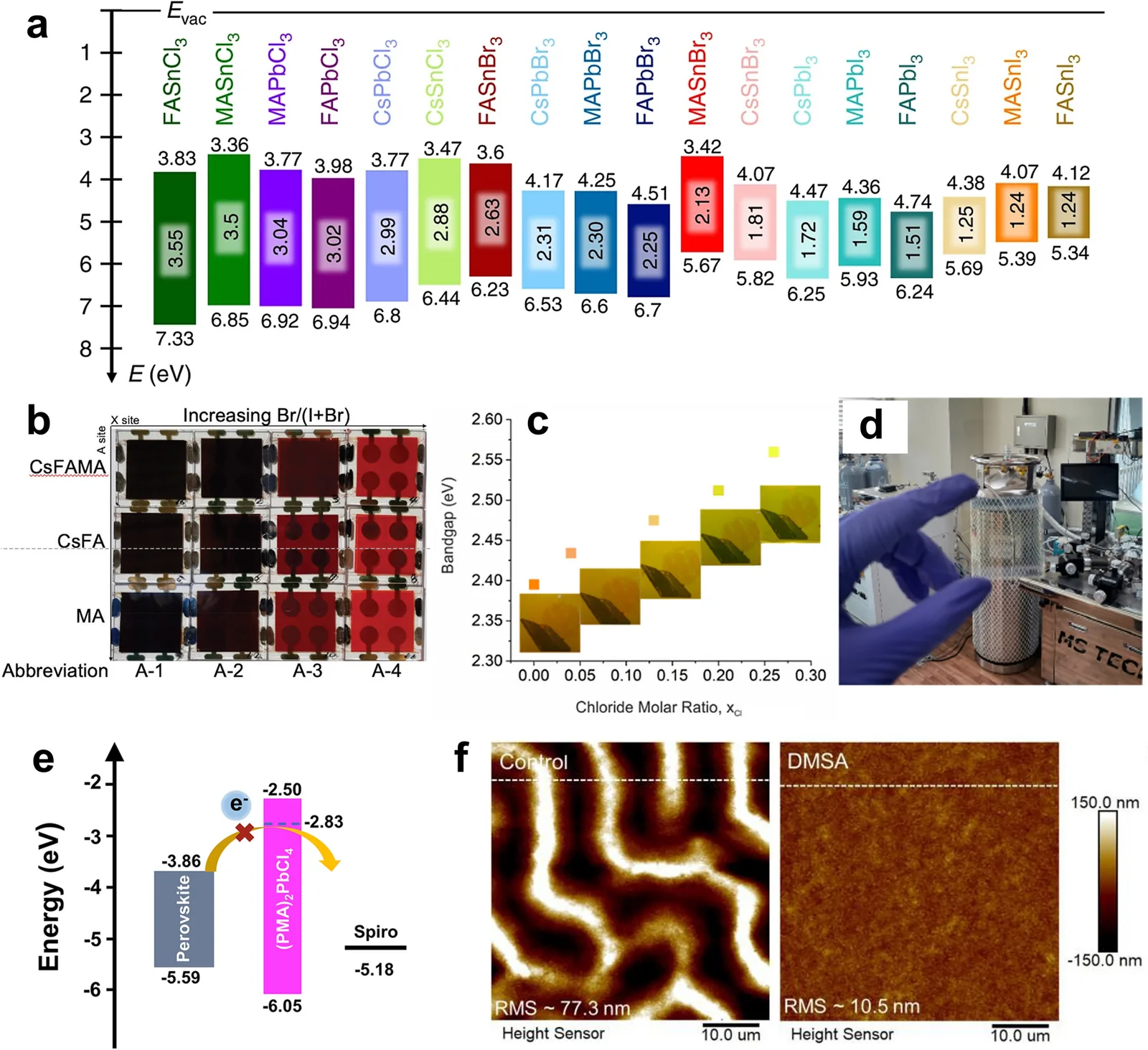
**a** Energy-level diagram of various metal halide perovskites. Reproduced under terms of the CC-BY license [96]. Copyright 2019, The Authors, published by Springer Nature. **b** Photographs of perovskite devices with different cations (MA, FA, Cs) and varying I-Br content. Reproduced under terms of the CC-BY license [38]. Copyright 2022, The Authors, published by Wiley. **c** Bandgap variation for MAPb(Br_1-x_Cl_x_)_3_ with different chloride molar ratio. Elsevier, 2022. Reproduced with permission [34]. Copyright 2022, Elsevier. **d** Photograph of the MAPbCl_3_-based ST-PSC. Reproduced with permission [35]. Copyright 2024, American Chemical Society. **e** Schematic illustration of the energy-level alignment between the perovskite and the wide-bandgap 2D (PMA)_2_PbCl_4_ layer. Reproduced under terms of the CC-BY license [36]. Copyright 2023, The Authors, published by Springer Nature. **f** AFM images of wide-band-gap perovskite films prepared via DMF-based (control) and DMSO-based (DMSA) solvent system. Reproduced with permission [15]. Copyright 2024, Wiley

Tuning the perovskite band gap offers an effective way to control which portions of the light spectrum are absorbed or transmitted. The band gap can be broadly tuned from 1.24 to 3.55 eV by tailoring the perovskite composition, specifically through substitution or mixing of the A-site cations (e.g., MA, FA, Cs, Rb), B-site metals (e.g., Pb, Sn), and X-site halides (e.g., I, Br, Cl), as shown in Fig. 3a [7, 96]. For wet-based techniques, the composition can be controlled by adjusting the precursor molar ratios during solution preparation, whereas for vapor-based techniques, it can be tuned by regulating the precursor sublimation rates during thermal evaporation. While compositional tuning is essential to achieve the desired band gap and optical transparency, it also causes a shifting in the energy levels of the valence band maximum (VBM) and conduction band minimum (CBM). The VBM is mainly influenced by the interaction between the metal *s* orbitals and the halide *p* orbitals. Therefore, it moves to higher energy when the halide changes from I to Br to Cl, and also when Sn is replaced with Pb. The CBM, on the other hand, depends on the interaction between the metal *p* orbitals and the halide *s* orbitals. As a result, it shifts to lower energy across the same halide series (I → Br → Cl) and when Pb is substituted with Sn [96].

A-site cations are primarily modified to improve the structural stability of the perovskite crystal, but they have minimal influence on the band gap and optical transparency. B-site metal cations are often alloyed to produce narrow-band-gap perovskites, which are less relevant for achieving semitransparency [97–100]. Semitransparency is mainly tuned by adjusting the halide composition at the X-site. Typically, I-based perovskites exhibit the narrowest band gaps, followed by Br-based and then Cl-based compositions. The value exceeding 1.65 eV is generally regarded as wide band gap for perovskite materials [101–103]. As illustrated in Fig. 3b, perovskite films rich in iodide appear dark brown and gradually lighten to a more transparent brown as iodide is replaced with bromide [104]. MAPbBr_3_ perovskites show an orange-yellow hue, and the color slowly shifts toward light yellow with increasing chloride content (Fig. 3c) [34]. Meanwhile, perovskite films based on fully chlorinated compositions, i.e., MAPbCl_3_, appear nearly transparent (Fig. 3d) [35].

Matteocci et al. investigated the effect of partial chloride substitution in MAPbBr_3_ perovskites on the optical band gap and device performance. For 500-nm-thick MAPb(Br_1-x_Cl_x_)_3_ films, the band gap increased linearly from 2.39 eV at *x*_Cl_ = 0 to 2.56 eV at *x*_Cl_ = 0.26. The optimal composition was achieved at *x*_Cl_ = 0.13, where high crystal quality and minimal trap-assisted recombination at the interface were obtained, resulting in a PCE of 6.3%, an AVT of 69.4%, and a LUE of 4.37% [34]. “Glass-clear,” neutral-colored ST-PSCs with a high CRI can be achieved by confining the absorption range to the UV region. This can be accomplished by widening the band gap above 2.85 eV, which typically requires a chloride-to-bromide ratio of at least 80:20 [35, 105]. Although this limits the PCE to very low values, the resulting high CRI remains valuable. Lee et al. demonstrated this concept using fully chloride-based MAPbCl_3_ ST-PSCs, reporting a CRI of 95, an AVT of 72.5%, and minimal haziness [35]. A PCE of 0.68% was achieved by employing Pb(Ac)_2_ instead of conventional PbCl_2_ as the lead source, which improved precursor solubility and the resulting film quality.

Although tuning the halide composition readily yields the desired band gap and semitransparency, it also introduces new challenges related to lower photostability, high *V*_OC_ losses, and poorer morphological quality. The first issue will be detailed further in a dedicated section about the stability. Wide-band-gap perovskite devices are theoretically capable of generating a high *V*_OC_ owing to their larger band gap. However, the experimentally achieved *V*_OC_ in ST-PSCs is often lower than expected. One of the major issues arises from the energetic misalignment between the wide-band-gap perovskite and the charge transport layers, leading to a mismatch between the internal quasi-Fermi level splitting (QFLS) and the external *V*_OC_ and, consequently, enhanced interfacial non-radiative recombination [106]. An effective strategy to mitigate these losses is by using surface passivation. For example, PMACl passivates the perovskite surface defects by filling the halide/organic cation vacancies and coordinating with undercoordinated Pb^2+^. This interaction induces the formation of a wide-band-gap 2D (PMA)_2_PbCl_4_ layer that establishes type-I band alignment, which serves as an electron-blocking barrier at the perovskite/spiro-OMeTAD interface (Fig. 3e). Consequently, interfacial non-radiative recombination is effectively suppressed, enabling ST-PSCs to achieve a PCE of 18.6% [36].

Increasing the perovskite band gap through solution processing may also compromise film morphology because of the changes in crystallization kinetics [52, 53]. For instance, Br-rich perovskites exhibit low formation energies, which induce rapid and non-uniform nucleation during solution processing, leading to uncontrolled crystal growth [15, 101]. As a result, mixed-halide wide-band-gap perovskite films commonly show pinholes, defects, and inhomogeneous compositions, accompanied by poor crystallinity, high trap densities, and irreversible microstrain [15]. In addition, the ionic size mismatch between I^−^ (2.20 Å) and Br^−^ (1.96 Å) induces internal compressive stress during pre-crystallization and annealing, giving rise to surface wrinkles in the films [107]. Defects tend to accumulate in the wrinkle valleys, where they shorten the carrier diffusion length, enhance non-radiative recombination, and accelerate phase segregation [108, 109]. These effects ultimately reduce both V_OC_ and PCE over time [110]. Modifying the solvent system offers an alternative route to modulate the crystallization kinetics. Lian et al. developed wide-band-gap perovskite films using a pure DMSO-based precursor combined with nitrogen gas quenching and DMSO-mediated solution aging (DMSA) treatment [15]. Compared to DMF-based systems, the higher viscosity of DMSO reduces the crystallization rate, allowing smooth film formation, preventing surface wrinkles, and enabling ST-PSCs to reach 17.61% PCE (Fig. 3f).

#### Microstructure Engineering

**Fig. 4:**
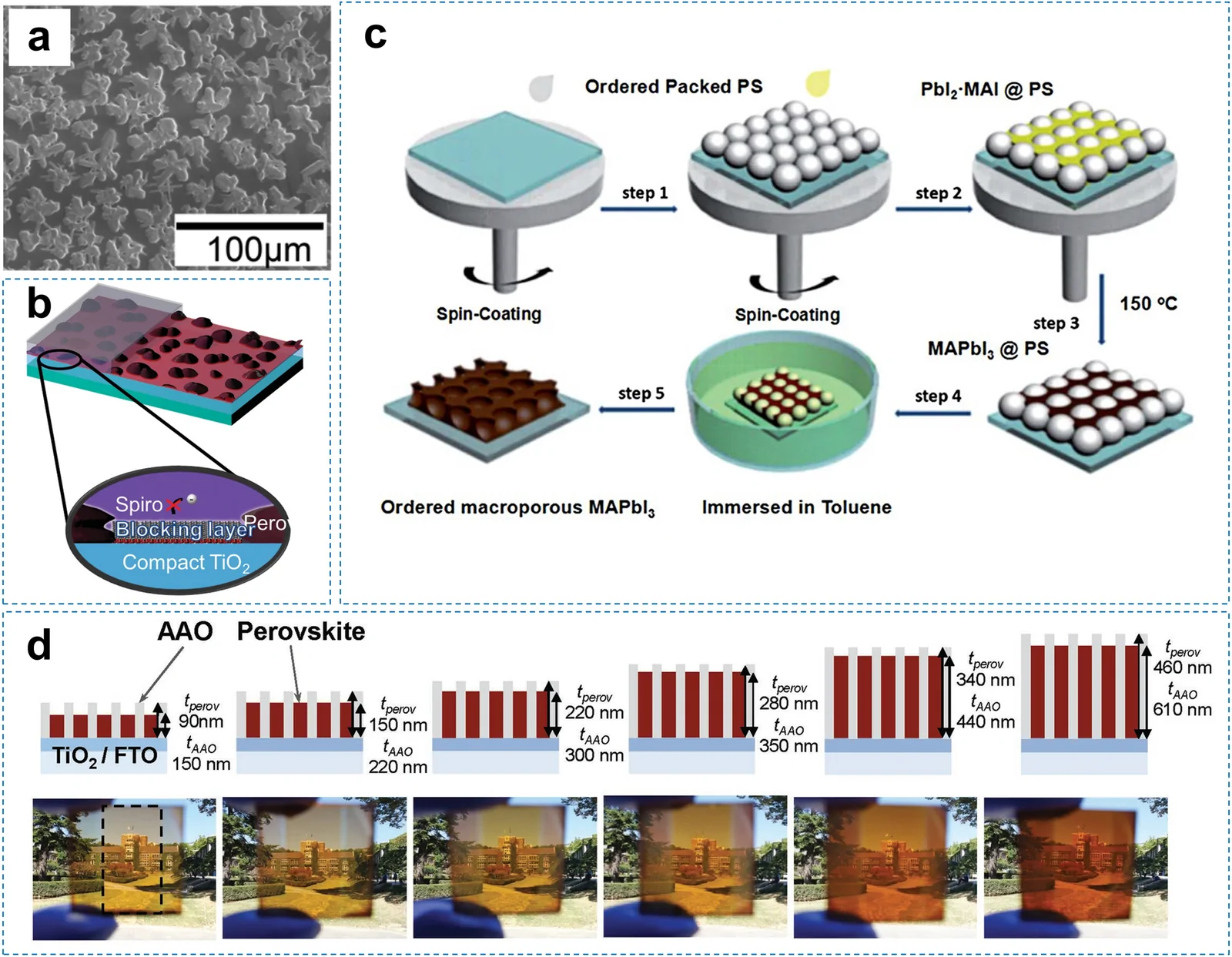
**a** SEM image of island-like perovskite layer. Reproduced with permission [42]. Copyright 2014, American Chemical Society. **b** Schematic diagram of the island-like perovskite sample containing siloxane as blocking layer. Reproduced with permission [43]. Copyright 2016, Wiley. **c** Fabrication process of macroporous perovskite layer using sacrificial polystyrene template. Reproduced with permission [44]. Copyright 2016, Royal Society of Chemistry. **d** Schematic illustration of perovskite layers grown within anodized Al_2_O_3_ (AAO) scaffolds of different thicknesses, along with corresponding photographs of the fabricated samples. Reproduced with permission [45]. Copyright 2016, Wiley

During the early development of ST-PSCs, partial transmission of light is achievable by deliberately reducing perovskite coverage in order to produce porous films. For example, Eperon et al. modified the precursor chemistry by preparing a solution with excess organic components dissolved in DMSO [42]. The surplus organic content slowed the perovskite crystallization, while the slow evaporation of DMSO further delayed the film formation, resulting in a discontinuous island-like perovskite layer that enabled an AVT of 28% (Fig. 4a). However, the presence of perovskite-free regions created shunt pathways due to direct ETL/HTL contact, limiting the PCE to only 5.2%. To address this issue, Hörantner et al. introduced alkyl-siloxane, which preferentially bound to the exposed TiO_2_ regions, forming a transparent blocking layer (Fig. 4b) [43]. This approach slightly improved the performance, yielding a PCE of 6.1% and an AVT of 38%.

Another strategy to control perovskite coverage is to grow the perovskite crystals inside a sacrificial layer of polystyrene microspheres. The polystyrene is then removed with toluene, leaving behind a well-ordered macroporous perovskite structure (Fig. 4c) [44]. Changing the size of the microspheres allowed the control of the AVT from 20% to 45%, with the best PCE of 11.7% obtained at 36.5% AVT. Meanwhile, Kwon et al. employed anodized Al_2_O_3_ nanopillars of varying lengths as a porous template to grow perovskite layer of different thickness (Fig. 4d) [45]. During the perovskite deposition, the spin speed was increased to prevent the formation of thick overlayer on Al_2_O_3_ and confine the perovskite within the porous structure. The spatial confinement of the template also reduced the crystallization rate by slowing the solvent evaporation, enabling upward crystal growth [35]. Since Al_2_O_3_ is relatively transparent, this approach enabled ST-PSCs with efficiencies of 8.5%–13.3% at AVTs of 45.4%–26.3% [45]. Although these approaches improved transmittance and reduced shunt pathways, the need for additional materials and complex synthesis may pose further challenges when scaling up the device fabrication.

The next strategy to control the semitransparent properties of PSCs is micropatterning, which involves spatially segmenting an opaque device into alternating regions of complete perovskite stack and transparent openings. This method has been successfully applied to Si- and thin-film-based PV technologies previously, demonstrating its universality [111–113]. In practice, once the opaque PSC is fully fabricated, selected areas are removed using mechanical scribing or laser ablation, yielding translucent ST-PSCs with nearly color-neutral light transmission. Moreover, micropatterning offers design flexibility, since the architecture can be tailored by adjusting the shape and distribution of the removed areas. This enables direct control over transparency level, gradient optical effects, visual perception, and aesthetic qualities to suit specific application requirements.

The performance of translucent ST-PSC is mainly influenced by the patterning method, the pattern design, and the perovskite stack. Optimal performance is achieved when the reduction in PCE stems only from the decrease in J_SC_, which is proportional to the active area removed during patterning. In this translucent ST-PSC concept, the opaque region accounts for only a fraction of the aperture area. Thus, the performance of ST-PSC can also be evaluated using the “opaque-area-PCE,” which normalizes the output power to the absorbing (opaque) portion of the aperture area [46]. This metric enables direct comparison between unpatterned opaque PSC and patterned translucent ST-PSC. The optimization of performance and stability in translucent ST-PSCs follows similar strategies as in fully opaque devices, with the main distinction being the additional optimization required for the micropatterning process.

**Fig. 5:**
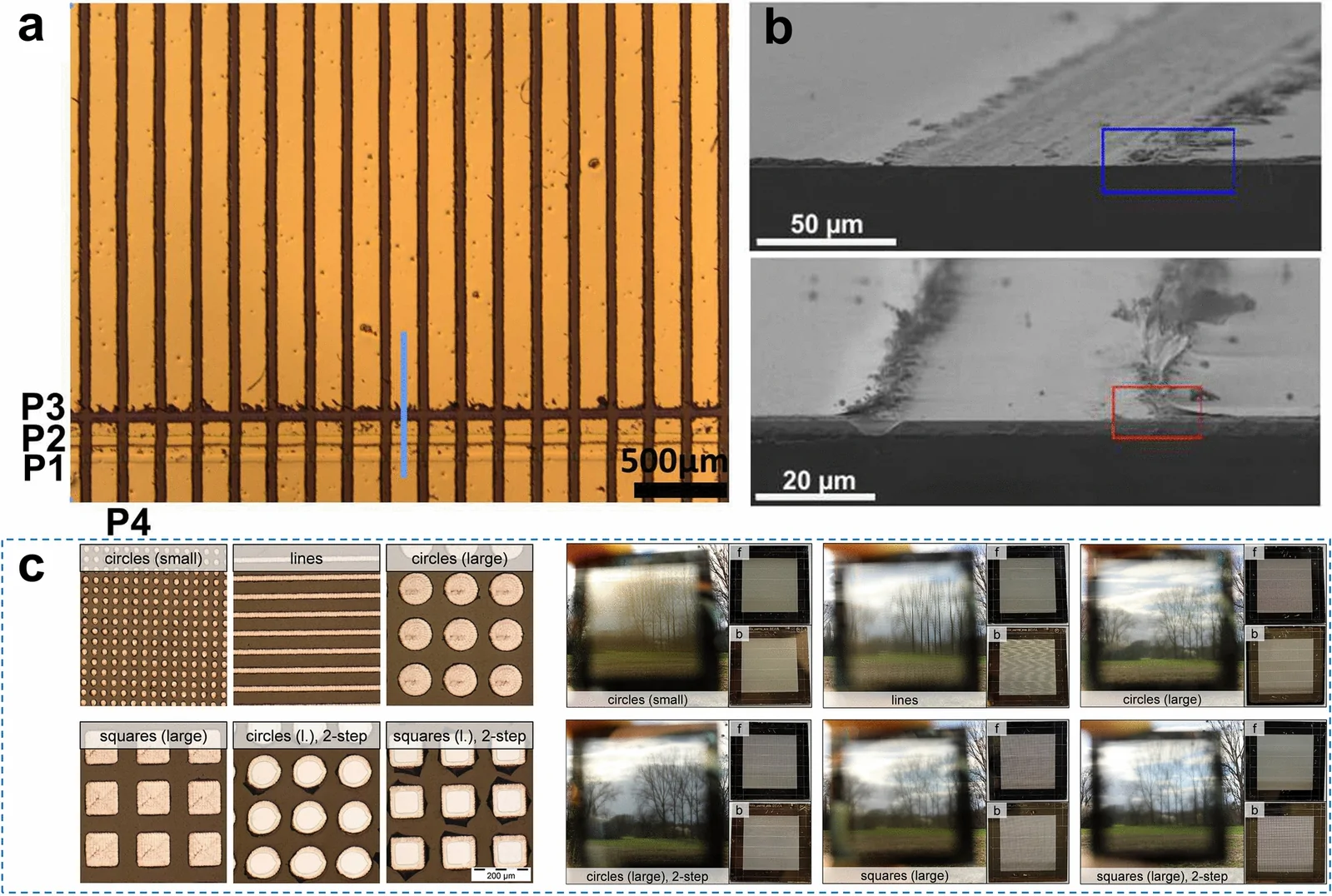
**a** Microscopic image of the translucent ST-PSC showing the patterned regions where the material was removed. **b** Cross-sectional SEM images of translucent ST-PSC with area removed via (top) mechanical scribing and (bottom) laser pattern. Reproduced with permission [46]. Copyright 2018, Royal Society of Chemistry. **c** Microscope images of translucent ST-PSCs with laser-scribed transparent areas of different shapes, together with the corresponding view-through, front-side, and back-side images of translucent submodules fabricated using these transparent area designs. Reproduced under terms of the CC-BY license [47]. Copyright 2023, The Authors, published by Royal Society of Chemistry

One of the earliest translucent ST-PSC was reported by Rakocevic et al., where around 10%-50% aperture area of 4 cm^2^ opaque devices was removed using mechanical or laser scribing (Fig. 5a) [46]. Both approaches, however, present certain risks that can compromise device integrity and performance. Mechanical patterning often causes irregular lateral damage from chipping, whereas laser patterning can lead to delamination of the top metal contact and Spiro-OMeTAD due to thermal effects, since these layers have weak adhesion to the underlying perovskite (Fig. 5b) [114]. Despite these issues, both methods yield comparable device performance, with laser patterning offering advantages in speed, precision, and flexibility of design. Although the PCE reduced from 13.1% to 5.4% with increasing AVT from 7% to 37% due to area removal, the opaque-area-PCE values remained similar (i.e., ~ 13%) for transparent area up to ~ 30%. This indicates that micropatterning strategy can easily alter the transparency level without affecting the original power output. However, the opaque-area-PCE obviously reduced once the transparent area exceeds ~ 40% primarily due to the increased series resistance caused by the patterning process.

Meanwhile, Ritzer et al. showed that laser scribing can produce unique translucent ST-PSCs with different micropatterns, including lines, circles, and squares of various sizes (Fig. 5c) [47]. They introduced a two-step laser scribing process in which the first step removes functional layers at low fluence, followed by a second step at higher fluence applied to a slightly smaller area to ablate the ITO. This approach not only minimized electrical damage to the functional layers caused by the strong laser pulses, but also enhanced the overall optical quality. Devices with additional ITO ablation exhibited superior clarity, achieving AVTs up to 44% with almost neutral CRI of 97 and only 3% haze. Among the designs, square-patterned devices delivered the best optical performance. The developed approach was also applied for the first time to tandem device based on 2 T perovskite–perovskite subcells, yielding PCEs of 11.1% at 31% AVT.

#### Other Strategies

**Fig. 6:**
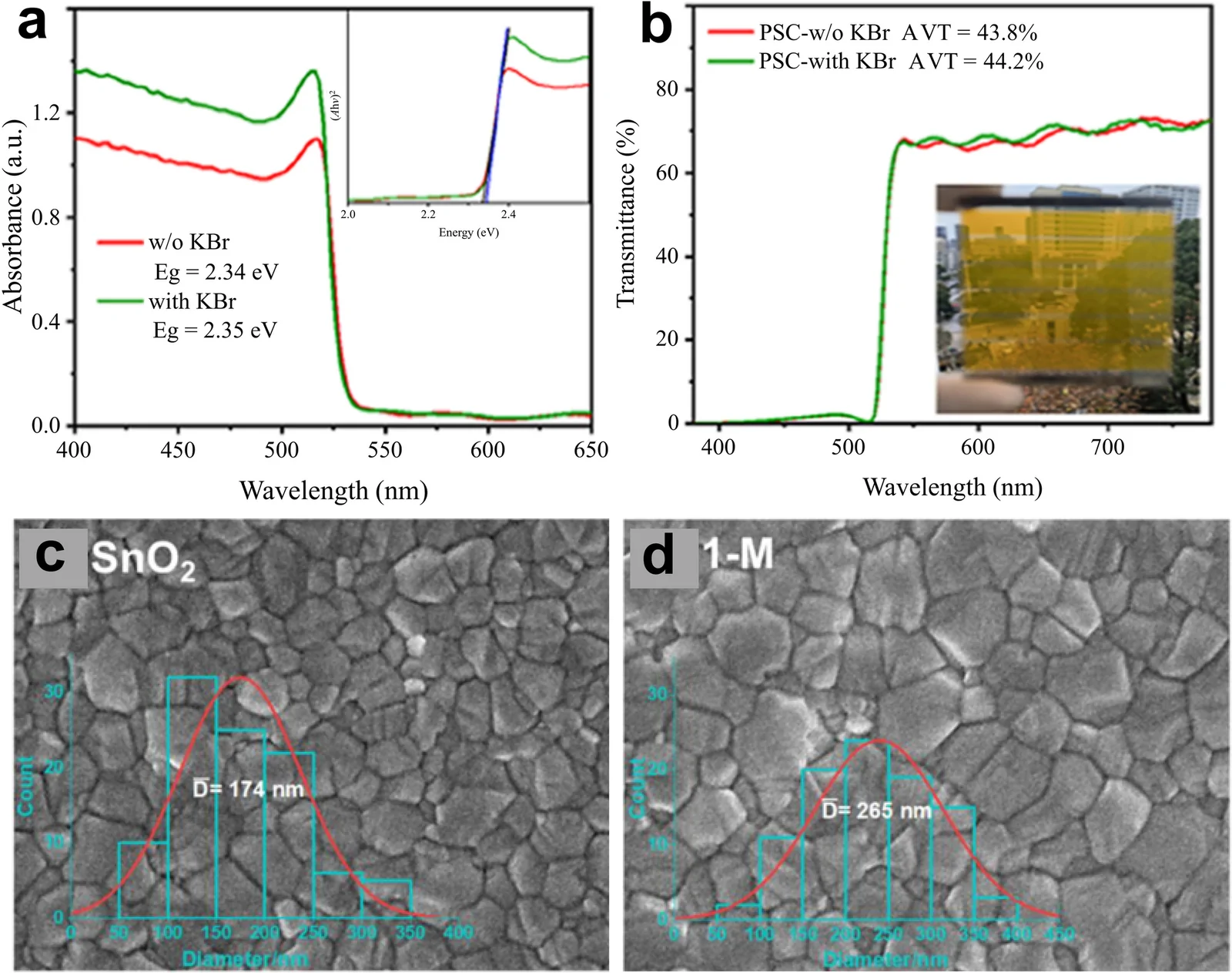
**a** Absorption (inset Tauc plot) and **b** transmittance spectra (inset photo) of CsPbBr_3_ films with and without KBr doping. Reproduced with permission [82]. Copyright 2024, American Chemical Society. SEM images of perovskite films deposited **c** on SnO_2_ and **d** on MXene interlayer at the buried interface. Reproduced with permission [84]. Copyright 2023, American Chemical Society

Generally, many strategies developed for opaque perovskite devices can be adapted to enhance the quality of perovskite films and reduce defects in the devices, thereby improving the performance of ST-PSCs. These strategies primarily involve introducing dopants or incorporating additional functional layers at the bottom or top surfaces of the perovskite film. For instance, doping a small amount of KBr into vapor-deposited CsPbBr_3_ films improved perovskite crystallinity, thereby enhancing charge extraction and increasing the PCE from 5.89% to 7.28%. Although the band gap of the doped perovskite film widened slightly by approximately 0.01 eV, the absorption of sub-band-gap light was obviously enhanced owing to better crystal quality (Fig. 6a). On the other hand, the transmittance of over-the-band-gap light was unaffected and the AVT remained high at 44% (Fig. 6b) [82]. Besides, introducing an interlayer such as 2D MXene at the buried interface can modulate perovskite crystallization and promote the growth of larger grains with reduced defect density (Fig. 6c, d). The resulting high-quality perovskite not only suppresses interfacial recombination but also mitigates bulk recombination due to the reduced number of grain boundaries, thereby enhancing the J_SC_ and *V*_OC_. This improvement compensates for efficiency losses associated with the thin absorber layer, yielding an ST-PSC with approximately 15% PCE and 27% AVT [84].

### Charge Transport Layers

In addition to their primary role in charge extraction, the charge transport layers (ETL and HTL) in ST-PSCs must exhibit minimal parasitic light absorption. This requirement is critical because parasitic absorption in these layers neither contributes to photocurrent generation nor supports transparency, thereby impairing device visibility and diminishing the performance of advanced PV configurations such as tandem and bifacial solar cells. Materials with wide band gaps and the ability to form uniform ultrathin films are therefore highly desirable for use as charge transport layers.

**Fig. 7:**
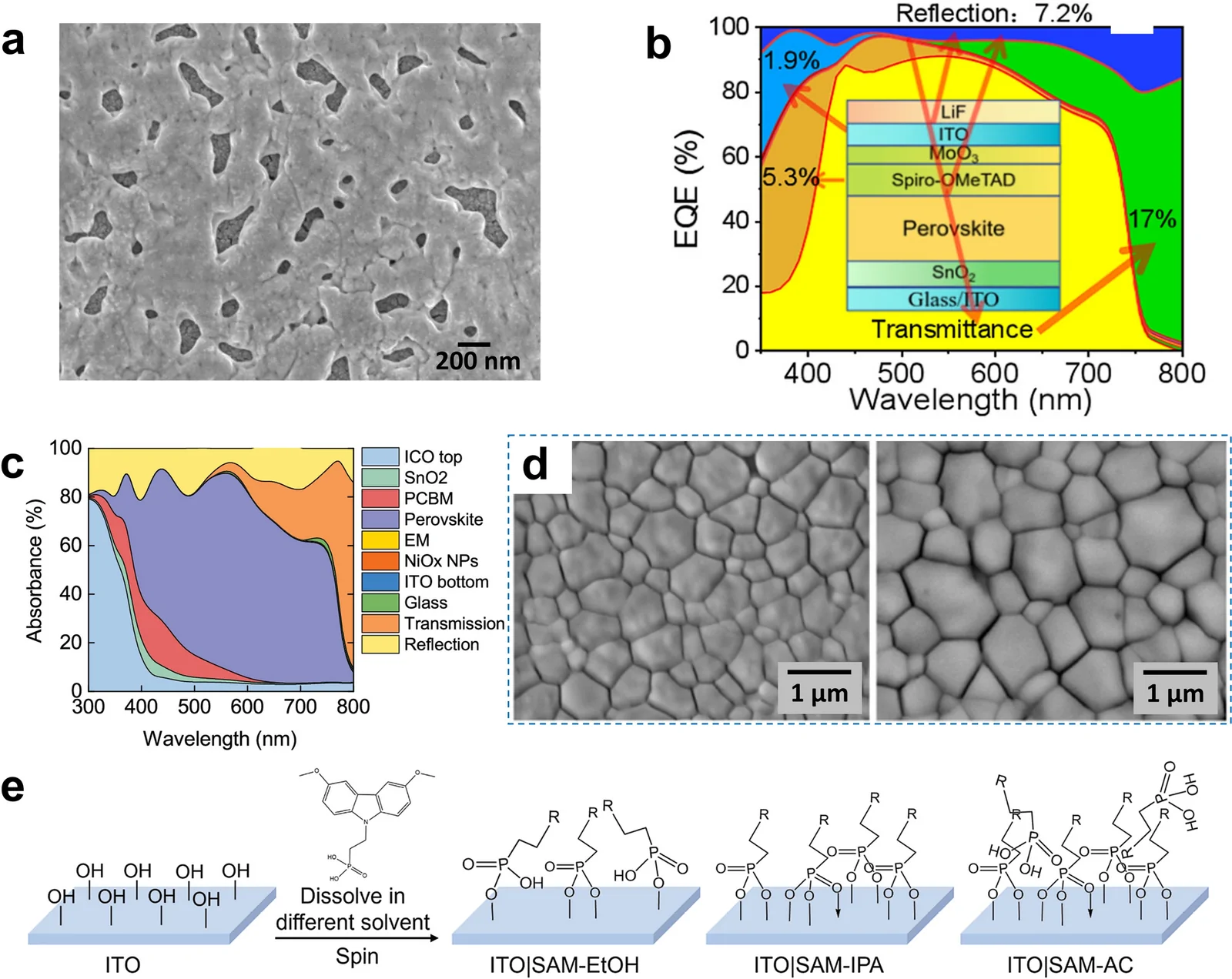
**a** SEM image of CsPbI_3_-based perovskite with 180-nm-thick capping layer on mesoporous TiO_2_ scaffold. Reproduced under terms of the CC-BY license [48]. Copyright 2025, The Authors, published by American Chemical Society. **b** Optical loss analysis of the ST-PSC showing parasitic absorption by spiro-OMeTAD. Reproduced under terms of the CC-BY license [115]. Copyright 2025, The Authors, published by Wiley. **c** Optical loss analysis of the ST-PSC showing parasitic absorption by PCBM. Reproduced under terms of the CC-BY license [80]. Copyright 2024, The Authors, published by American Chemical Society. **d** SEM images of FAPbBr_3_ perovskite films deposited on (left) ITO/SnO_2_ and (right) ITO/SnO_2_/KCl. Reproduced with permission [49]. Copyright 2024, American Chemical Society. **e** Schematic illustration of MeO-2PACz molecular packing formation on ITO prepared using different solvents. Reproduced with permission [50]. Copyright 2024, Elsevier

In devices employing a mesoporous-structured ETL such as TiO_2_, the perovskite layer cannot be made as thin as in planar architectures to achieve the desired transparency. This limitation arises because the perovskite must completely infiltrate the mesoporous scaffold while simultaneously forming a continuous capping layer on its surface to prevent direct contact between the ETL and HTL. As a result, the perovskite layer must always be slightly thicker than the mesoporous layer, inherently limiting the achievable transparency. Furthermore, maintaining uniform infiltration and complete surface coverage throughout the mesoporous network remains a considerable challenge. For instance, Fig. 7a illustrates the formation of a CsPbI_3_-based perovskite capping layer on mesoporous TiO_2_. Although the capping layer is approximately 180 nm thick, several uncovered regions are still visible, indicating incomplete surface coverage [48]. Hence, planar architectures, which readily facilitate the formation of a compact and fully covered perovskite film, are generally more favorable for achieving high transparency and uniformity in ST-PSCs [59].

Both conventional n–i–p and inverted p–i–n architectures show strong potential for achieving high PCE and AVT. In n–i–p ST-PSCs, TiO_2_ (bandgap ~ 3.2 eV) and SnO_2_ (bandgap ~ 3.8 eV) are commonly employed as ETLs due to their wide bandgaps and excellent transparency, with SnO_2_ offering a slight advantage [92, 116]. In p–i–n devices, the development of self-assembled monolayer (SAM)-based HTLs such as 2PACz, MeO-2PACz, and Me-4PACz has been particularly beneficial, not only for efficient hole extraction but also for improving transparency owing to their ultrathin nature (thickness < 2 nm) [117]. However, alternative materials for organic-based charge transport layers require further exploration, as conventional materials exhibit relatively high parasitic absorption in both the visible and NIR regions. For instance, the parasitic absorption of spiro-OMeTAD-based HTL accounts for at least 5.3% of the total incident light (Fig. 7b), and this loss increases with increasing spiro-OMeTAD thickness [115]. Likewise, approximately 5.7% of light incident from the rear side is parasitically absorbed by PCBM-based ETL, which is detrimental to bifacial operation (Fig. 7c) [80].

Many strategies developed for optimizing the ETL and HTL in opaque PSCs are also applicable to ST-PSC configurations. For instance, KCl has been employed to heal oxygen vacancy defects in the SnO_2_ layer, thereby enhancing electron extraction at the buried interface and improving the performance of opaque PSCs [118, 119]. A similar observation was reported by Jafarzadeh et al. for ST-PSC, where the introduction of KCl onto the SnO_2_-based ETL reduced surface defects on SnO_2_ and promoted the formation of larger grains of FAPbBr_3_ wide-band-gap perovskite (Fig. 7d) [49]. This structural improvement facilitates more efficient charge transport within the device, resulting in higher J_SC_ and FF, ultimately enabling a flexible ST-PSC with a PCE of 6.8% and an AVT of 55%.

Improving the quality of the HTL in inverted structures is essential not only for efficient charge extraction but also for controlling the buried interface properties of wide-bandgap perovskites. Hou et al. achieved an ST-PSC with 19.1% PCE and an 17.8% AVT by optimizing the molecular packing of MeO-2PACz on ITO [50]. This was accomplished by replacing ethanol, a commonly used solvent, with isopropanol (IPA). During fabrication, ethanol evaporates rapidly due to its low saturation vapor pressure, which limits the MeO-2PACz adsorption on the substrate. In contrast, IPA evaporates more slowly, enabling effective chemical adsorption and compact layer formation of MeO-2PACz (Fig. 7e). Such film quality also improves the compactness of perovskite grains at the buried interface. However, using a very slow-evaporating solvent like acetone leads to mere physical adsorption and mechanical stacking of MeO-2PACz, resulting in an overly thick layer with poor surface coverage.

### Transparent Conductive Electrodes

The transparent electrode is the key component that distinguishes ST-PSCs from conventional opaque PSCs, as it replaces the metallic rear contact to enable optical transparency while maintaining sufficient electrical conductivity. In conventional PSCs, the rear electrode typically consists of a highly conductive metal such as Au, Ag, or Cu with thickness between 80 to 100 nm. In contrast, ST-PSCs employ transparent conductive materials as rear electrodes such as highly doped oxide semiconductors or so-called transparent conductive oxides (TCOs), ultrathin metals, dielectric/metal/dielectric (DMD) structures, and carbon-based materials [120, 121]. In general, high optical transmittance particularly in the photoactive region and low sheet resistance are the two primary criteria for transparent electrodes to achieve optimal performance [122–124]. When the ST-PSC is coupled with a secondary device beneath it, such as in tandem solar cells or PV–PEC systems, high transmittance in the NIR region becomes equally important to ensure sufficient light absorption by the bottom device. Furthermore, an ideal transparent electrode should exhibit high chemical and thermal stability, strong interfacial adhesion, process compatibility with underlying layers, cost-effective fabrication and materials, and good mechanical flexibility for flexible device applications [125]. In some device configurations, transparent electrodes can also exhibit antireflective properties, offering an additional performance advantage [56]. Optimizing the thickness of the transparent electrode is also essential to balance optical transparency and lateral charge transport, as excessively thick films amplify parasitic absorption, whereas overly thin films increase electrical resistance [126].

#### TCO

TCOs are widely used in ST-PSCs due to their high optical transparency, excellent electrical conductivity, strong film adhesion, and well-established deposition technologies. Among them, fluorine-doped tin oxide (FTO) and indium tin oxide (ITO) coated on glass are commercially available and have become standard substrate materials for both opaque and semitransparent PSCs. However, FTO is unsuitable as the rear contact for ST-PSCs because its fabrication by atmospheric-pressure chemical vapor deposition (APCVD) involves high temperatures and reactive chemicals that can damage perovskite and organic charge transport layers [127], unless a thermo-compression method is applied. Therefore, ITO, which can be deposited by low-temperature sputtering technique, remains the preferred choice for rear electrodes in ST-PSCs. Its high carrier concentration (> 10^21^ cm^−3^) [61] and large band gap (~ 3.6 eV) [128] enable excellent electrical conductivity while maintaining high optical transmittance in the visible region.

Although ITO is widely used owing to its favorable properties, its high free carrier density leads to strong intraband absorption, resulting in significant parasitic loss of NIR photons [129]. While lowering carrier density can improve transparency, it also reduces lateral conductivity of ITO, which is already much lower than that of metal electrodes. Poor conductivity is particularly detrimental to the FF and PCE of the large-area devices. To mitigate this, a metal grid or finger layer can be deposited on top of the TCO, but wide grid lines may cause excessive J_SC_ losses due to shading effect [54]. Alternatively, using thinner TCO layer could enhance the transparency but this feature increases sheet resistance and degrades device performance [77]. While maintaining a balance between electrical conductivity and optical transparency is crucial, the development of TCOs material that can simultaneously offer high transmittance and excellent conductivity remains a major research priority.

**Fig. 8:**
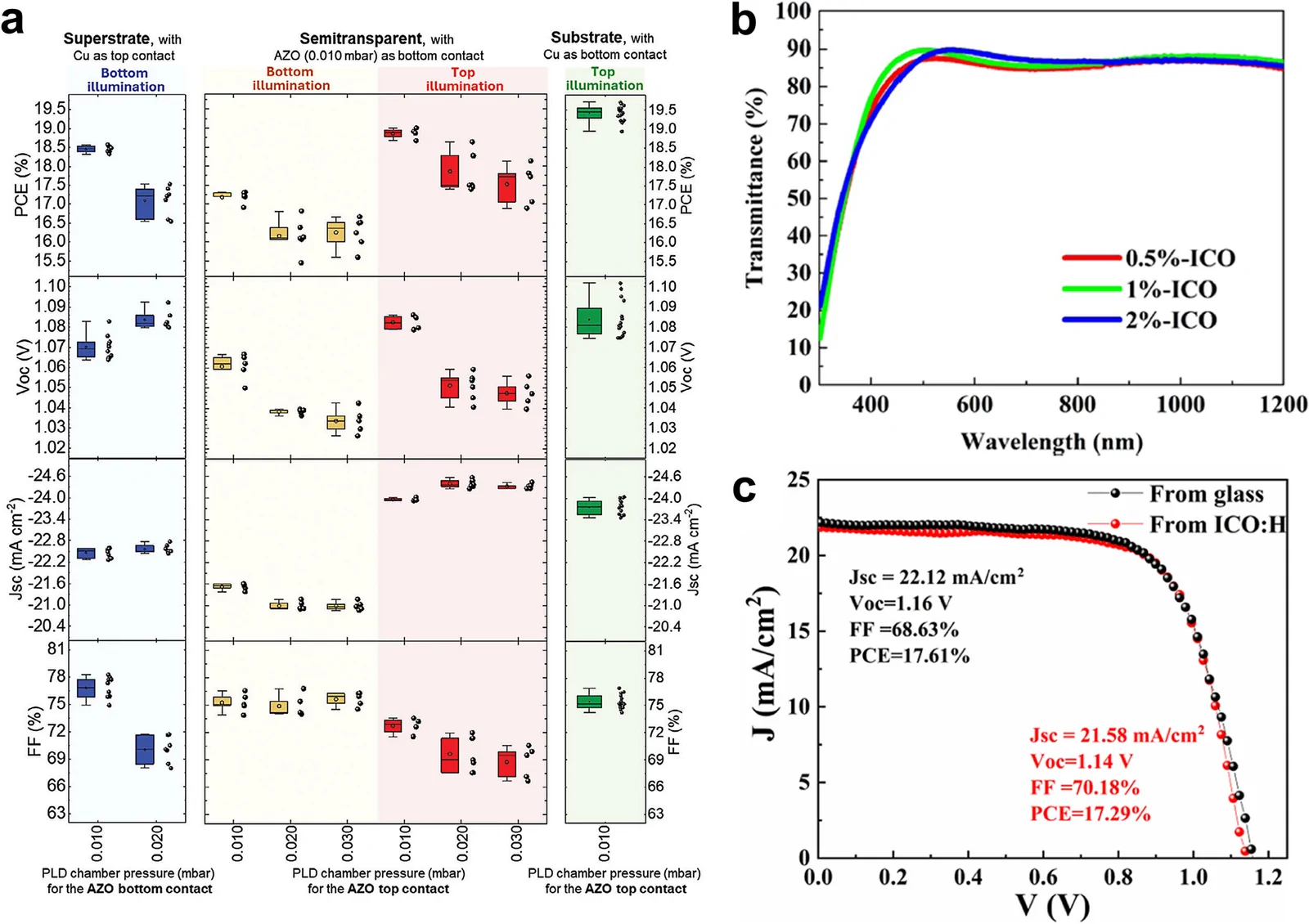
**a** Statistical distribution of the PV performance of PSCs employing PLD-deposited AZO as the top electrode (superstrate configuration), bottom electrode (substrate configuration), or both electrodes (semitransparent configuration). Reproduced under terms of the CC-BY-NC-ND license [55]. Copyright 2024, The Authors, published by Wiley. **b** Transmittance spectra of 100-nm-thick ICO films on glass with varying concentrations of cerium doping. Reproduced with permission [56]. Copyright 2023, American Chemical Society. **c** J–V curves of ST-PSCs with ICO:H electrode measured from the front (glass side) and back (electrode side). Reproduced with permission [57]. Copyright 2024, Elsevier

Aluminum-doped zinc oxide (AZO) is another promising TCO for ST-PSCs and is commonly used in CIGS solar cells due to its superior NIR transparency and cost-effectiveness [124]. However, its electrical conductivity is low without annealing at ≥ 200 °C [130], a temperature that can damage the underlying perovskite. Encouragingly, AZO-based devices also exhibit better stability than metal-based counterparts [131], although comparable stability to ITO-based devices [55]. More importantly, avoiding reliance on scarce indium can prevent from potential price fluctuations [132]. Recently, Reinders et al. successfully fabricated highly conductive and homogeneous AZO electrodes at room temperature using PLD, achieving sheet resistances of ~ 25–55 Ω sq^−1^ [55]. PLD offers precise stoichiometric control, which is important for maintaining the optimal Al ratio, whereas its high-energy deposition enhances crystallinity and film density, reducing defects and improving both conductivity and stability. AZO deposited via PLD can serve as the bottom electrode, top electrode, or both, with all configurations demonstrating impressive PCE (Fig. 8a). The maximum recorded PCE for ST-PSC reached 19.0%, matching that of ITO-based devices. AZO-based ST-PSC also exhibited stability comparable to ITO-based devices, retaining approximately 80% of their initial efficiency after continuous heating at 85 °C in N_2_ for ~ 1800 h. Nevertheless, the industrial scalability of PLD remains under debate.

Enhancing carrier mobility rather than increasing carrier density is an alternative yet effective strategy to achieve high-conductivity TCO without causing excessive NIR absorption. To implement this, cerium-doped indium oxide (ICO) containing 0.5%–2% cerium has been developed as a rear transparent electrode due to its intrinsically high carrier mobility. The ICO film exhibits a band gap of 3.53–3.56 eV and transmittance exceeding 86% over a broad wavelength range (400–1200 nm) (Fig. 8b). A device employing a 100-nm-thick 1% cerium-doped ICO layer with a MoO_*x*_ buffer achieved a PCE of about 19% [56]. Building on this approach, Che et al. fabricated ST-PSCs using hydrogen-doped ICO (ICO:H) electrodes without a buffer layer, attaining a PCE above 17% when measured from both sides (Fig. 8c). This performance was attributed to the non-damaging reactive plasma deposition process and the defect passivation by hydrogen in amorphous ICO, which enhanced Hall mobility, reduced carrier density, and improved light transmission [57]. The ST-PSCs retained ~ 70% of their initial efficiency after 700 h of dark storage at room temperature in N_2_, comparable to the commonly employed ITO.

#### Metal-Based Electrode

Generally, metal films can still maintain adequate electrical conductivity, good ductility, and high transparency to visible light when their thickness is below ~ 20 nm [133]. However, as the film becomes thinner, the sheet resistance increases significantly due to discontinuous morphology and enhanced electron scattering at grain boundaries and surfaces [134]. Forming a continuous ultrathin metal layer on a charge transport layer is often challenging because of the large surface energy mismatch between the two materials. This mismatch typically results in the formation of metal films with isolated islands structure during thermal evaporation, following the Volmer–Weber growth mode. To overcome this issue, ultrathin metal electrodes are commonly prepared using three strategies: (1) introducing a seed layer, (2) employing a dielectric–metal–dielectric (DMD) multilayer configuration, or (3) using nanowire structure.

**Fig. 9:**
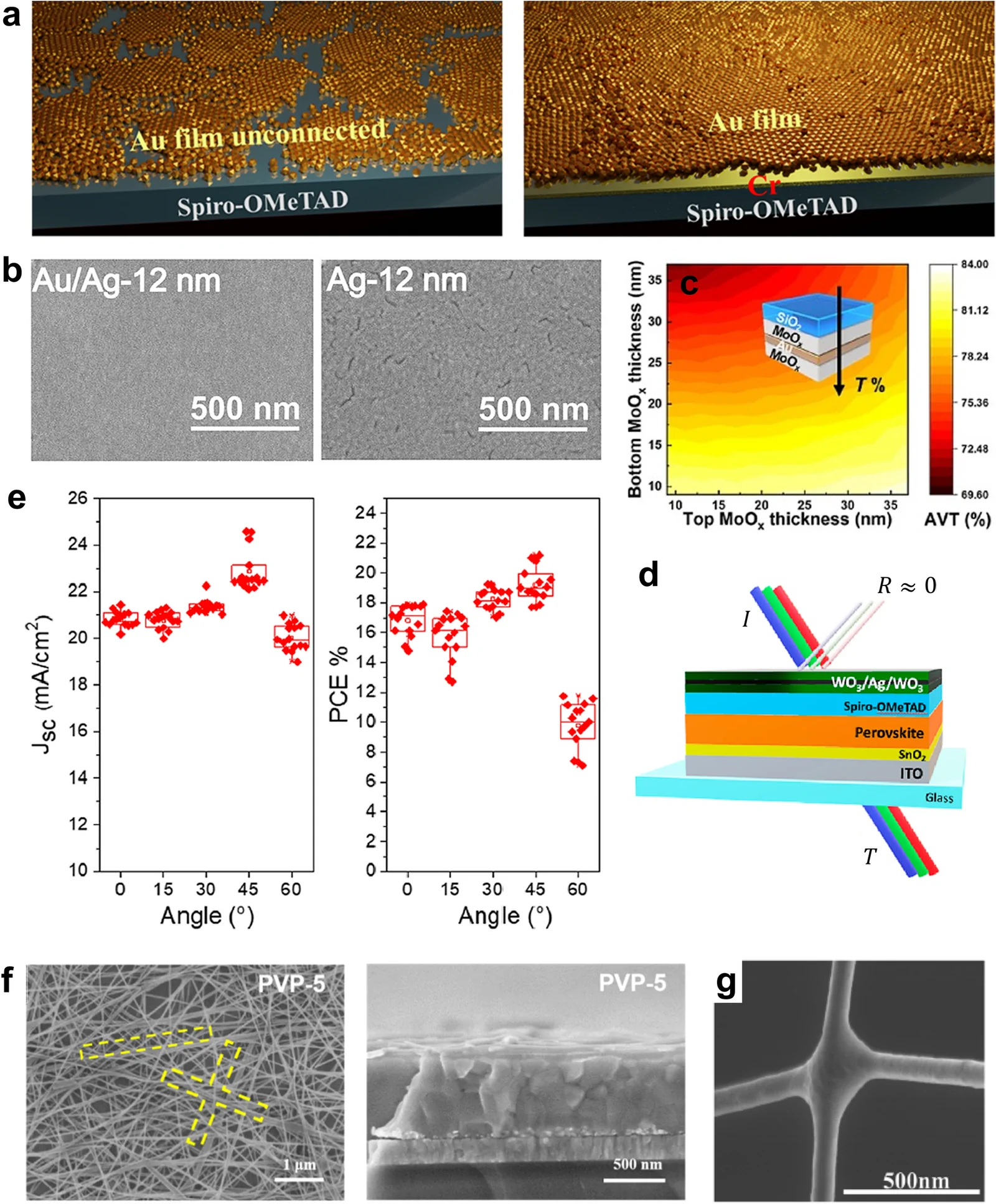
**a** Schematic comparison of ultrathin Au film formation on spiro-OMeTAD and on a Cr seed layer. Reproduced with permission [65]. Copyright 2021, Elsevier. **b** SEM image of 12 nm-thick Ag grown without and with 1-nm-thick Au seed layer. Reproduced with permission [66]. Copyright 2024, Elsevier. **c** Optical simulation of the AVT of MoO_x_/Au/MoO_x_ structures with varying top and bottom MoO_x_ layer thicknesses. Reproduced with permission [69]. Copyright 2023, Elsevier. **d** Schematic illustration of incident light entering the device at an angle, showing negligible reflectance. **e** Statistics of J_SC_ and PCE as a function of light incident angle for ST-PSC with WO_3_/Ag/WO_3_ electrode. Reproduced under terms of the CC-BY license [70]. Copyright 2024, The Authors, published by American Chemical Society. **f** Top-view and cross-sectional SEM images of Ag nanowires with PVP indicating improved network and adhesion. Reproduced with permission [67]. Copyright 2024, Elsevier. **g** SEM image of chemically welded Ag nanowire. Reproduced with permission [68]. Copyright 2023, American Chemical Society

The introduction of a seed layer can modify the growth behavior to the Frank–van der Merwe mode, enabling the formation of a uniform and continuous ultrathin film. For instance, Yang et al. deposited a 1-nm Cr seed layer prior to the growth of a 7-nm-thick Au layer and achieved a low sheet resistance of 16.3 Ω sq^−1^, which enabled an ST-PSC with an impressive PCE of 19.8% [65]. This improvement is attributed to the sufficient wettability provided by the Cr seed layer, which facilitates the formation of a compact ultrathin Au electrode on spiro-OMeTAD (Fig. 9a). Similarly, Cui et al. reported that a 12-nm-thick Ag film deposited without a seed layer exhibited numerous ravines and cracks due to its tendency to agglomerate into island-like structures (Fig. 9b) [66]. By introducing a 1-nm-thick Au seed layer, the Ag film became continuous and smooth because the seed layer effectively controlled the percolation threshold, enabling the formation of a dense Au/Ag bilayer. As a result, an ST-PSC with a PCE of 18% and an AVT of 17.97%, corresponding to a LUE of 3.23%, was successfully demonstrated.

Meanwhile, DMD electrodes consist of an ultrathin metal interlayer sandwiched between two dielectric layers with high refractive indices. Common dielectric materials are based on metal oxides such as MoO_3_ and WO_3_, while Ag and Au are frequently used as the metallic interlayers. The bottom dielectric layer facilitates efficient hole transfer from the HTL to the metal electrode due to its high work function [69], while simultaneously acting as a buffer that protects the underlying layer during electrode deposition. It also functions as a seed layer, as the low interfacial energy of the dielectric promotes strong adhesion with the metal, enabling the formation of uniform ultrathin metal films [69, 135]. Meanwhile, the top MoO_3_ layer serves as an optical spacer that controls metal reflectance, defines the transmission window, and provides antireflective protection for the underlying layers [81, 136]. The middle metal interlayer ensures high electrical conductivity for efficient charge extraction. The synergistic combination of materials in the DMD structure enhances optical transmittance, as the dielectric layers effectively suppress light reflection through the antireflective effect [137, 138]. In addition, DMD electrodes are usually fabricated by thermal evaporation rather than sputtering, which prevents high-energy particle damage to the underlying perovskite layer [139].

The key parameters governing DMD performance in ST-PSCs are the layer thicknesses and the dielectric–metal material combinations. Usually, the thickness of the bottom and top dielectric layer is similar. However, Kim et al. reported that asymmetric DMD electrodes with different dielectric layer thicknesses (MoO_*x*_ 15 nm/Au 10 nm/MoO_*x*_ 35 nm) exhibit higher optical transmittance than the symmetric ones (MoO_*x*_ 15 nm/Au 10 nm/MoO_*x*_ 15 nm) while maintaining high conductivity [69]. As shown in Fig. 9c, the AVT increased with a thicker top MoO_*x*_ layer and a thinner bottom MoO_*x*_ layer, as the former reduces parasitic absorption and the latter minimizes reflectance. Smoother surfaces in asymmetric DMD stacks further reduce visible light scattering and reflectance compared to symmetric stacks. Consequently, the asymmetric DMD structure enables a ST-PSC PCE of 11.3% at an AVT of 23.4%.

In practical operation, the angle of sunlight incident on ST-PSCs varies continuously throughout the day and across seasons. Therefore, it is crucial to develop transparent electrodes that maintain high light transmittance irrespective of the incident angle. To address this, the effect of dielectric layer thickness in WO_3_/Ag/WO_3_ electrodes was investigated by varying the WO_3_ thickness from 15 to 70 nm while keeping the Ag thickness constant at 14 nm [70]. A 40-nm-thick WO_3_ layer demonstrated negligible light reflection for incident angles between 0° and 45° (Fig. 9d), enabling the electrode to sustain a transmittance of approximately 78% across the visible region. This optical behavior closely resembles that of air. Additionally, the electrode demonstrated a low sheet resistance of about 10 Ω sq^−1^, enabling the fabrication of FACsPbI_3_-based ST-PSCs with a *J*_SC_ exceeding 20 mA cm^−2^ under various incident angles (Fig. 9e). The highest PCE achieved was 21.2% at an AVT of 15%.

Another approach to obtain transparent electrodes based on metal is by using silver nanowires (AgNWs), where a continuous, randomly distributed network of Ag forms a conductive and highly transparent layer. The solution processability of AgNWs makes them attractive for low-cost electrodes, but the loose junctions within the network and poor adhesion to the underlying layer often result in high resistance [140]. Blending AgNWs with a binder such as polyvinylpyrrolidone (PVP) can tighten the network and interfacial adhesion (Fig. 9f), enhancing charge transport and optical transmittance in ST-PSCs, as demonstrated by Fu et al. [67]. Using this strategy, an 18%-efficient ST-PSC with a high bifaciality factor of nearly 80% was achieved due to the high transparency from both illumination sides. Since AgNWs are only physically connected at their junctions, Bian et al. employed AgNO_3_ and polydopamine to chemically weld these contacts (Fig. 9g), which significantly improved conductivity [68]. Subsequently, reduced graphene oxide was coated onto the AgNWs to prevent oxidation by atmospheric oxygen. The resulting electrode exhibited a sheet resistance of 6.5 Ω sq^−1^ and 88% transmittance, along with excellent bending durability and environmental stability. When used as the rear electrode in an ST-PSC, the device achieved a PCE of 16.15% with an AVT of 9.8%.

#### Carbon-Based Electrode

Carbon-based materials, such as graphene and carbon nanotubes (CNTs), are promising candidates for transparent electrodes due to their hydrophobic nature and their ability to suppress metal ion migration, thereby contributing significantly to the excellent stability of ST-PSCs [75, 141, 142]. In addition, their composition of pure carbon atoms offers a cost-effective and sustainable alternative that address concerns over raw material supply. Carbon electrode also has excellent electrical conductivity, mechanical flexibility, and chemical stability. To date, two main types of carbon electrodes that have been explored for use as top contacts in PSCs are paste-type and film-type electrodes [143]. Paste-type electrodes, however, are opaque, making them unsuitable for semitransparent applications [76].

**Fig. 10:**
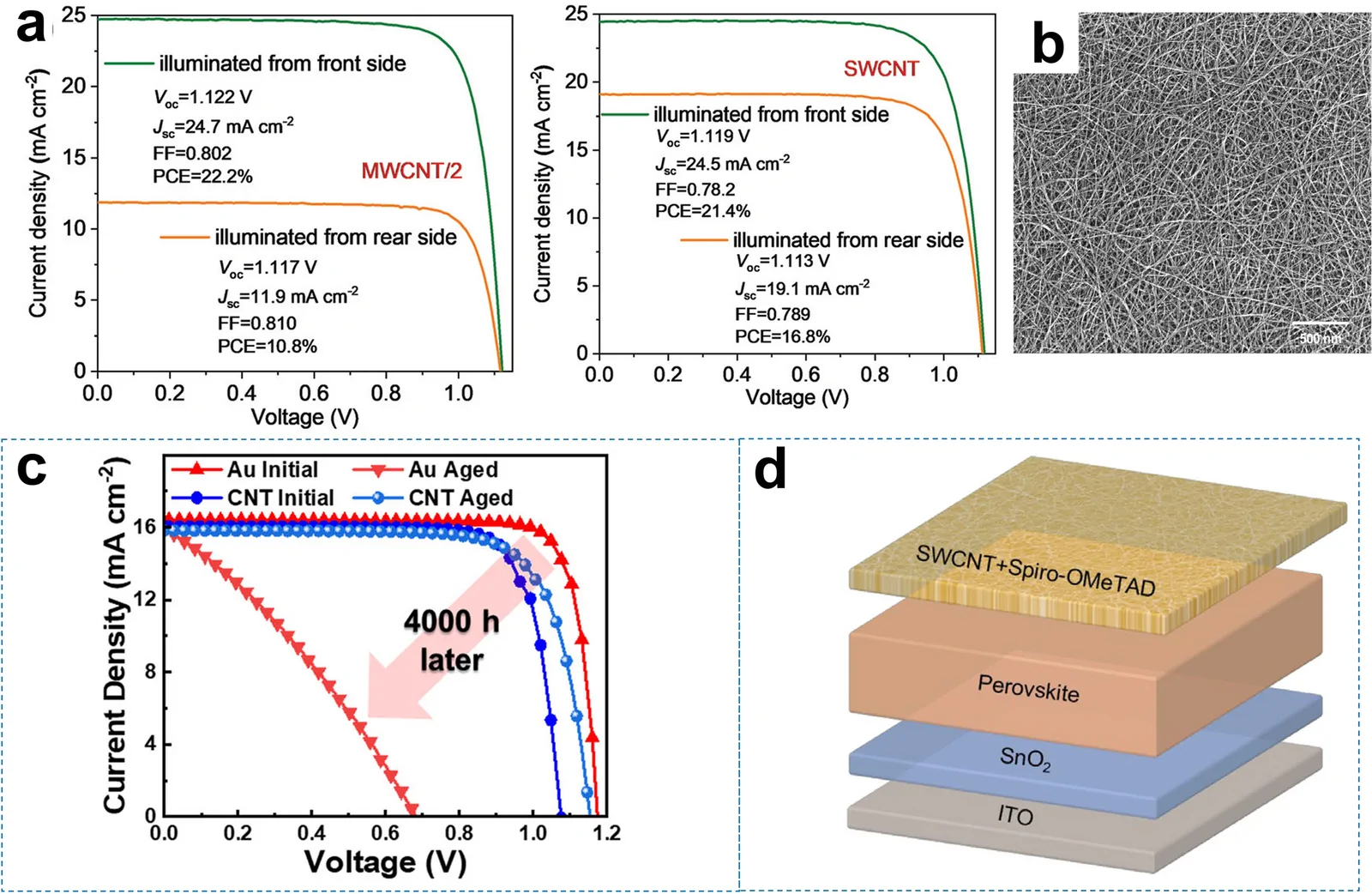
**a** J–V curves of ST-PSCs employing SWCNT or MWCNT transparent electrodes as measured from both the front and rear sides. Reproduced with permission [73]. Copyright 2022, Royal Society of Chemistry. **b** SEM image of SWCNT film prepared via FCCVD. Reproduced under terms of the CC-BY license [75]. Copyright 2024, The Authors, published by Springer Nature. **c** J–V curves of opaque PSC with Au electrodes and ST-PSC with CNT electrodes after aging for 4000 h. Reproduced under terms of the CC-BY license [76]. Copyright 2024, The Authors, published by Wiley. **d** Schematic diagram of the ST-PSC based on spiro-OMeTAD embedded SWCNT. Reproduced with permission [74]. Copyright 2025, Elsevier

Film-type carbon electrodes are more suitable for use in semitransparent devices since their thin structure allows sufficient light transmission. Graphene has been explored as transparent electrodes in the past few years, but the fabricated films often exhibit high sheet resistance [69, 144, 145]. In addition, the dry transfer process can introduce air pockets beneath the film, which deteriorates the quality of interfacial contact [146]. Meanwhile, CNTs such as single-walled (SWCNTs) and multi-walled (MWCNTs) variants have gained significant interest these days as it can produce highly efficient devices. SWCNTs, however, are generally more favorable than MWCNTs for ST-PSC because its single-walled structures enable higher optical transparency while maintaining comparable electrical conductivity. For instance, an ST-PSC with an MWCNT electrode achieved a front-side PCE of 22.2%, slightly higher than 21.4% for an SWCNT-based device [73]. However, when measured from the rear side, the SWCNT-based device showed much superior PCE of 16.8% compared to 10.8% for MWCNTs (Fig. 10a), due to the lower visible-light transmittance of MWCNTs. Therefore, MWCNTs are more suitable for single-junction devices where transparency is not critical, while SWCNTs are preferable for applications like bifacial and tandem solar cells.

The preparation of SWCNT films can be conducted via wet or dry process. The wet process involves dispersing and depositing SWCNTs onto target substrates, but this approach often introduces contaminants and structural defects that degrade film performance [74]. In contrast, the dry process, particularly the floating catalyst chemical vapor deposition (FCCVD) method, is gaining popularity, in which random SWCNT networks are directly formed on membrane filters at room temperature. These films can then be transferred onto perovskite layers without causing damage. Using this approach, SWCNT films with entangled, small-bundle structures have been successfully fabricated by Zhang et al. (Fig. 10b) [75]. The resulting films exhibited an AVT of around 85% and performed effectively as transparent electrodes on both sides of ST-PSC device, delivering bifacial PCEs of 18.54% and 18.22% for the front and rear sides, respectively.

Yoon et al. similarly employed FCCVD-synthesized CNT films to construct an inorganic ST-PSC with a PCE of 13.8% [76]. In their approach, the CNT electrode was first laminated onto the device, and the HTL was deposited afterward. This reversed sequence enabled the formation of a thicker HTL, which improved hole extraction. The carbon-based electrode also served as a moisture barrier, enabling the device to retain nearly 100% of its initial PCE for over 4000 h under ambient conditions without encapsulation, whereas the Au-based opaque device preserved only about 20% (Fig. 10c). Similar strategy has also been adopted by Li et al. where spiro-OMeTAD was intercalated into the SWCNT network (Fig. 10d), producing ST-PSC with PCE of 17.2% [74].

### Auxiliary Components

Beyond the critical functional layers, various strategies have also been employed to optimize auxiliary components, including anti-reflective coatings and photon-conversion materials, to further enhance the performance of ST-PSCs.

#### Anti-Reflective Coating

When light passes through different media, the differences in refractive index between each medium lead to Fresnel reflection, which results in significant loss of incident photons and consequently lowers the J_SC_ and overall PCE of the solar cells [147]. In ST-PSCs, reflection losses occur at both sides of the device, i.e., at the air/glass front interface and the air/transparent conductive oxide back interface. To minimize the undesirable reflection, antireflective coatings (ARC) are typically applied on both surfaces [148]. This is important not only to maximize the device efficiency, but also to suppress glare, minimize color distortion, and ensure a uniform optical appearance, which are particularly valuable for building-integrated applications.

**Fig. 11:**
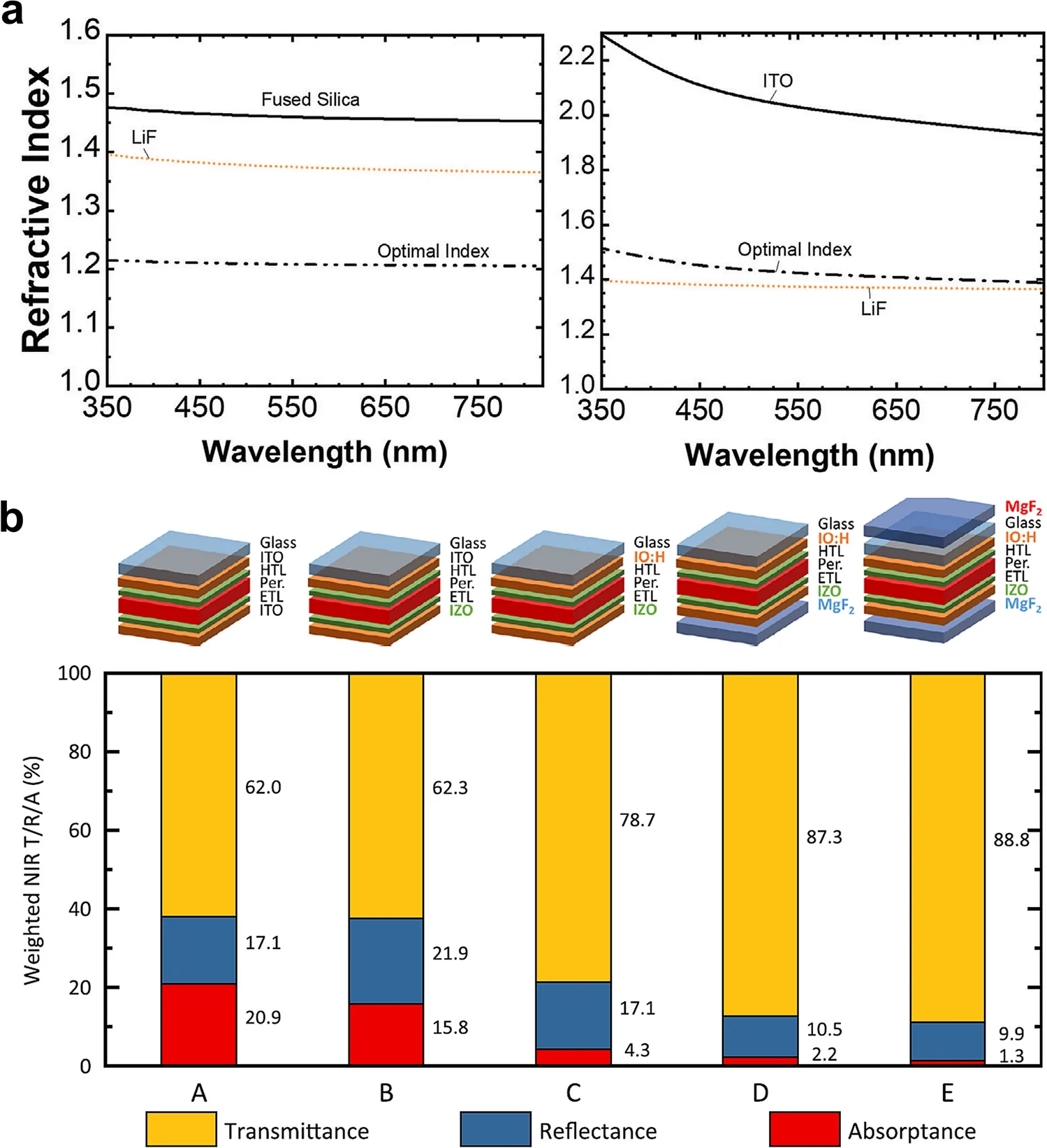
**a** Refractive index of several materials at the glass (fused silica)/air and ITO/air interfaces along with their respective optimal index. Reproduced under terms of the CC-BY-NC-ND license [78]. Copyright 2024, The Authors, published by Wiley. **b** Schematic of ST-PSC configurations with different TCOs, without ARC, with ARC on one side, and with ARC on both sides. The accompanying bar chart shows the distribution of NIR transmittance, reflectance, and absorbance for each configuration. Reproduced under terms of the CC-BY-NC license [77]. Copyright 2022, The Authors, published by Wiley

For an ARC layer to work effectively, its material must have a refractive index that matches well with the surrounding media. As shown in Fig. 11a, fused silica glass, which is commonly used as the substrate, has a refractive index of about 1.46, while ITO on the rear side of ST-PSCs has a refractive index of around 2.0. Accordingly, the optimal refractive index for a single-layer ARC is approximately 1.22 for the front side and 1.4 for the rear side [78, 149]. Materials such as LiF and MgF_2_, with refractive indices of 1.37–1.38 across the visible to NIR range [150], are therefore suitable for minimizing reflectance on both sides of the device. Rodkey et al. demonstrated that applying LiF together with Al_2_O_*x*_ as an ARC on ITO reduced reflectance losses to below 1%, which is particularly advantageous for bifacial operation, where light enters the ST-PSC from both sides [78].

ARC also plays a crucial role in ensuring efficient NIR transmission to the bottom devices, which is essential for achieving high overall performance in applications such as tandem solar cells and PV–PEC systems. Feeney et al. systematically investigated this effect and found that, depending on the device structure, around 17%–22% of NIR light was reflected in ST-PSCs without ARC (Fig. 11b) [77]. Depositing a 150-nm MgF_2_ layer on the IZO back contact reduced the reflection at the IZO/air interface to 10.5%, while adding another 125-nm MgF_2_ layer to the front glass further lowered the air/glass reflection loss by about 0.6%. These results highlight that ARC is not only vital for suppressing reflection losses and improving efficiency of the ST-PSC itself, but also for maintaining the high optical clarity required for real-world applications.

#### Photo Conversion

The light absorption of ST-PSCs can be enhanced by integrating up-conversion or down-conversion materials on the glass side where incident light enters, or inside the device as an additional layer [151]. Up-conversion materials absorb low-energy photons in the NIR range and convert them into higher-energy photons usable by the perovskite layer. In addition to improving PCE, up-conversion material can reduce direct transmission of certain NIR photons, marginally lowering the radiant heat passing through the device. In contrast, down-conversion materials absorb high-energy photons such as UV-light and re-emit them as lower-energy photons that align with the absorption spectrum of perovskite. This process could reduce the parasitic light absorption by ETL or HTL, increase photocurrent generation of ST-PSC, and minimize UV-induced degradation of perovskite layer. Some of these materials also exhibit minor anti-reflection properties, thereby increasing the light intensity entering the devices [80, 81]. These spectrum management strategies not only enhance light harvesting to increase J_SC_ and V_OC_, but also have potential to exceed the Shockley–Queisser limit without sacrificing transparency [152].

**Fig. 12:**
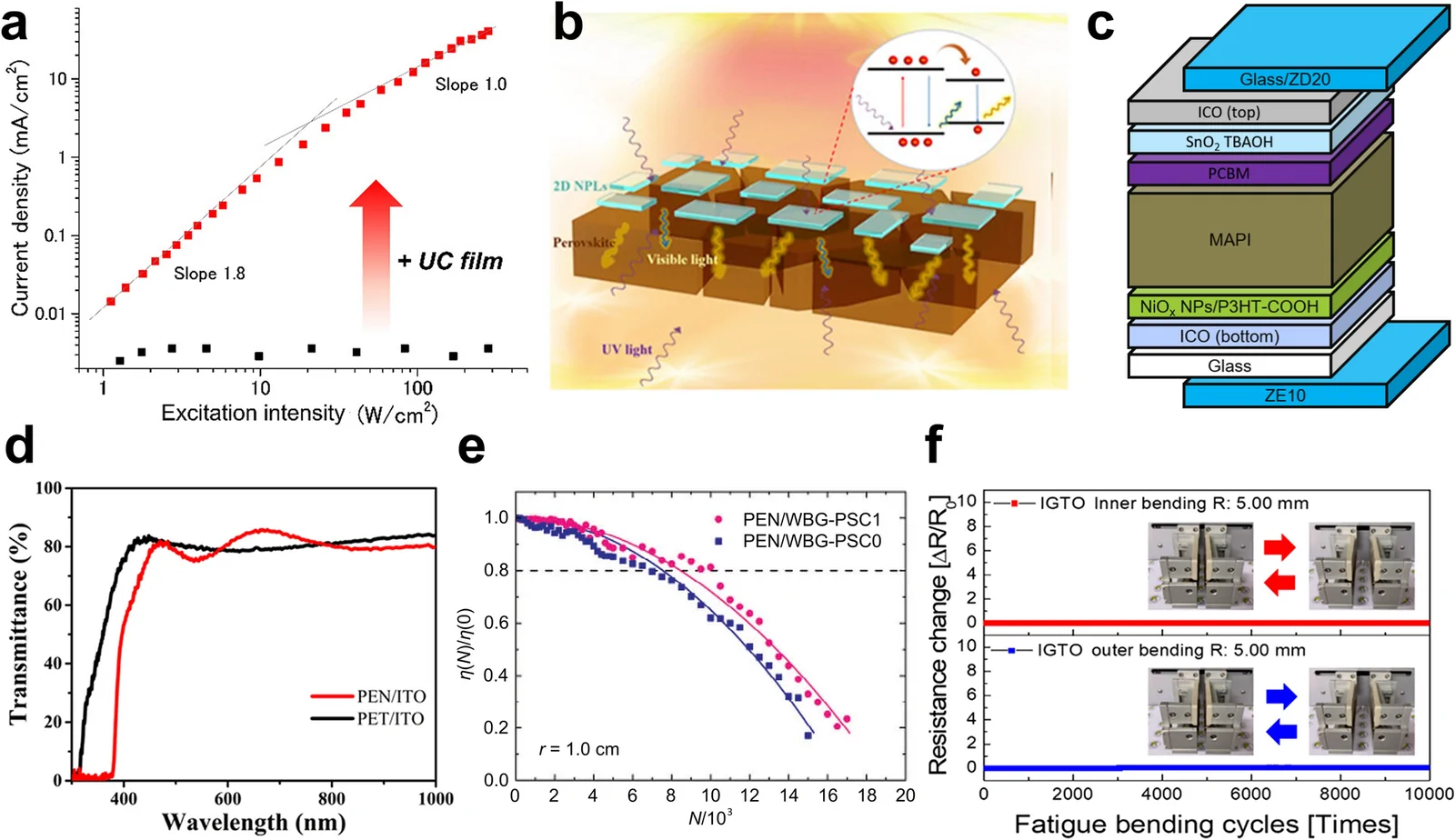
**a** Double-logarithmic plots of J_SC_ against excitation intensity (λ_excitation_ = 938 nm) for ST-PSC without or with rubrene-osmium complex up-conversion material. Reproduced with permission [79]. Copyright 2020, Wiley. **b** Schematic diagram of the mechanism of down-conversion effect by 2D-NPLs–PMMA composite. Reproduced under terms of the CC-BY license [81]. Copyright 2024, The Authors, published by Wiley. **c** Schematic diagram of ST-PSC with TPETPA-based (denoted as ZE10) and DPABA-based (denoted as ZD20) down-conversion layers at the front and rear side, respectively. Reproduced under terms of the CC-BY license [80]. Copyright 2024, The Authors, published by American Chemical Society. **d** Transmittance spectra of PEN/ITO and PET/ITO. Reproduced with permission [154]. Copyright 2023, Elsevier. **e** Bending durability test of flexible ST-PSC without and with polymer dopant. Reproduced with permission [155]. Copyright 2024, Wiley. **f** Resistance variation of IGTO on PET substrate over 10,000 cycles of fatigue bending. Reproduced with permission [156]. Copyright 2021, American Chemical Society

Kinoshita et al. verified this principle by placing rubrene combined with an osmium complex as up-conversion material behind the ST-PSCs. The device exhibited obvious photocurrent generation under 938 nm excitation (Fig. 12a), although the absorption of perovskite is typically limited to below 800 nm [79]. Yang et al. applied a down-conversion material based on 2D perovskite PEA_2_MA_3_Pb_4_Br_13_ nanoplatelets/poly(methyl methacrylate) (2D-NPLs–PMMA) composite layer in ST-PSCs [81]. The material absorbed UV–blue photons with energies above its bandgap (> 2.59 eV), which are not effectively absorbed by the bulk perovskite film. This process generated excitons confined within the 2D perovskite, causing quantum confinement effects and discrete energy states. The excitons then recombined radiatively, emitting visible photons that can be absorbed by the bulk perovskite, leading to an enhanced *J*_SC_ (Fig. 12b). The devices also exhibited higher *V*_OC_ and FF due to the passivation effect of the 2D perovskite and PMMA. Stability was also improved owing to the greater hydrophobicity of these components [153], which helps repelling water from entering the perovskite film. As a result, devices with PCEs of 14.26% and 10.65% with respective AVTs of 19.4% and 26.9% were obtained.

Different materials are suitable for different sides because of their unique capacity to optimize the absorption characteristics of ST-PSCs from either side. Glowienka et al. developed two types of down-conversion layers made of N,N-diphenyl-4-(1,2,2-triphenylethenyl)-benzenamine (TPETPA) mixed with polymeric binder and 4-(N,Ndiphenylamino) benzaldehyde (DPABA) mixed with polymeric binder with the aim to reduce UV optical losses [80]. The luminescence of the materials arises from the aggregation-induced emission, which amplifies the fluorescence of the dyes embedded in the binder. The study found that 350-nm TPETPA-based and 400-nm DPABA-based down-conversion films are suitable for the front and rear sides of the ST-PSC, respectively (Fig. 12c). With these down-conversion layers, each side of the ST-PSC exhibited enhanced PCE of approximately 0.4% absolute value.

### Flexible ST-PSC

ST-PSCs can also be fabricated on flexible substrates to broaden their application potential. Among the available options (flexible glass, polymers, and metal foils), polymer-based substrates such as PET and PEN are most widely adopted due to their excellent mechanical flexibility and high optical transparency. Both PET and PEN typically transmit ~ 85% of visible light in the 400–700 nm range [157]. Compared with glass substrates, however, these polymers exhibit stronger absorption in the UV region, with PEN showing more pronounced effect (Fig. 12d). While this UV absorption can partially protect the perovskite layer from degradation, it simultaneously reduces the number of photons available for current generation. To mitigate this trade-off, the incorporation of UV–visible downshifting layers has been proposed as an effective strategy to enhance the overall optical utilization of flexible ST-PSCs [154].

Beyond transparency and efficiency, flexible ST-PSCs must tolerate repeated bending, stretching, and twisting without cracking or interfacial delamination. Most materials and deposition strategies developed for rigid ST-PSCs can be transferred to flexible substrates, although minor process optimization is often required to enhance mechanical durability. A key parameter governing mechanical behavior is Young’s modulus, where lower values correspond to softer and more flexible materials [158]. The Young’s modulus increases from iodide- to bromide- and chloride-based perovskite compositions [159]. This trend may suggest that bandgap widening through partial halide substitution could compromise mechanical durability. However, so far, studies have not shown that wide-band-gap perovskite films suffer more mechanical failure than standard-band-gap perovskites after repeated bending [155, 160, 161]. This means that halide substitution for bandgap tuning can maintain mechanical stability comparable to that of standard perovskites. Nevertheless, perovskite films remain intrinsically brittle, particularly at grain boundaries [162]. To address this limitation, doping strategy has been explored. For example, Xie et al. introduced a polymer dopant (PBDTTPD) that interacts with Pb^2+^ defects at grain boundaries in wide-band-gap perovskite films, leading to reduced efficiency loss under repeated bending (Fig. 12e) [155]. These flexible ST-PSCs were further demonstrated as tandem top cells, achieving an efficiency of 16.2% and an overall tandem PCE of 23.0%.

The commonly used crystalline ITO transparent electrode is relatively brittle, and its conductivity reduces significantly when repeatedly bent at a curvature radius below 14 mm [49]. Better flexibility can be achieved with amorphous electrodes, typically deposited at low temperatures. For instance, amorphous ITO improves the critical bending radius to around 10 mm [163]. However, this amorphous nature often reduces electrical conductivity, limiting the performance of flexible ST-PSCs. To overcome these challenges, alternative materials that combine high flexibility with excellent conductivity are needed. Notably, Lim et al. demonstrated an amorphous IGTO transparent electrode with only a 0.8% PCE reduction compared to opaque-based ST-PSCs. This electrode also maintained its electrical resistance over 10,000 bending cycles, even at a bending radius of 5 mm for both inner and outer bending (Fig. 12f) [156].

### Semitransparent Perovskite Minimodule

Efforts to develop semitransparent perovskite solar minimodules (ST-PSMs) have been actively pursued to advance this technology toward practical implementation. For large-area modules, the spin coating technique commonly employed in laboratory-scale studies is less suitable, as it often produces films with poor thickness uniformity. This limitation arises from the radial centrifugal force during spin coating, which induces pronounced thickness gradients and spatial inhomogeneity across the film [164]. In ST-PSC employing ultrathin absorber layers, such inhomogeneity can lead to incomplete coverage and pinhole formation, leading to direct contact between the ETL and HTL and ultimately device shunting. Besides, approximately 90% of the precursor material is wasted during the spin deposition, which unnecessarily increases the production cost [7]. In contrast, scalable fabrication methods such as slot-die coating, blade coating, inkjet printing, and thermal evaporation have demonstrated the capability to produce high-quality films over large area for both opaque PSCs and ST-PSMs with reasonable efficiency [165, 166]. Previous study shows that blade or slot-die coating uses about ten times less perovskite solution than spin coating when depositing films over a 15 × 15 cm^2^ area [167]. Importantly, both solution-based and vapor-based scalable methods are capable of fabricating all functional layers in ST-PSCs, enabling either fully solution-processed or fully vapor-processed device architectures [33, 64].

**Fig. 13:**
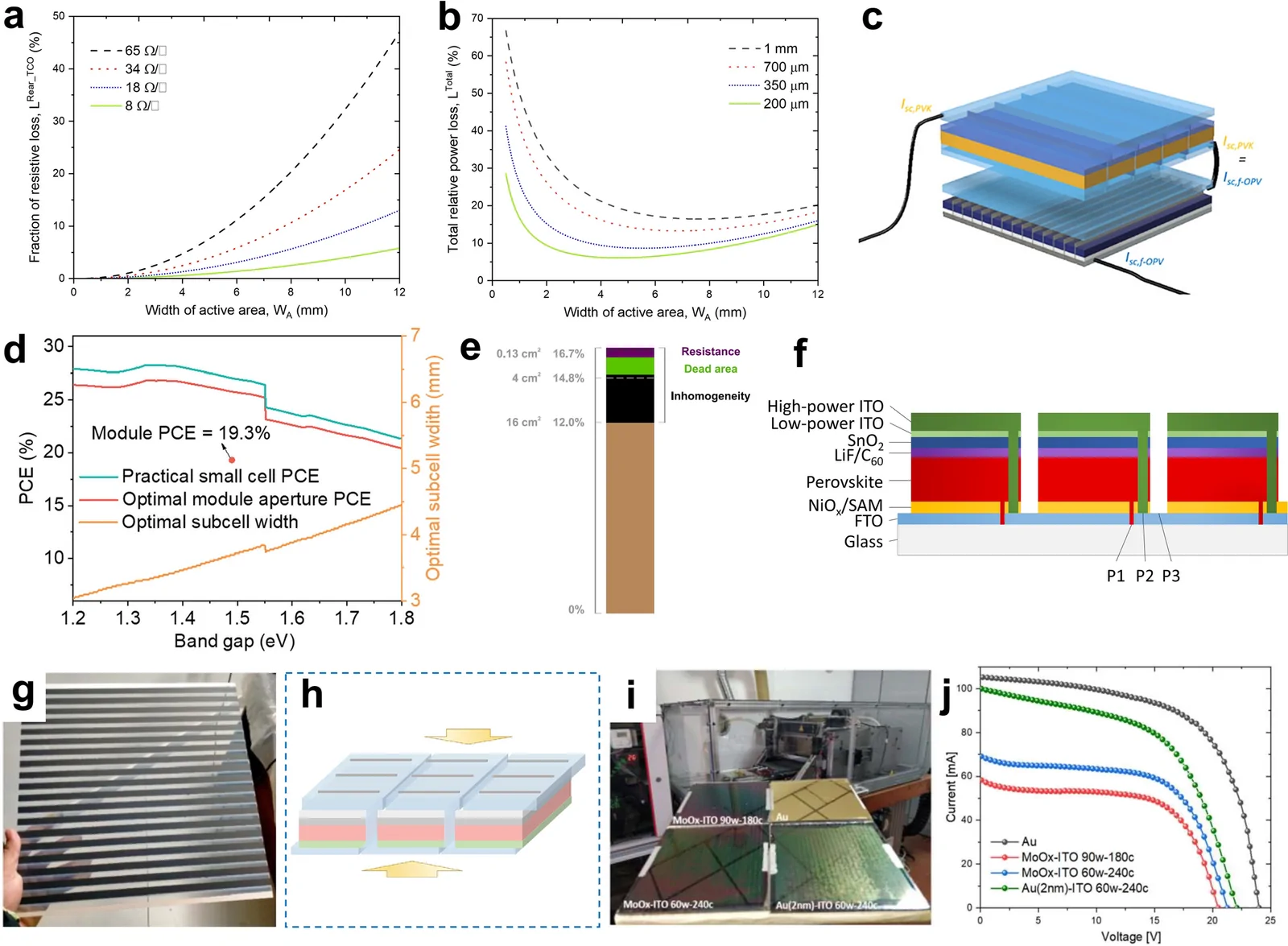
**a** Fraction of resistive loss as a function of subcell width for different rear TCO sheet resistances. **b** Total relative power loss as a function of subcell width for different interconnection widths. Reproduced under terms of the CC-BY-NC license [169]. Copyright 2022, The Authors, published by Wiley. **c** Schematic illustration of 2 T mechanically stacked module. Reproduced under terms of the CC-BY-NC-ND license [169]. Copyright 2023, The Authors, published by Wiley. **d** PCE limit and optimal subcell width of perovskite module as a function of perovskite band gap. Reproduced under terms of the CC-BY license [170]. Copyright 2022, The Authors, published by American Physical Society. **e** Schematic of factors contributing to PCE reduction in ST-PSMs as device area increases. Reproduced with permission [171]. Copyright 2019, Wiley. **f** Schematic illustration of the ST-PSM with wider P2 and P3 scribe line widths. **g** Photograph of ST-PSM with a large scribed area. Reproduced with permission [172]. Copyright 2024, Elsevier. **h** Schematic diagram of the structure of bifacial ST-PSM containing Ag grid lines. Reproduced with permission [54]. Copyright 2023, Springer Nature. **i** Photographs of the ST-PSM along with the employed slot-die coater. **j** Corresponding I–V curves of ST-PSMs employing different buffer layers and top electrodes. Reproduced under terms of the CC-BY license [167]. Copyright 2024, The Authors, published by IEEE

A more pressing issue in ST-PSMs, however, lies in the significant resistive losses associated with transparent electrodes. As discussed earlier, thick transparent electrode on the rear side is required to enhance the film conductivity, but this feature concurrently increases the parasitic absorption [17]. Since conductivity can only be increased to a limited extent, large-area ST-PSMs suffer from high resistive losses, which reduces the FF of the devices. Besides, large-area single cells typically generate very high current densities and low voltages, which complicate module interconnection and increase power losses. To mitigate these losses, monolithic architecture is adopted, in which large-area ST-PSMs are divided into smaller subcells connected in series using P1–P3 laser scribing, similar to CdTe and CIGS modules [168]. Generally, reducing the width of each subcell may lead to lower resistive losses but simultaneously increases the fraction of inactive regions or “dead areas.” Therefore, the subcell width must be optimized to balance the lateral charge transport in the transparent electrodes and the dead-area losses. As shown in Fig. 13a, resistive losses in ST-PSMs increase exponentially with subcell width, especially at higher sheet resistance of rear TCO [169]. Minimum power loss is typically achieved with subcell widths of 4–6 mm and interconnection (dead-area) widths of 200–350 µm (Fig. 13b).

Adjusting subcell widths is also critical to achieve current matching, particularly in 2 T mechanically stacked tandems (Fig. 13c). For example, in a 2 T perovskite (FAPbBr_3_)/organic (PM6:Y6:PCBM) tandem mini-module developed by Cerrillo et al., optimal J_SC_ matching was attained by setting the perovskite and organic subcell widths to 7.22 and 3.19 mm, respectively, yielding a champion tandem efficiency of 14.94% for a 20.25 cm^2^ aperture module [173]. It is worth noting that current density has a major influence on performance losses during upscaling, because resistive and interconnection losses increase with higher current. Consequently, upscaling ST-PSMs based on wider band gap compositions may lead to lower performance losses, as these devices typically provide lower J_SC_ but higher V_OC_. In addition, the width of the subcells can be increased for wider band gap perovskites due to the reduced photocurrent resulting from lower light absorption (Fig. 13d) [170]. This reduces dead area loss and enables more efficient light harvesting.

Apart from electrical resistance and dead area, performance losses during upscaling of ST-PSMs are also caused by film inhomogeneity. However, the relative influence of these three factors depends on the module size. As shown by Jaysankar et al., scaling an ST-PSM from a laboratory-scale cell (0.13 cm^2^) to a small-area module (4 cm^2^) reduces the PCE from 16.7% to 14.8%, with losses originating from all three factors and dead area being the dominant contributor (Fig. 13e) [171]. Further scaling to a larger module (16 cm^2^) using the same device design decreases the PCE to 12.0%, a reduction attributed exclusively to increased film inhomogeneity. This is because both the geometric fill factor and cell stripe width remain unchanged, indicating that dead area and electrical resistance do not introduce additional losses at this stage. This loss distribution is expected to persist in even larger modules, highlighting the critical importance of minimizing dead area, enhancing electrode conductivity, and ensuring uniform film formation to achieve high-efficiency ST-PSMs.

Although scribing removes part of the active area and reduces light absorption, it concurrently increases optical transparency. This effect becomes more pronounced when the widths of the P2 (scribing before rear electrode deposition) and P3 (scribing after rear electrode deposition) lines are widened. Enhanced transparency of ST-PSMs is particularly advantageous for applications that demand higher visibility, such as BIPV and agrivoltaics. Interestingly, Wang et al. demonstrated that a slightly wider P2 scribing not only increases transparency but also enhances contact continuity between the ITO rear electrode and the FTO front electrode (Fig. 13f) [14]. The exposed scribing region allows more ITO to fill the gap, forming a thicker connection layer that reduces series resistance and raises the FF to 78%. In addition, a wider P3 line improves electrical insulation between adjacent sub-cells and minimizes current leakage caused by the slight conductivity of the charge transport layer. When integrated with a silicon module in a 4 T tandem configuration, the improved transparency of the ST-PSM increases the I_SC_ of the silicon bottom cell, despite a minor reduction in the I_SC_ of the perovskite top module itself. Consequently, a 240 cm^2^ ST-PSM achieved a PCE of 16%, while the corresponding 4 T tandem device reached a certified PCE of 25.9%.

To achieve higher AVT for specific applications, it is theoretically possible to substantially increase the P2- and P3-scribed areas in ST-PSMs. As illustrated in Fig. 13g, an ST-PSM prepared on 900 cm^2^ substrate only has ~ 200 cm^2^ active area [172], which closely mimics the spatial segmentation strategy used in the conventional semitransparent modules. However, this design offers little advantage for ST-PSMs, given their much lower efficiency when normalized to the total installed area. Moreover, this design is not cost-effective, as the transparent dead areas are actually TCO-coated glass, which represents the most expensive component of the module [17]. In contrast, micropatterning strategies, as discussed earlier, offer a more practical solution for ST-PSMs, which enables improved color neutrality while maintaining acceptable efficiency.

Another strategy to address the poor conductivity of transparent electrodes is the introduction of metal grid lines, as commonly employed in silicon solar cells. Gu et al. incorporated Ag grid lines on the rear ITO electrode of each subcell while preserving the monolithic architecture (Fig. 13h) [54]. Owing to the high conductivity of Ag, charge carriers from the ITO are collected by the grid lines and rapidly transported to adjacent subcell. The grid geometry, including width and spacing, was systematically optimized to balance the electrical conductivity and optical shading losses. An optimal configuration was achieved using a grid width of 0.2 mm and a spacing of approximately 2 mm. Without the Ag grid, the FF decreased by about 5%. The optimized minimodule, with an aperture area exceeding 20 cm^2^, achieved impressive bifacial PCEs of 20.2% under front illumination and 15.0% under rear illumination.

Nevertheless, most ST-PSMs developed in the laboratory still have limited active areas below 50 cm^2^. Castriotta et al. achieved a significant milestone by fabricating a 225 cm^2^ ST-PSM (Fig. 13i) with a PCE of 13.18%, demonstrating its strong potential for industrial adoption despite the comparatively lower efficiency. The device employed the structure MgF_2_/glass/FTO/SnO_2_/CsMAFAPbIBr/PEAI/spiro-OMeTAD/MoO_*x*_ or Au (2 nm)/ITO [167]. The buffer and transparent electrode layers were deposited by thermal evaporation and sputtering, respectively, while all other functional layers were fabricated via blade or slot-die coating. The study revealed that incorporating an ultrathin Au buffer layer resulted in superior device performance compared to MoO_*x*_ (Fig. 13j), primarily due to the improved *V*_OC_ and *J*_SC_. Such enhancement originated from the better energy-level alignment of Au with the spiro-OMeTAD HTL, enhanced protection of the underlying layers during sputtering, and reduced shunt resistance.

## Key Strategies for Stability Optimization

The stability of PSC has been extensively reviewed in the literature [174–177]. In contrast, the present discussion specifically focuses on stability challenges unique to ST-PSC and the corresponding strategies developed to improve their operational durability. Notably, many of these strategies address multiple degradation pathways simultaneously, reflecting the interdependent nature of stability issues in ST-PSCs.

### Phase Segregation in Perovskite

**Fig. 14:**
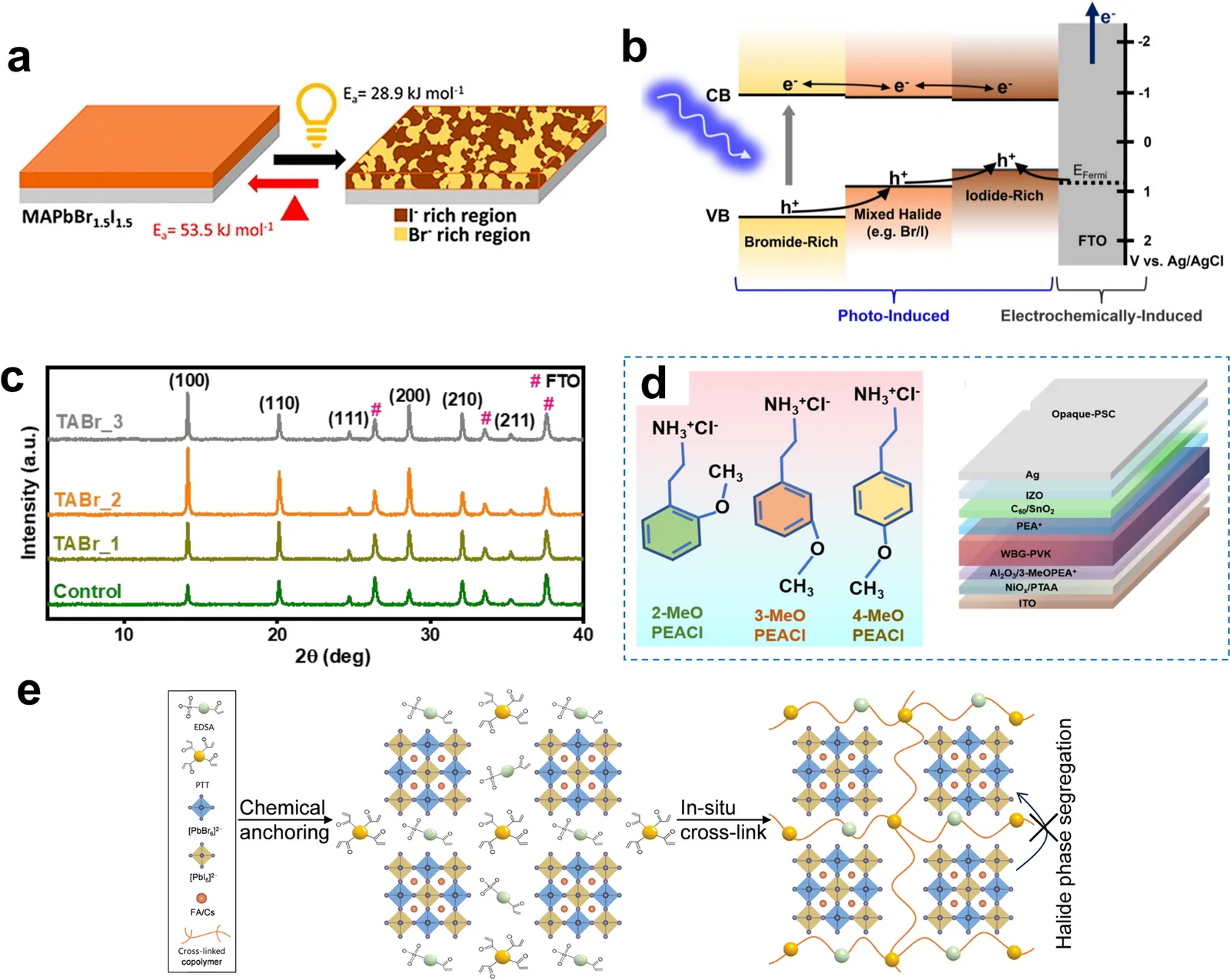
**a** Schematic illustration of light-induced phase segregation and subsequent dark-state remixing in mixed-halide perovskite films. **b** Band diagram illustrating carrier trapping mechanism in mixed-halide perovskite after phase segregation. Reproduced with permission [182]. Copyright 2022, American Chemical Society. **c** XRD spectra of perovskite films without and with TABr additives. Reproduced with permission [39]. Copyright 2024, American Chemical Society. Chemical structures of 2-, 3-, and 4-MeOPEACl. **d** Chemical structure x-MeOPEA⁺ ligands and schematic diagram of the corresponding solar cell incorporating the ligands at the buried perovskite/HTL interface. Reproduced with permission [40]. Copyright 2024, American Chemical Society. **e** Schematic diagram of copoly-PE self-assembles at perovskite grain boundaries via chemical anchoring to form a percolating network and prevent phase segregation. Reproduced with permission [41]. Copyright 2025, Royal Society of Chemistry

Since semitransparency is commonly achieved by widening the band gap, the influence of mixed-halide anions on the device stability becomes critical. Generally, incorporating a small fraction of Br⁻ into the perovskite lattice can improve the structural stability, as the smaller ionic size of Br⁻ and the stronger Pb–Br interaction compared to Pb–I can create a more robust crystal framework [178]. Nonetheless, perovskites with high Br content are prone to phase segregation under illumination, thermal stress, or electric fields as a result of ion migration [179, 180]. In particular, ion migration preferentially occurs at grain boundaries, where vacancy concentrations are high, because halide ions and vacancies have low migration activation energies [181]. However, I⁻ and Br⁻ ions exhibit different activation energies, which causes them to respond differently under the same driving forces and eventually generates I-rich and Br-rich domains (Fig. 14a) [180]. Due to the band offset between these I/Br phases, the I-rich domains trap the photogenerated carriers and act as recombination centers (Fig. 14b) [182]. This phenomenon, commonly known as the Hoke effect, has been observed in MAPb(Br_*x*_I_1-*x*_)_3_ for 0.2 < *x* < 1 [183], in FAPb(Br_*x*_I_1-*x*_)_3_ for 0.55 < *x* < 0.9 [184], and in CsPb(Br_*x*_I_1-*x*_)_3_ for 0.4 < *x* < 1 [185], which typically corresponds to band gaps above 1.7 eV. Although phase segregation is reversible in the dark because entropy favors a homogeneous halide distribution [186], its occurrence can accelerate V_OC_ and PCE decay through intensified non-radiative recombination [110].

To mitigate this issue, numerous strategies have been explored including composition control, additive engineering, and surface passivation. In composition engineering, controlling the A-site cations has proven effective in improving the perovskite stability by increasing the kinetic barrier for ion migration. As reported by Yu et al., CsFA-based perovskites exhibit superior stability compared to MA- and CsFAMA-based analogs [38]. Moreover, the morphology of CsFA-based perovskites remained largely unaffected by variations in Br concentration, enabling ST-PSCs to reach PCEs of 15.5% at 20.7% AVT. Another effective strategy involves incorporating additives into the perovskite precursor solution. As demonstrated by Garai et al., the use of tryptamine hydrobromide (TABr) as additive not only passivated halide and metal ion vacancies, but also regulated crystallization to enhance the perovskite crystallinity (Fig. 14c) [39]. This allowed the ST-PSCs with 22% AVT to achieve a PCE of 14.21%.

A further method relies on interfacial engineering, as shown by Zhang et al., who employed methoxy-substituted phenylethylammonium (*x*-MeOPEA⁺) ligands (specifically 2-, 3-, and 4-MeOPEACl) at the perovskite/HTL buried interface (Fig. 14d) [40]. Here, the methoxy groups chemically bond with mesoporous Al_2_O_3_ and the ammonium groups passivate the perovskite interfacial defects. Besides, the presence of these ligands promoted the growth of vertically oriented crystals and homogenized the I-Br distribution in the wide-band-gap perovskites, thereby suppressing phase segregation. As a result, ST-PSCs delivered a PCE of 18.5% (15.4% for the IZO front side). Cross-linked network polymers are another class of rising materials that capable of suppressing phase segregation by passivating grain boundary defects and reducing strain, thereby inhibiting ion migration. Their percolating network could provide superior stabilization compared with small molecules and linear polymers. A notable example of cross-linked polymer is formed from pentaerythritol tetraacrylate and 3-[[2-(methacryloyloxy)-ethyl]dimethylammonio]propane-1-sulfonate (copoly-PE) (Fig. 14e), which enabled ST-PSCs with a *V*_OC_ deficit of only 0.40 V to retain 95% of their efficiency after 550 h [41].

### Ion Diffusion in Other Components

**Fig. 15:**
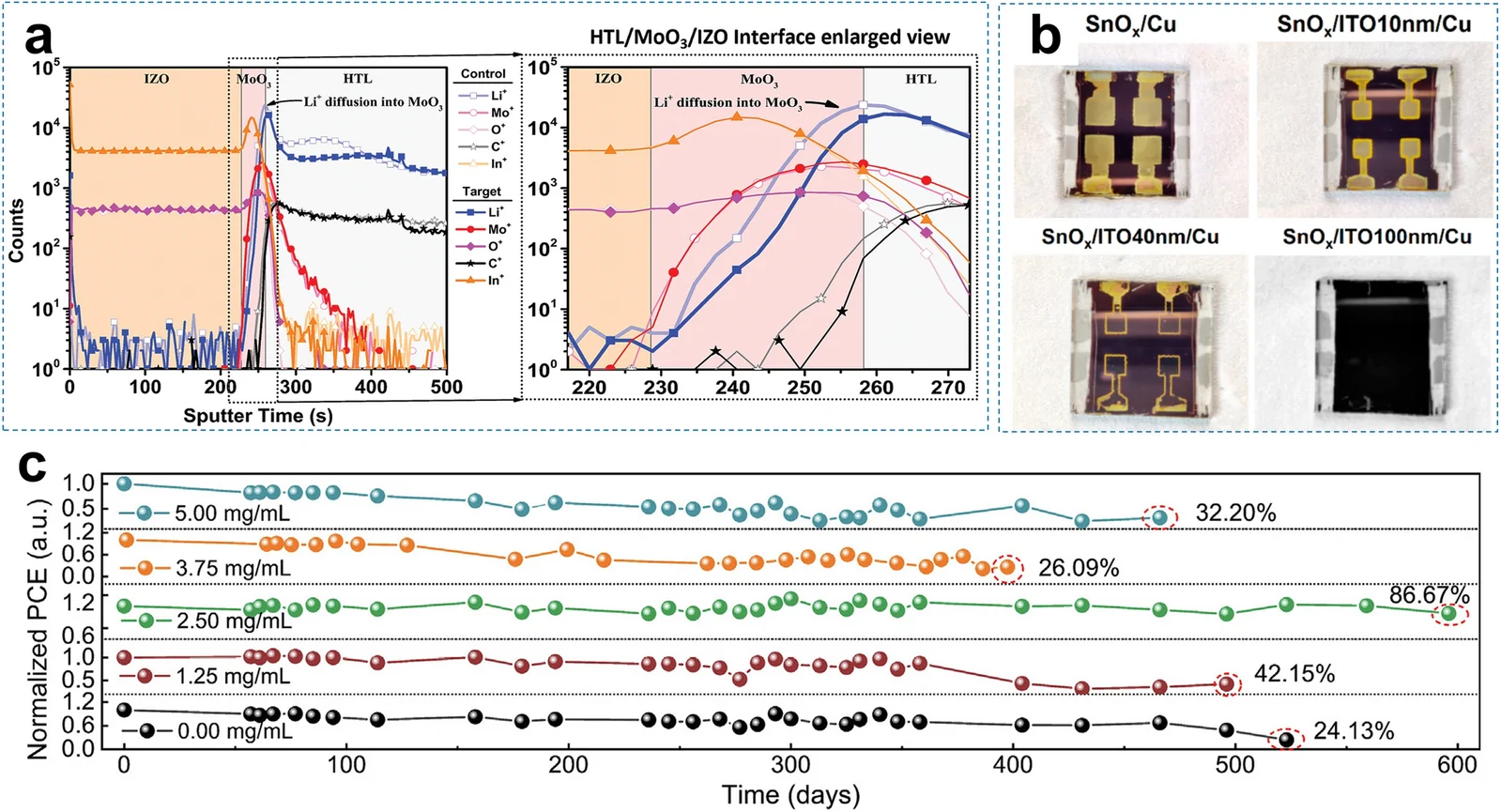
**a** Wide and enlarged views of TOF–SIMS depth profiles showing Li^+^ diffusion across the HTL/MoO_3_/IZO interface in ST-PSCs oxidized for 3 h (control) and 4 days (target). Reproduced under terms of the CC-BY-NC license [51]. Copyright 2023, The Authors, published by Wiley. **b** Photographs of devices incorporating ITO layers of different thicknesses beneath Cu electrode. Reproduced under terms of the CC-BY license [71]. Copyright 2022, The Authors, published by American Chemical Society. **c** Long-term stability of ST-PSCs without and with different amount of PANI additive in spiro-OMeTAD. Reproduced with permission [72]. Copyright 2024, Elsevier

In addition to ion migration within the perovskite, ions from other components can also diffuse within the device, leading to detrimental reactions. In PSC fabrication, the spiro-OMeTAD layer is typically exposed to dry air for several hours to promote its oxidation and enhance conductivity, which can increase the FF to values beyond 80% [187]. However, insufficient oxidation of spiro-OMeTAD can be detrimental in ST-PSCs, as residual Li^+^ ions from the Li-TFSI dopant tend to dissociate and diffuse into the MoO_3_ buffer layer (Fig. 15a). This diffusion progressively decreases the work function of MoO_3_ and increases the hole-injection barrier at the HTL/MoO_3_ interface, thereby accelerating performance degradation. In contrast, extending the oxidation duration enables complete conversion of the reactive Li^+^ species at the HTL surface into more stable lithium oxides (Li_2_O and Li_2_O_2_), which effectively suppresses Li-ion diffusion. This behavior ultimately enhances both the initial PCE and long-term stability of ST-PSCs [51].

Metal-based contacts, such as ultrathin metal or DMD electrodes, may easily diffuse into the perovskite film in the presence of electric field and readily react with halide species to form metal halides, a process that becomes more pronounced at elevated temperatures [175, 188]. This interaction generates shunting pathways and results in irreversible device degradation [189]. However, the diffusion behavior of metal electrodes in conventional opaque devices differs from that in ST-PSC due to the presence of the bottom dielectric layer. For example, in opaque devices without buffer layer, the incorporation of a thin Cu layer beneath a thick Ag layer is beneficial to minimize diffusion and suppress the formation of AgI [190]. In contrast, Cu readily diffuses into MoO_3_, and introducing an ultrathin Ag layer at the MoO_3_–Cu interface can prevent this diffusion [191]. Therefore, Ag is generally more preferable than Cu in DMD electrodes in order to maintain good device stability [70]. Metal diffusion can also be prevented by the ITO rear electrode with sufficient thickness and this is particularly important for the semitransparent module, where metal grid might be required. It was shown that ITO with at least 100 nm thickness is required to prevent perovskite degradation due to Cu diffusion (Fig. 15b). Devices with Cu-only rear electrodes can reach higher PCE (18.8%) but lower stability (T_80_ < 50 h), whereas ITO/Cu devices achieved much higher stability (T_80_ > 400 h) despite the slightly lower PCE (17.8%) [71]. Although this result was obtained from opaque devices, the role of ITO in suppressing metal diffusion is highly relevant to the design and stability of ST-PSCs.

Modification of the HTL is also crucial to ensure that the ST-PSC retains high performance over an extended period, particularly when the transparent top contact involves a thin metal-based electrode. Chen et al. demonstrated that incorporating 2.5 mg mL^−1^ polyaniline (PANI) into spiro-OMeTAD significantly improved the stability of ST-PSCs, as PANI suppressed the diffusion of Au atoms from the electrode into the perovskite layer [72]. Moreover, PANI interacted with the Li-TFSI and tBP additives to inhibit water ingress. It also mitigated the corrosive effect of tBP on the perovskite layer, promoted the oxidation of spiro-OMeTAD, and chemically passivated defects by coordinating with Pb^2+^ ions and filling I^−^ vacancies. Owing to these multifunctional effects, the ST-PSC achieved a PCE of 15.66% (17.26% under bifacial operation) and retained approximately 87% of their initial performance for over 596 days under 50%–90% relative humidity even without encapsulation (Fig. 15c).

### Sputter-Induced Damage

**Fig. 16:**
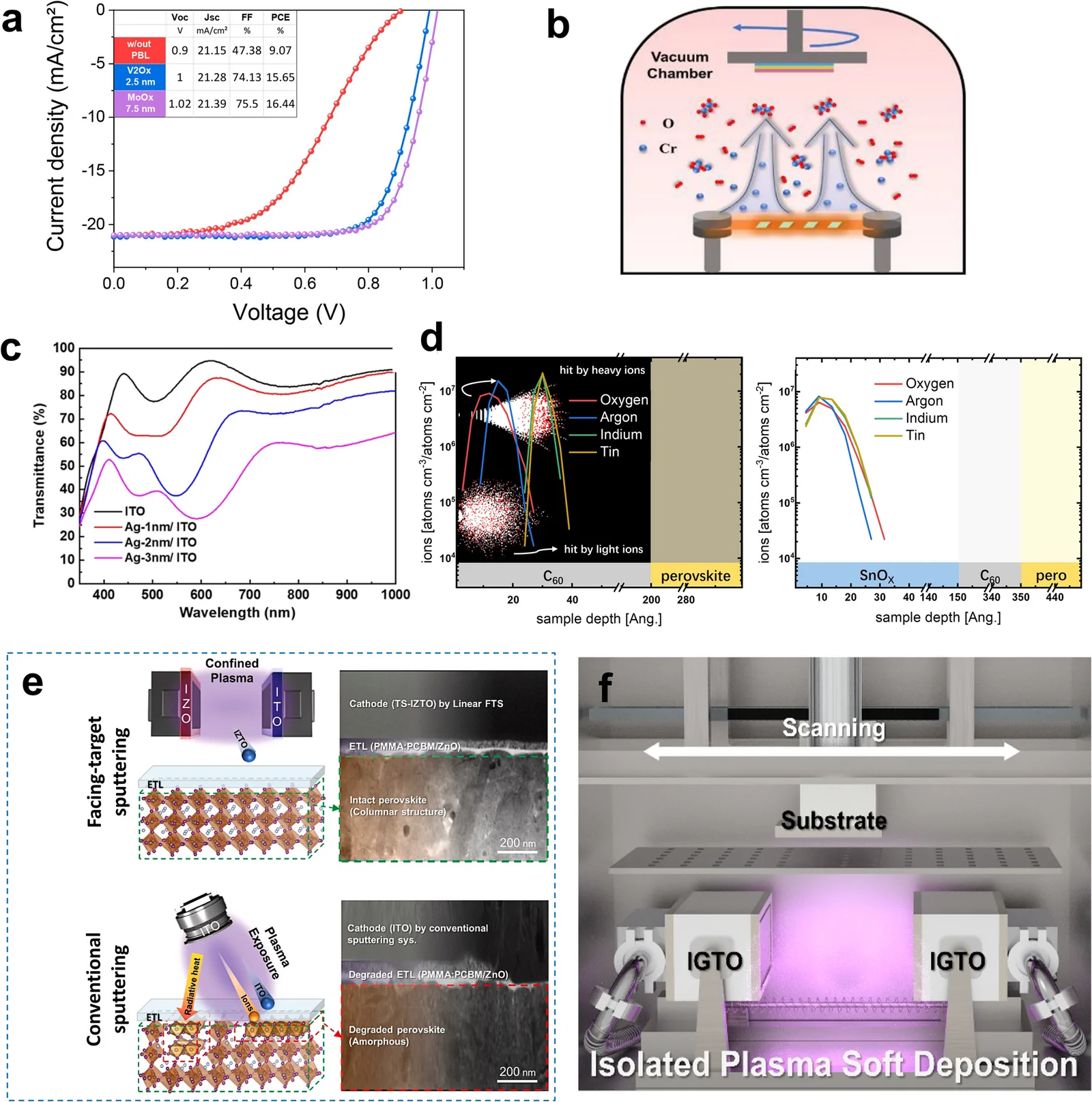
**a** J–V characteristics of ST-PSCs without a buffer layer and with V_2_O_x_ or MoO_x_ buffer layers. Reproduced under terms of the CC-BY license [59]. Copyright 2023, The Authors, published by American Chemical Society. **b** Schematic diagram of CrO_x_ film fabrication using to-RTD process. Reproduced with permission [194]. Copyright 2023, Elsevier. **c** Transmittance spectra of glass/Ag/ITO electrodes with varying Ag buffer layer thicknesses. Reproduced with permission [84]. Copyright 2023, American Chemical Society. **d** Ion penetration depth profiles during ITO sputtering for perovskite/C_60_ and perovskite/C_60_/SnO_x_ stacks. Reproduced under terms of the CC-BY license [58]. Copyright 2024, The Authors, published by Royal Society of Chemistry. **e** Schematic illustrations of (top) the facing-target sputtering configuration and (bottom) the conventional sputtering system used for depositing IZTO or ITO transparent electrodes. The right panels show cross-sectional HR-TEM images of the corresponding ST-PSCs. Reproduced with permission [60]. Copyright 2020, Elsevier. **f** Schematic illustration of the isolated plasma soft deposition system used for depositing IGTO films. Reproduced with permission [61]. Copyright 2025, Elsevier

The sputtering technique used for depositing ITO is an industry-compatible process that employs plasma excitation and energetic ion bombardment to eject atoms from a target material, enabling their uniform deposition onto the substrate [192, 193]. However, high-energy particle bombardment can damage the organic HTL and perovskite layers and, in severe cases, cause delamination of the underlying layers [84]. The sputtering process can also lead to dissociation of C = N bonds at the perovskite surface, resulting in the loss of organic moiety [58]. Such damage increases the series resistance and energy barrier at the charge transport layer/ITO interface, which interrupts the carrier transport and causes an “S-shaped” J–V curve (Fig. 16a) [59]. To mitigate this, a thin buffer layer (typically based on metal oxide or ultrathin metal) with low parasitic absorption and suitable band alignment is deposited by thermal evaporation prior to sputtering. The optimal buffer layer thickness typically ranges from 2 to 15 nm, depending on the material and fabrication parameters used. Several materials have been developed as buffer layers, and each of them offers distinct advantages and limitations.

MoO_3_, the most widely used buffer for conventional ST-PSCs, provides high conductivity for efficient hole extraction, but oxygen vacancies in MoO_3_ may trigger decomposition reaction toward iodide ions on perovskite surface [195]. V_2_O_*x*_ exhibits better stability under light soaking and suppresses oxidation and Li-ion diffusion in the HTL, though its efficiency is slightly lower than that of MoO_3_ [59]. SnO_2_, commonly employed in inverted ST-PSCs, offers excellent charge transport and stability but is typically deposited by atomic layer deposition (ALD), which is costly and less practical for large-scale production [196]. CrO_*x*_ has also shown promise as a buffer layer, enabling ST-PSCs with 19% PCE using only a 4 nm film, but its deposition by trace-oxygen reactive thermal deposition (to-RTD) requires precise process control (Fig. 16b) [194]. For metal-based buffer layers such as Ag, overly thin films provide inadequate protection against sputtering damage, whereas just slightly thicker layers can significantly reduce the AVT due to the increased reflection and plasmonic absorption (Fig. 16c) [84].

Previously, plasma radiation during sputtering was believed to be a major cause of damage to perovskite and organic HTLs [197]. However, Yang et al. reported that sputtering-induced damage originates primarily from ion bombardment rather than plasma radiation [58]. In fact, ST-PSCs exposed to plasma radiation showed over 1% higher efficiency than unexposed samples, as plasma radiation can relieve lattice strain and suppress nonradiative recombination. In contrast, bombardment by ions such as O, Ar, Sn, and In can penetrate up to ~ 4 nm into the exposed film (Fig. 16d). In the C_60_ layer, ion bombardment induces defect formation, while in the SnO_2_ layer, it does not significantly damage the material but triggers phonon propagation that transfers heat deeper into the film, leading to C=N bond breakage at the perovskite surface. Increasing the SnO_2_ thickness to 15 nm effectively scatters phonons and shields the perovskite from heat-induced damage during ITO sputtering, allowing the ST-PSC to achieve a PCE comparable to that of its opaque counterpart.

Increasing the sputtering power during ITO deposition improves film conductivity, but this process is not recommended due to the presence of damaging energetic particles. To overcome this issue without using the buffer layer, low-power or “soft” sputtering approach has been explored [198]. Although this approach effectively protects the underlying layers, it often results in ITO films with higher sheet resistance and poorer electrical conductivity [58]. Currently, there is no universal guideline for the optimal deposition parameters (such as power density, working pressure, or substrate-to-target distance) to achieve damage-free ITO sputtering, as these conditions may vary greatly depending on the equipment design and configuration. An alternative and more robust strategy involves employing a bilayer ITO structure, where a thin ITO layer is first deposited at low power to minimize damage, followed by a high-power deposition of thick ITO to restore conductivity. This approach has enabled the fabrication of ST-PSCs with a high FF approaching 80% [14].

Sputtering-induced damage can also be mitigated by modifying the sputtering system design. Lim et al. introduced a facing-target sputtering configuration to prepare InZnSnO (IZTO) transparent electrodes, in which two parallel targets face each other while the substrate is positioned farther from the plasma region (Fig. 16e) [60]. This setup generates strong magnetic fields and a negative potential between the targets, which confines the high-density plasma and prevents direct irradiation on the sample. As a result, the morphology of all layers is well preserved and the device efficiency significantly improved from 3.43% to 15.72% compared with the conventional direct sputtering system. However, this approach suffers from poor uniformity due to uneven plasma distribution and a low deposition rate. Later, the isolated plasma soft deposition method was developed, where a metal grid and linear slit were employed in the sputtering chamber to separate the plasma region from the substrate (Fig. 16f). In this configuration, energetic particles lose energy through collisions within the isolated region before reaching the substrate, enabling uniform large-area deposition. Using this technique, ST-PSCs with ITO and InGaTiO (IGTO) electrodes achieved PCEs of 15.52% and 18.71%, respectively [61, 62]. Although alternative vapor deposition methods such as ALD, reactive plasma deposition (RPD), and pulsed laser deposition (PLD) have been explored to deposit transparent electrodes more gently without buffer layers, sputtering remains the dominant approach due to its industrial maturity and scalability [61, 199].

### Extrinsic Factors

**Fig. 17:**
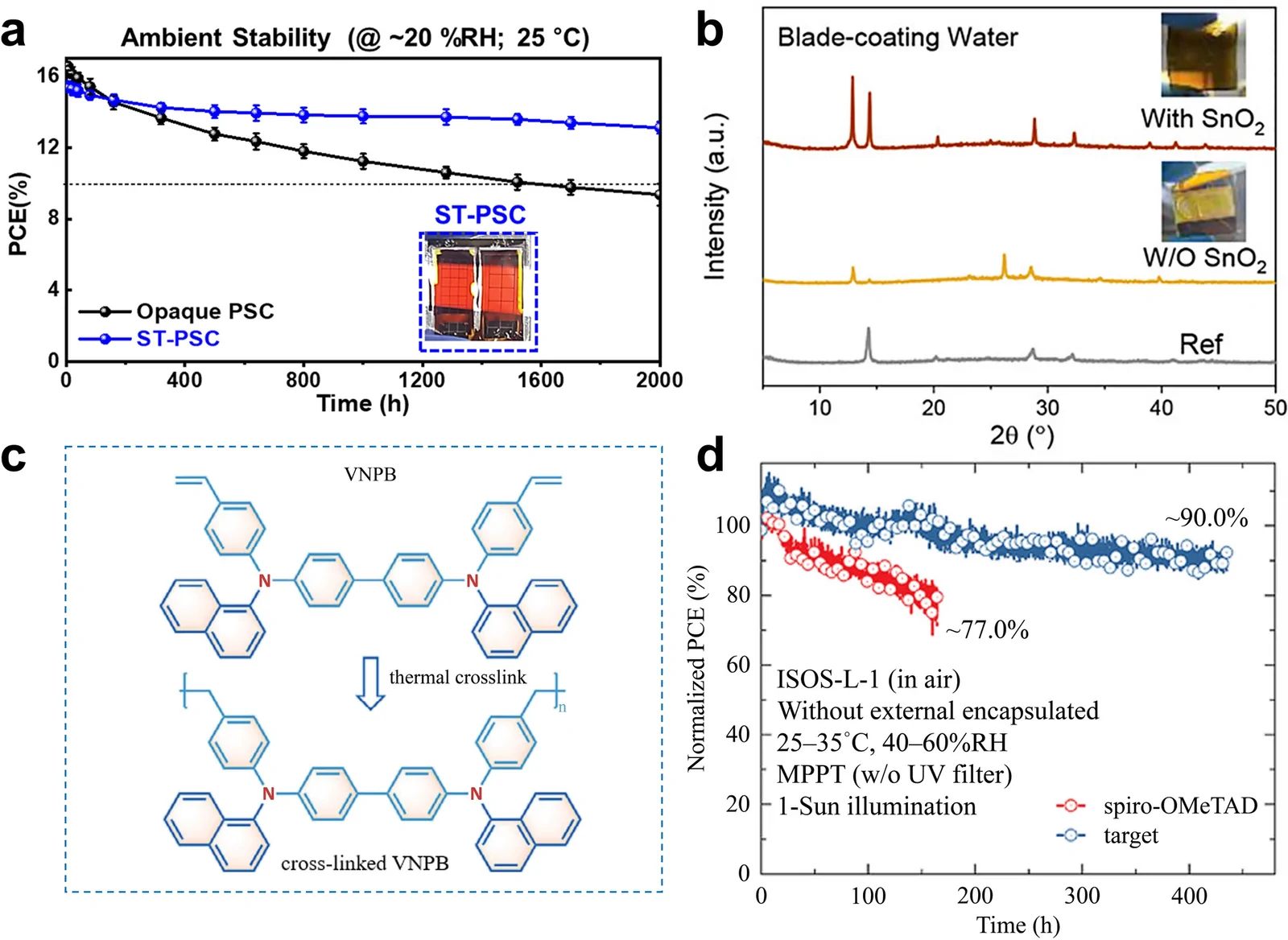
**a** Ambient stability of opaque PSCs and ST-PSCs during air aging. Reproduced with permission [63]. Copyright 2022, Elsevier. **b** XRD spectra and photographs (inset) of perovskite layers with and without SnO_2_ buffer layer after directly exposed to water. Reproduced under terms of the CC-BY license [64]. Copyright 2024, The Authors, published by Wiley. **c** Chemical structure of cross-linked VNPB and its formation via thermal crosslink process. **d** Operational stability test of ST-PSC based on spiro-OMeTAD and cross-linked VNPB. Reproduced with permission [52]. Copyright 2025, Wiley

Similar to opaque PSC, several extrinsic factors, such as humidity, oxygen, UV light, and heat, are well known for the device degradation, although their influence in ST-PSCs may differ slightly [7, 200]. In ST-PSCs, ITO rear electrode can suppress moisture and oxygen ingress more effectively than metal-based electrodes, albeit with slightly lower initial PCE. Superior ambient stability for ST-PSCs was observed by Tyagi et al., where the device retained over 85% of PCE after 2000 h, compared with ~ 60% for opaque PSCs (Fig. 17a) [63]. However, moisture and oxygen ingress are still possible through thinner layer, pinholes, or device edges. In addition, perovskite degradation by-products can react with the ITO, further accelerating device degradation [201]. Therefore, dense and compact ITO films are required to effectively inhibit moisture and oxygen ingress. Notably, such films can even protect the perovskite layer from direct contact with perovskite-dissolving solvents, as demonstrated by the successful fabrication of all-perovskite tandem devices [202]. Additional protection can also be achieved using compact buffer layers, such as MoO_3_ or SnO_2_. For example, a compact 20-nm-thick SnO_2_ layer grown using ALD preserves the perovskite structure even under direct water exposure (Fig. 17b), thereby enabling the processing of transparent electrodes using scalable solution-based techniques, such as blade coating of AgNWs [64].

UV light-induced degradation pathways in ST-PSCs also differ slightly from that in opaque PSCs. In n–i–p architectures, UV irradiation can activate wide-bandgap metal oxide ETLs such as TiO_2_, generating photocatalytic sites that accelerate interfacial perovskite degradation [91, 116]. This effect can be mitigated by using SnO_2_-based ETLs, while the perovskite layer itself can absorb part of the incident UV light [176]. However, in ST-PSCs, high-energy UV photons can also directly impinge from the rear side, which compromises stability if the rear charge transport layer is UV unstable, as reported for spiro-OMeTAD [52]. Substituting the HTL with more UV-stable materials is therefore critical. For instance, VNPB, a cross-linkable p-type small molecule, can enhance UV and thermal stability and suppress perovskite–MoO_3_ reactions (Fig. 17c). Due to its high optical transparency, illumination from rear side gives higher efficiency than front side with a bifaciality of 101.4%. Under maximum power point tracking (ISOS-L-1) conditions, VNPB-based devices retained ~ 90% of their initial PCE after 430 h, whereas spiro-OMeTAD-based devices retained only 77% after 160 h (Fig. 17d) [52]. Even in p-i-n ST-PSC architectures, carbazole-based SAMs (2PACz, Me-4PACz, and MeO-2PACz) can undergo UV-induced decomposition, although they show better light-soaking stability than spiro-OMeTAD. Polymerizing the SAM (e.g., poly-2PACz) significantly improves the UV stability of HTL, but their application in ST-PSCs remains underexplored [203].

Heat-induced degradation represents another critical stability challenge for ST-PSCs. Owing to the low thermal conductivity of perovskite materials, heat can readily accumulate within the device under operation. This heat buildup not only promotes thermal decomposition of the perovskite absorber and organic charge transport layers [204], but also lowers the activation energy for ion migration, thereby accelerating phase segregation [205]. Under hot outdoor conditions, operating temperatures exceeding 60 °C have been reported for ST-PSCs [82]. Several effective strategies have been demonstrated to improve thermal stability, including the incorporation of dopants such as alkali metals [82], polymers [83], and small molecules [52], as well as the replacement of conventional HTL with more thermally robust alternatives [53].

### Encapsulation

The encapsulation process usually involves cover glass and adhesive encapsulant and plays a vital role in protecting perovskite devices from humidity and mechanical damage. Glass itself is an excellent and cost-effective encapsulation component for blocking water and oxygen. Since ST-PSCs are fabricated on glass substrates, applying a cover glass with proper edge sealing not only provides full protection of the device components from the external environment but also maintains the optical transparency. Encapsulation using tempered glass is a widely adopted technology in silicon PV [206], and therefore, it is seen as an excellent option for ST-PSC. This is because it has high transparency with about 90% transmittance for wavelength between 400 and 1100 nm. The transmittance of the glass can reach up to 98% if coated with anti-reflective coating [207]. In addition, the glass can provide excellent mechanical strength and proven long-term durability under harsh outdoor conditions.

**Fig. 18:**
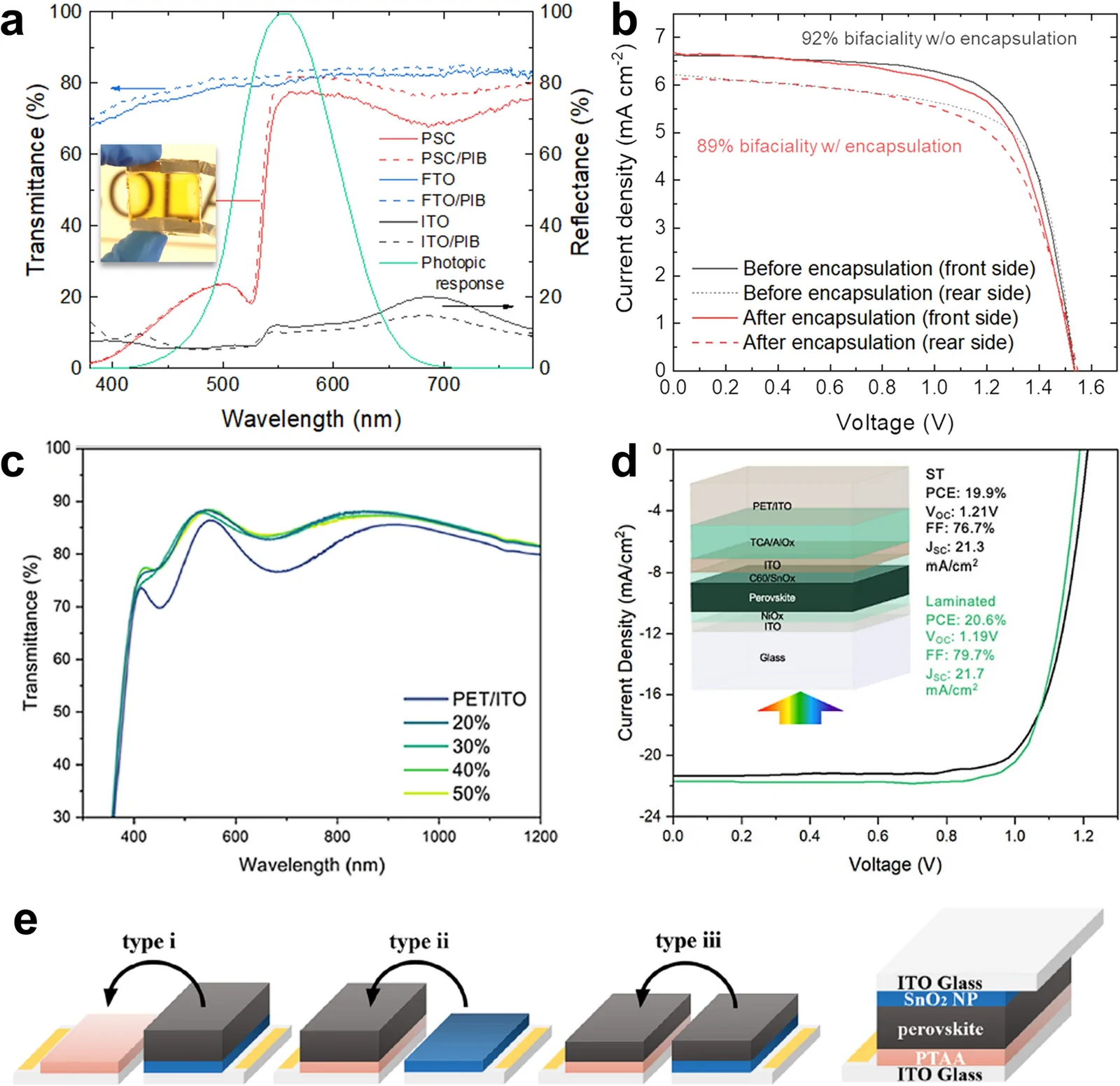
**a** UV–Vis transmittance and reflectance spectra of ST-PSC, FTO, and ITO before and after encapsulation with PIB. Inset is the photograph of the ST-PSC. **b** J–V curves of the ST-PSC measured under front- and rear-side illumination before and after encapsulation with PIB. Reproduced under terms of the CC-BY license [85]. Copyright 2024, The Authors, published by Springer Nature. **c** Transmittance spectra of bare and TCA coated PET/ITO. 20% to 50% indicate the wt% of ionic liquid in the TCA material. **d** *J* − *V* scans of ST-PSC without and with TCA lamination. Reproduced with permission [86]. Copyright 2024, American Chemical Society. **e** Schematic diagram of the fabrication processes of the self-encapsulated ST-PSCs and the obtained device structure. Reproduced with permission [87]. Copyright 2023, American Chemical Society

Meanwhile, a series of adhesive encapsulants have been previously developed for opaque-based PV cells and modules such as EVA, polyolefins (POEs), thermoplastic polyurethanes (TPU), UV curable epoxies (E131 and E132 resin by Ossila, Vitralit epoxy glue by Panacol, etc.), and ionomers (Surlyn, Bylen and Jurasol) [85, 208, 209]. All these encapsulants can be applied to ST-PSCs, but their impact on optical transparency requires further investigation. Mariani et al. developed an encapsulation method based on a transparent viscoelastic semi-solid/highly viscous liquid polyolefin, i.e., low-molecular-weight polyisobutylene (PIB) [85]. By introducing two-dimensional hexagonal boron nitride nanoflakes into PIB, they achieved notable improvements in adhesion, barrier performance, and thermal management. Devices encapsulated with this composite retained more than 80% of their initial efficiency even after rigorous accelerated aging tests. More importantly, after the ST-PSC was encapsulated with the PIB, the AVT of the ST-PSC increased from 58.1% to 62.7% (Fig. 18a). This improvement is attributed to better matching of the refractive indices at the interfaces after encapsulation, allowing PIB to act as an antireflective coating. The J–V curves in Fig. 18b further indicate that the PCE under both front- and rear-side illumination remained essentially unchanged after encapsulation. The bifaciality factor also remained high, decreasing only slightly from 92% to 89% with encapsulation.

Alvianto et al. developed a novel transparent conductive adhesive (TCA) based on a mixture of a transparent polymer matrix poly(vinylidene fluoride-*co*-hexafluoropropylene) (PVDF-HFP), ionic liquid 1-ethyl-3-methylimidazolium bis(trifluoromethylsulfonyl) imide (EMITFSI), and alumina nanoparticles [86]. The TCA layer demonstrates excellent encapsulation capability, ensuring strong environmental stability of the device. Moreover, its refractive index matching with the substrate imparts superior antireflective properties compared to bare PET/ITO, resulting in improved optical transmittance (Fig. 18c). Owing to its high electrical conductivity, the TCA further enhances the *J*_SC_ and FF of ST-PSCs (Fig. 18d). Interestingly, it can also be used to bond two different subcells, such as perovskite and CIGS, enabling operation in a 2T tandem configuration even though both subcells are fabricated separately, as in 4T tandem devices.

Another approach for encapsulating ST-PSCs is to fabricate the functional layers on two separate transparent conductive substrates and subsequently laminate them by compressing them to each other. As shown in Fig. 18e, the perovskite layer can be prepared on either ETL or HTL or both followed by combining them into one cell using the thermocompression bonding process [87]. The obtained device using this method is generally referred to as a self-encapsulated ST-PSC [87, 210]. This technique bears a strong resemblance to the fabrication method of laboratory-scale dye-sensitized solar cells (DSSCs), where the cells are sandwiched using two FTO substrates [211]. In comparison with the conventional encapsulation procedure, this method not only provides effective protection against moisture and oxygen but also simplifies the device fabrication process. Specifically, it integrates the deposition of the transparent electrode with the encapsulation step while enabling the concurrent preparation of half-cells prior to lamination, thereby reducing the processing time. Moreover, this strategy broadens the design flexibility of ST-PSC architectures by eliminating the need for strict process compatibility with the perovskite layer. In other words, ETL and HTL materials or fabrication parameters (e.g., the use of polar solvents or high-temperature annealing) that would normally degrade the perovskite can be employed without concern. Interestingly, the lamination strategy is applicable to both rigid and flexible ST-PSCs, as well as two-terminal tandem devices [212].

Using this approach, Jung et al. achieved a PCE of 17.24% for ST-PSCs based on a 1.67 eV bandgap perovskite, while maintaining over 95% of the initial efficiency during MPPT testing for more than 600 h [87]. During the thermocompression process, crystal growth occurred, leading to enlarged perovskite grain sizes. The process also induced compressive strain in the perovskite layer, which suppressed iodine extraction and phase segregation. Similarly, Cheng et al. optimized the halide concentration gradient within the perovskite layer to enable effective lamination of the top and bottom perovskite. This strategy produced ST-PSCs with PCE approaching 19% and enhanced thermal stability, as the devices retained their initial efficiency for more than 1200 h at 85 °C [88].

Despite the notable progress, achieving high-performance ST-PSCs with high reproducibility via the lamination process remains challenging, mainly due to imperfect interfacial contact between the laminated layers. Voids and trapped air often form during thermocompression, significantly reducing device performance and accelerating internal degradation. Moreover, this approach limits the module design to a single large-area cell rather than multiple monolithically interconnected sub-cells. As a result, the limited conductivity of the TCO across large areas increases series resistance and leads to greater power losses. Nevertheless, with further optimization of module architecture and the development of highly conductive transparent layers, thermocompression strategy could become a viable route for high-performance ST-PSC modules in the future.

## Potential Applications

**Fig. 19:**
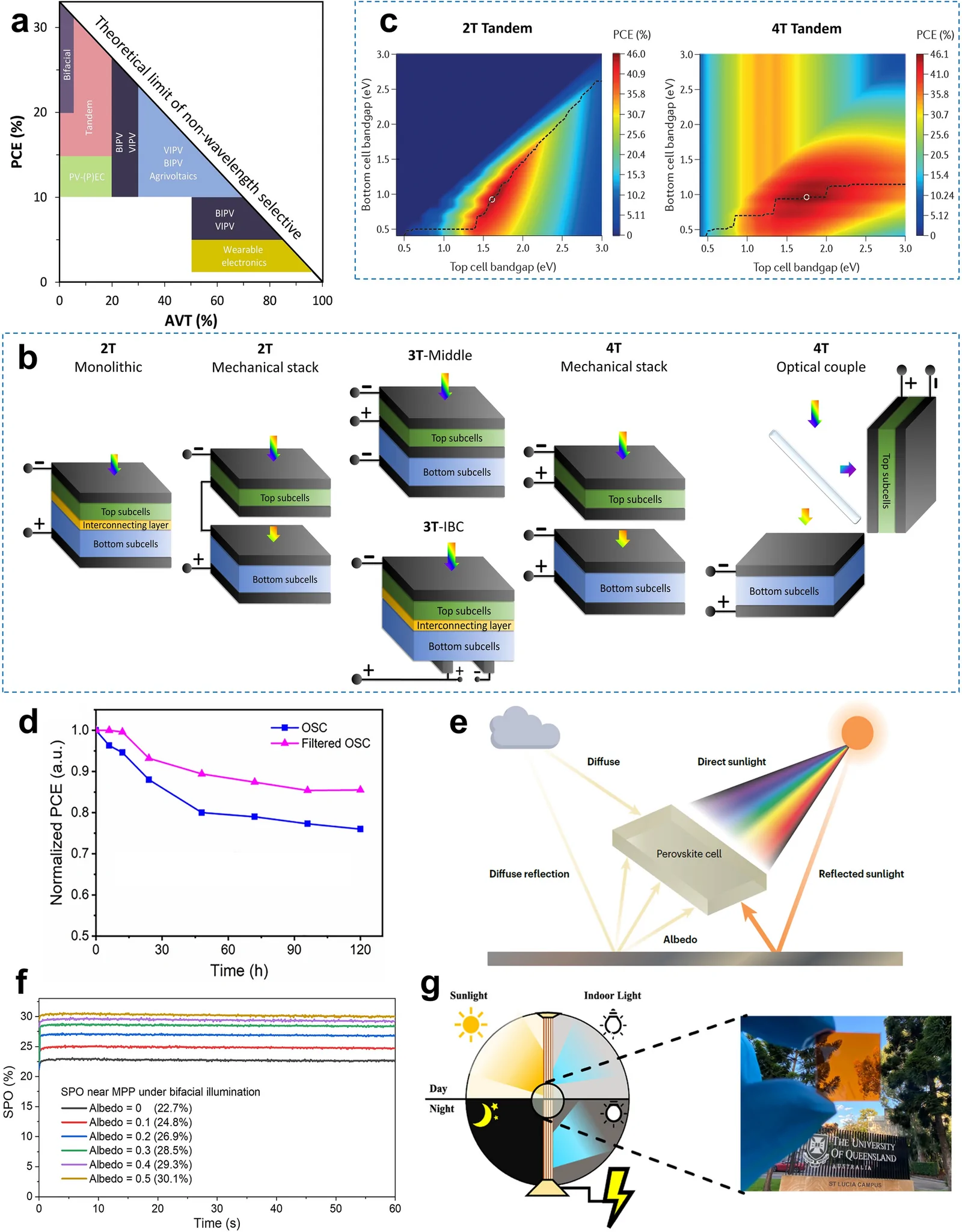
**a** PCE and AVT requirements for ST-PSCs across different application scenarios. **b** Tandem solar cells configuration. **c** Maximum PCE attainable by 2 T and 4 T tandem solar cells with different band gap combination. Reproduced with permission [213]. Copyright 2017, Springer Nature. **d** Stability of polymer solar cells without and with CsPbI_2_Br-based ST-PSC filter under continuous 1-sun illumination. Reproduced under terms of the CC-BY license [217]. Copyright 2022, The Authors, published by Springer Nature. **e** Schematic illustration of light absorption by a bifacial ST-PSC. Reproduced with permission [117]. Copyright 2024, Springer Nature. **f** J–V characteristics of a bifacial PSC measured under simultaneous front and rear illumination, with 1 Sun (100 mW cm^−2^) incident from the front and 0–0.5 Sun albedo (0–50 mW cm^−2^) from the rear. Reproduced with permission [17]. Copyright 2023, Elsevier. **g** Schematic diagram of bifacial ST-PSC operation under day and night conditions, together with an image of a semitransparent perovskite film. Reproduced with permission [218]. Copyright 2024, Elsevier

The potential applications of ST-PSCs cover both standalone and integrated systems. As single systems, they can be utilized in BIPV, VIPV, agrivoltaics, wearable electronics, and indoor applications, where partial transparency and light management are key advantages. In combined systems, ST-PSCs can function as top cells in tandem configurations or as the PV component in photoelectrochemical (PV–PEC) devices, enabling efficient solar energy conversion across broader spectral ranges. Figure 19a summarizes the PCE and AVT criteria required for ST-PSCs to serve these applications, based on considerations of power output adequacy, transparency demands, LCOE competitiveness, and relevant regulatory constraints. In this context, AVT values below 50% are only practical when accompanied by PCEs exceeding 10%, primarily to maintain LCOE competitiveness.

### Tandem

According to the Shockley–Queisser theory, the maximum PCE of an ideal PSC is limited to about 33%, primarily due to sub-bandgap photon transmission and hot-carrier thermalization losses [132]. A practical strategy to overcome this theoretical ceiling is the development of tandem solar cells, which can be realized in two-terminal 2 T monolithic, three-terminal 3 T, or four-terminal 4 T mechanically stacked configurations (Fig. 19b). Within this framework, ST-PSCs are ideal as top subcells because they efficiently harvest high-energy photons (short wavelengths) while transmitting low-energy photons (long wavelengths) to the bottom subcell for further absorption. In principle, 2 T tandem architectures containing ST-PSCs with suitable band gap combination can reach PCEs of up to 46% (Fig. 19c) [213]. The theoretical efficiency of infinite-junction tandems can achieve approximately 65% under non-concentrated illumination and 83% under concentrated illumination [214]. To date, certified PCE of 34.9% [16] and 33.1% [215] has been reported for 2 T and 4 T double-junction cells on rigid susbtrates, respectively, while flexible tandem devices have demonstrated PCE of up to 33.6% [216]. These achievements confirm that the use of ST-PSCs in tandem configurations can surpass the Shockley–Queisser limit of single-junction solar cells.

In 2 T tandems, the subcells are series connected through an interconnection layer, and according to Kirchhoff’s law, the total V_OC_ equals to the sum of the subcell voltages, while the J_SC_ is constrained by the subcell with lower current. Achieving current matching therefore requires precise band gap tuning of the perovskite top cell to balance its own absorption with light transmission to the bottom cell. Because most tandem-compatible bottom cells, such as SHJ and CIGS, have negative front contacts and rough surfaces, the perovskite must have compatible device configuration and form a conformal film to ensure proper current flow and suppress interfacial non-radiative recombination [132, 219]. Consequently, ST-PSC fabrication strategies require some modifications, particularly for the perovskite and bottom charge transport layers. Encouragingly, these adjustments mostly depend on deeper technical understanding of the ST-PSC rather than high-cost interventions, enabling rapid advancement of the 2 T tandem technology.

In 4 T configurations, the subcells are electrically independent with separate terminals, which allows them to operate at their respective maximum power points without current-matching constraints. This enables ST-PSCs to be directly integrated as “add-on devices,” albeit with higher optical losses and cost due to the additional interfaces and materials [220]. Despite these drawbacks, 4 T tandems incorporating ST-PSCs hold strong potential, as they enable scalable and straightforward upgrades of existing solar farms and BIPV systems where module replacement is difficult. Since 4 T systems are free from current-matching constraints, ST-PSCs can pair with any bottom-cells regardless of the technology (e.g., TOPCon, PERC, IBC) [217, 221]. Electrical independence also reduces the sensitivity of 4 T systems to spectral variations due to diurnal and seasonal changes, potentially leading to higher energy yield and lower cost-per-watt than the 2 T systems [222]. This advantage could become more pronounced in vertical installations such as BIPV systems, where light incidence is dominated by morning or evening illumination. Even with ongoing stability concerns, ST-PSCs remain advantageous because the degraded or lower-efficiency top cells can be replaced with new or higher-efficiency ones without interrupting power generation from the underlying modules [167, 223].

Recently, 3 T tandem systems have attracted attention for combining the structural simplicity of 2 T designs with the operational flexibility of 4 T tandems. In these devices, the ITO interlayer between the top cell and bottom cell functions as both a recombination layer and a shared contact, eliminating the need for precise current matching and reducing optical losses compared to 2 T and 4 T tandems. Independent fabrication of the sub-cells further enhances manufacturing flexibility [224]. To date, PCE exceeding 29% has been achieved by combining ST-PSC with TOPCon silicon in a 3 T configuration, highlighting the strong potential of this approach [225].

ST-PSCs have been successfully paired with various bottom subcells, including silicon [226], CIGS [227], narrow-bandgap perovskites [228], polymers [229], quantum dots [230], dyes [231], and CdTe [232]. The optimal band gap for ST-PSC top cells depends on the type of the bottom device, typically ranging from 1.67 to 1.75 eV for silicon tandems, 1.59 to 1.75 eV for CIGS, and 1.8 to 1.9 eV for all-perovskite tandems [101, 132]. Assuming complete photon absorption above the band gap and full transmission below it, these band gap ranges correspond to AVT of roughly 0–40%. Recently, 2T triple-junction tandem cells based on perovskite/perovskite/silicon, perovskite/perovskite/organic, and all-perovskite architectures have been successfully developed [233]. Triple-junction devices can also be realized by integrating an ST-PSC on top of an existing 2 T tandem stack [220]. Owing to their wide band gap tunability, high versatility, and facile processing, ST-PSCs make the realization of low-cost, high-efficiency multi-junction tandem solar cells increasingly feasible.

Interestingly, when used as top subcells, ST-PSCs can also enhance the stability of less durable bottom cells. Liu et al. showed that polymer solar cells exhibited approximately 10% higher stability after 120 h of illumination when filtered through a CsPbI_2_Br-based ST-PSC (Fig. 19d) [217]. This improvement arises from the ability of the perovskite layer to filter high-energy UV photons, thereby preventing bond cleavage and photochemical degradation in the polymer layer. Overall, these attributes highlight the immense potential of ST-PSCs in advancing next-generation tandem PV technologies.

### Bifacial Photovoltaic

The silicon PV industry is undergoing a major transition toward bifacial technology, where bifacial modules production is projected to exceed 90% by 2030 [234]. In line with this industrial direction, research efforts have increasingly focused on developing bifacial perovskite solar devices. ST-PSCs possess intrinsic bifacial capability, allowing light harvesting from both the front and rear sides of the device [235]. Compared with conventional monofacial PSCs, this configuration increases power generation per unit area by utilizing diffuse and reflected sunlight (Fig. 19e) [117]. Similar to bifacial silicon modules, bifacial ST-PSCs can be mounted on elevated horizontal structures to capture albedo from the surrounding surfaces or installed vertically to harvest light from both sides. Furthermore, when integrated into BIPV systems, bifacial ST-PSCs can generate electricity not only from sunlight during the day but also from indoor and street lighting at night (Fig. 19f) [218].

For bifacial applications, it is not essential for light to fully transmit through the ST-PSC. The key objective is to maximize the light absorption from the front and rear sides, thereby increasing overall photon utilization and power output. Hence, the perovskite layer can be relatively thick (up to approximately 1 µm), rendering the device nearly opaque [78]. The layer thickness should be sufficient to absorb most incident light in a single pass, as transparent rear electrodes do not reflect light back into the active region as effectively as opaque metal electrodes [17]. Nevertheless, the rear electrode must remain transparent to allow light entry from the back side, which aligns with the typical ST-PSC configuration. For bifacial devices, a narrower bandgap (< 1.7 eV) is also preferred to enhance light absorption and improve PCE from both sides. Additionally, adopting ST-PSCs for bifacial applications tends to result in improved stability, as the perovskite composition typically excludes mixed halides, thereby avoiding issues related to photoinduced halide segregation [218].

Recent advances show that bifacial single-junction ST-PSC has energy outputs comparable to those of perovskite-based tandem devices [117]. For example, Jiang et al. showed that the ST-PSC device achieved PCEs of 23.3% (front) and 21.3% (rear), corresponding to a bifaciality factor exceeding 90% [17]. The slightly reduced rear performance was attributed to parasitic absorption in the IZO electrode and C60 layers. When illuminated from both sides, stabilized bifacial power output densities reached 26.9, 28.5, and 30.1 mW cm^−2^ at albedos of 0.2, 0.3, and 0.5, respectively (Fig. 19g). These results surpass those of the world-record monofacial single-junction PSCs and approach the performance of tandem technologies. Although the use of transparent back contact increases manufacturing costs by about 5%, bifacial ST-PSCs are projected to deliver 10%–20% higher energy yield and a lower LCOE, indicating their potential for high-efficiency and cost-effective solar power generation.

### Building-Integrated and Building-Attached Photovoltaics (BIPV and BAPV)

Since large-scale solar power plants are typically located far from urban areas where electricity demand is highest, transmitting electricity over long distances results in significant energy losses and increased costs for grid infrastructure [47]. A practical solution to mitigate these issues is to promote on-site solar power generation within urban environments using building-integrated or building-attached PV (BIPV or BAPV) systems. BIPV systems incorporate PV elements directly into the building envelope during construction or major renovations, eliminating the need for additional mounting structures. In contrast, BAPV systems are installed onto existing buildings after construction is complete using mounting brackets or frames [236]. ST-PSCs can be implemented through either approach and are particularly well suited for glass-based structures such as windows, skylights, and glazed façades.

In dense urban environments with numerous high-rise buildings, vertical façades receive significant solar exposure throughout the day. Although their orientation results in lower irradiance compared to horizontal rooftops, the large façade area allows integrated ST-PSCs to produce comparable or even greater overall energy yields. Given that the window-to-wall ratio in green building designs typically ranges from 30% to 45% [237–239], a substantial portion of building surfaces is potentially suitable for ST-PSC integration.

**Fig. 20:**
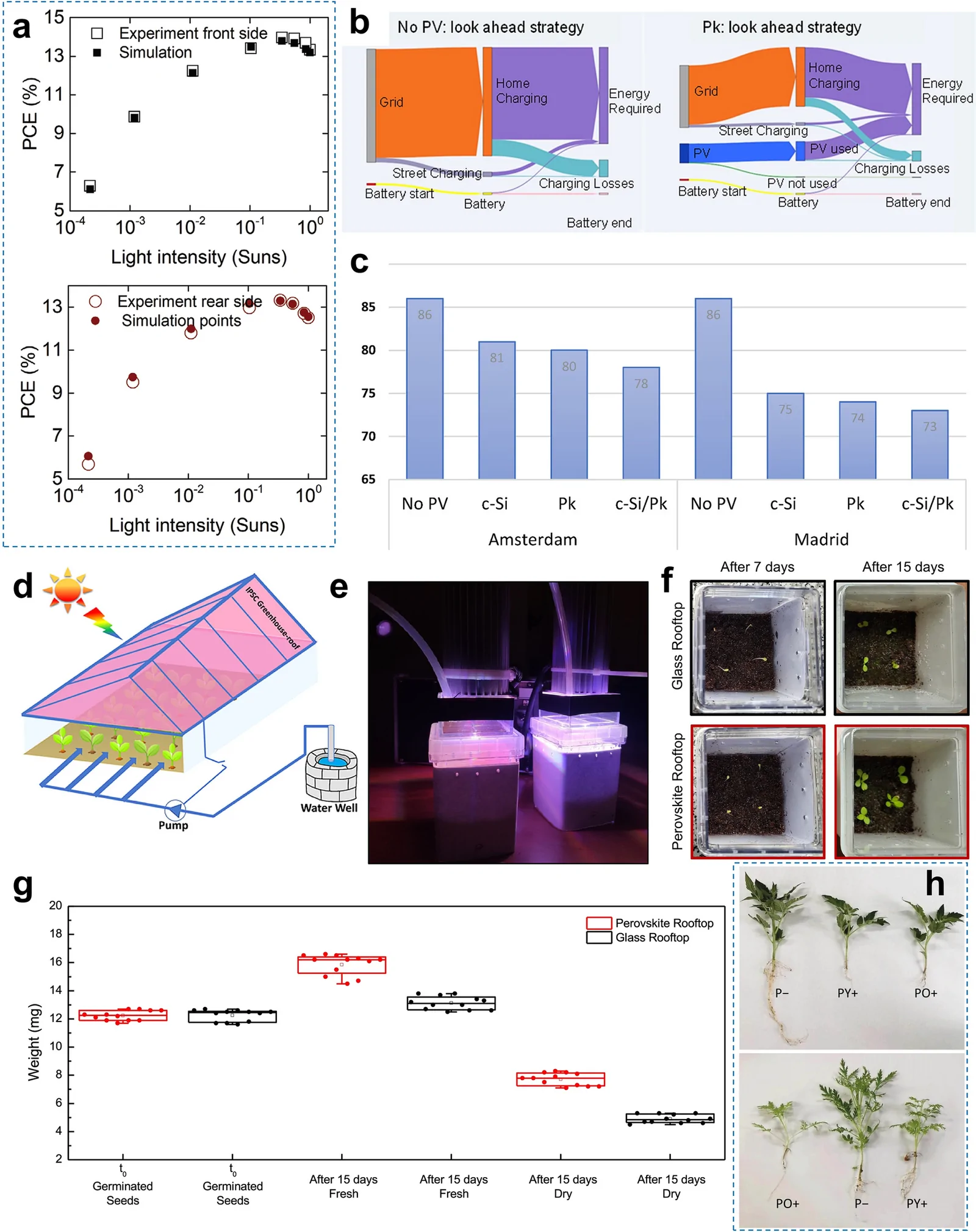
**a** PCE of experimental and simulated ST-PSC under different light intensities for front illumination and rear illumination. Reproduced under terms of the CC-BY-NC-ND license [242]. Copyright 2021, The Authors, published by Wiley. **b** Model of energy flows in EVs without and with perovskite-based PV. **c** Number of charging events for EVs without and with various PV technologies in Amsterdam (lower irradiance) and Madrid (higher irradiance). C-Si, Pk, and c-Si/Pk represent crystalline silicon, perovskite, and crystalline silicon/perovskite tandem PV technologies, respectively. Reproduced with permission [243]. Copyright 2024, Elsevier. **d** Schematic diagram of greenhouse incorporating ST-PSC roof. Reproduced under terms of the CC-BY license [244]. Copyright 2022, The Authors, published by American Chemical Society. **e** Photographs of a laboratory-scale greenhouse where radicchio seedlings are cultivated under LED illumination, with ST-PSC and bare glass serving as rooftops. **f** Photographs of the respective radicchio seedlings after 7 and 15 days. **g** Weight of the radicchio seedlings on day 1 and after 15 days. Reproduced under terms of the CC-BY-NC-ND license [245]. Copyright 2025, The Authors, published by Springer Nature. **h** Photographs of tomato (top) and Artemisia (bottom) plants grown under transparent glass (P −), a 2.53 eV perovskite filter (PY +), and a 2.31 eV perovskite filter (PO +). Reproduced under terms of the CC-BY license [246]. Copyright 2025, The Authors, published by MDPI

Conventional PV technologies are relatively heavy due to additional glass panels, metal racking, and mounting structures, which can raise safety concerns for existing buildings with limited load-bearing capacity [240]. In contrast, ST-PSCs are composed of ultrathin layers that can be fabricated on flexible, lightweight substrates, eliminating the need for bulky support structures and offering a safer solution for BAPV system. For new constructions incorporating BIPV, the solution processability of ST-PSCs enables direct fabrication of the device on architectural glass used in building assembly. Moreover, the need to encapsulate ST-PSC for protection against moisture and UV degradation naturally leads to glass-based architectures, making them highly compatible with façade integration [23]. ST-PSCs also operate efficiently under low-light and diffuse illumination, making them suitable for cloudy or shaded conditions [23, 49, 241]. As shown in Fig. 20a, the PCE of ST-PSCs illuminated from either the front or rear side slightly increases as light intensity decreases from 1 Sun to approximately 0.5 Sun and then drops sharply at very low intensities [242].

In general, an AVT over 25% is considered suitable for window application [12]. However, specific requirements may differ depending on regional green building standards. For example, Malaysia’s Green Building Index (GBI) specifies in MS1525:2014 Clause 5.4.2 that the visible light transmittance of daylight fenestration systems should not be less than 30% [247]. This requirement can simply be achieved using ST-PSCs through device engineering strategies such as employing ultrathin layers, integrating micropatterns, or utilizing wide-bandgap perovskite materials.

When integrated into glazing systems, ST-PSCs can reduce light transmission to enhance visual comfort while maintaining power generation. By allowing natural daylight penetration, ST-PSCs can also reduce the need for artificial lighting, thereby lowering overall energy consumption [30]. Additionally, ST-PSCs can act as thermal filters, blocking infrared radiation to reduce heat gain and indoor cooling demand, which helps offset their lower power output [248]. Beyond functionality, the color tunability of ST-PSCs offers architectural flexibility, allowing them to serve as both energy-generating and aesthetic elements in modern buildings [249].

### Vehicle-Integrated Photovoltaics (VIPV)

The prospect of integrating ST-PSC into vehicle glass is highly promising, particularly in response to the growing demand for electric vehicles (EVs) in recent years. ST-PSC can be incorporated into various glass components of passenger automobiles, including windshields, side and rear windows, and sunroofs [250]. Their application is even more practical in larger vehicles such as buses, trains, and trucks, where the increased glass surface area potentially allows for higher energy generation. In marine transportation, the typically unobstructed exposure of glass areas on ships, boats, and hovercraft presents favorable conditions for consistent solar energy harvesting using ST-PSC. Since ST-PSC can also be fabricated as lightweight and flexible devices, they can be integrated into aircraft windows to provide supplementary energy during flight. This technology can be either directly integrated into the glass components during vehicle manufacturing or applied as a post-processing film, similar to conventional window tints, with minor modifications to accommodate electrical connections.

Overall, this technology offers a unique opportunity to supply extra power for auxiliary systems in the vehicles such as lighting, ventilation, and infotainment, thereby reducing the load on the central battery while maintaining sufficient transparency to ensure clear visibility. Lowering the battery load may help extend vehicle driving range and, in the case of EVs, reduce recharging frequency [251]. Similar to BIPV systems, ST-PSC used in VIPV also offer the added benefit of shading, thereby reducing interior heat buildup and improving thermal comfort. Their transparency can also be tuned to accommodate specific customer preferences or regulatory design requirements, albeit at the expense of power output. While AVT values down to 0% are legally permitted in certain regions, a minimum AVT of ~ 20% is recommended to maintain adequate passenger visibility and comfort.

Recently, a research group associated with the Alliance for Solar Mobility (ASOM) presented a model demonstrating how PV installation on EVs can affect their energy usage [243]. As shown in Fig. 20b, nearly all of the energy consumed by EVs typically comes from external charging, primarily from home charging and to a lesser extent from street charging. When perovskite-based VIPV is implemented, around one-third of the energy demand is offset by energy generated from the onboard solar panels, which subsequently reduces the number of charging sessions required throughout the year. This reduction, however, still depends on the efficiency of the installed PV technology and the geographic location of the vehicle (Fig. 20c). Although these data do not directly represent a VIPV system based entirely on semitransparent devices, they still provide useful insights and highlight the potential of ST-PSC for future VIPV applications.

### Agrivoltaics

Agrivoltaics is an innovative strategy that enables dual land use, in which solar panels not only generate clean electricity but also provide partial shading for plants or aquatic animals, thereby enhancing their growth and overall productivity [252]. However, the conventional agrivoltaic systems based on opaque silicon panels can intensify the shading effect, potentially inhibiting the crop growth. Unlike opaque panels, ST-PSC offers selective light transmission, as their band gap can be tuned to allow specific wavelengths of sunlight to pass through, particularly those needed for photosynthesis, thereby meeting the crops optimal light requirements. ST-PSC prepared on flexible and lightweight substrates is also suitable for greenhouse roofs, where the canopy brackets are typically designed to support only lightweight plastic coverings (Fig. 20d). Moreover, the heat island effects in greenhouses could be mitigated using ST-PSC with wide-band-gap perovskite, because thermalization losses from non-radiative charge relaxation during high-energy photon absorption are minimized [244]. Most importantly, the ST-PSC not only ensures sufficient energy for self-operations of the agricultural site, but also enables farmers to sell surplus electricity to the grid, thereby supporting rural economic development.

During plant growth, photosynthetically active radiation, which covers the wavelengths from 400 to 700 nm, is the critical spectral range that plants utilize for photosynthesis [253]. Since most plants ineffectively absorb or mostly reflect the green light in the wavelength range of 500–600 nm (causing their green appearance), this underutilized light can be harnessed by ST-PSC for electrical power generation [252]. Perovskite materials with an absorption edge around 600 nm such as CsPbI_x_Br_1-x_ and MAPbBr_3_ show great potential for this purpose [160, 244]. Interestingly, the use of ST-PSC can also support healthy plant growth beneath them, as demonstrated by Spampinato et al. [245]. They developed a CsPbI_3_-based ST-PSC that transmits 70%–80% of below-band-gap photons (32% in the visible range). The light filtered through the device exhibited reduced blue light, enhanced red light, and a lower red to far-red ratio. When applied in laboratory-scale greenhouses to grow radicchio seedlings (*Cichorium intybus var. latifolium*), the filtered light promoted the growth of larger leaf area and increased biomass (Fig. 20e, f). After 15 days of growth, the average seedling mass increased from 13 mg under bare glass to 16 mg under ST-PSC-filtered light (Fig. 20g). These findings indicate that ST-PSCs can beneficially modulate seedling photomorphogenesis while simultaneously enabling energy harvesting.

Consistent with these findings, industrial-scale demonstrations suggest that moderate shading introduced by perovskite-based panels does not adversely affect crop performance. For example, Promate Group, a Taiwan-based company, reported that shading levels of up to 30% did not compromise the growth of water spinach, romaine lettuce, leaf lettuce, or tomato crops [254]. However, it is worth mentioning that the benefits of ST-PSCs are not universal across plant species. Preliminary tests on tomato (*Solanum lycopersicum L.*) and Artemisia (*Artemisia annua L.*) exhibited approximately 50% reductions in aerial biomass when a 2.53-eV perovskite absorber filter was used, with an additional 10% reduction observed when the band gap was further lowered to 2.31 eV (Fig. 20h) [246]. Overall, ST-PSCs hold immense potential for agrivoltaic applications as it offers simultaneous energy generation and crop cultivation, although careful and crop-specific optimization of key parameters such as AVT, absorber band gap, and shading ratio is needed to fully realize their benefits.

### Wearable Electronics and Indoor Applications

**Fig. 21:**
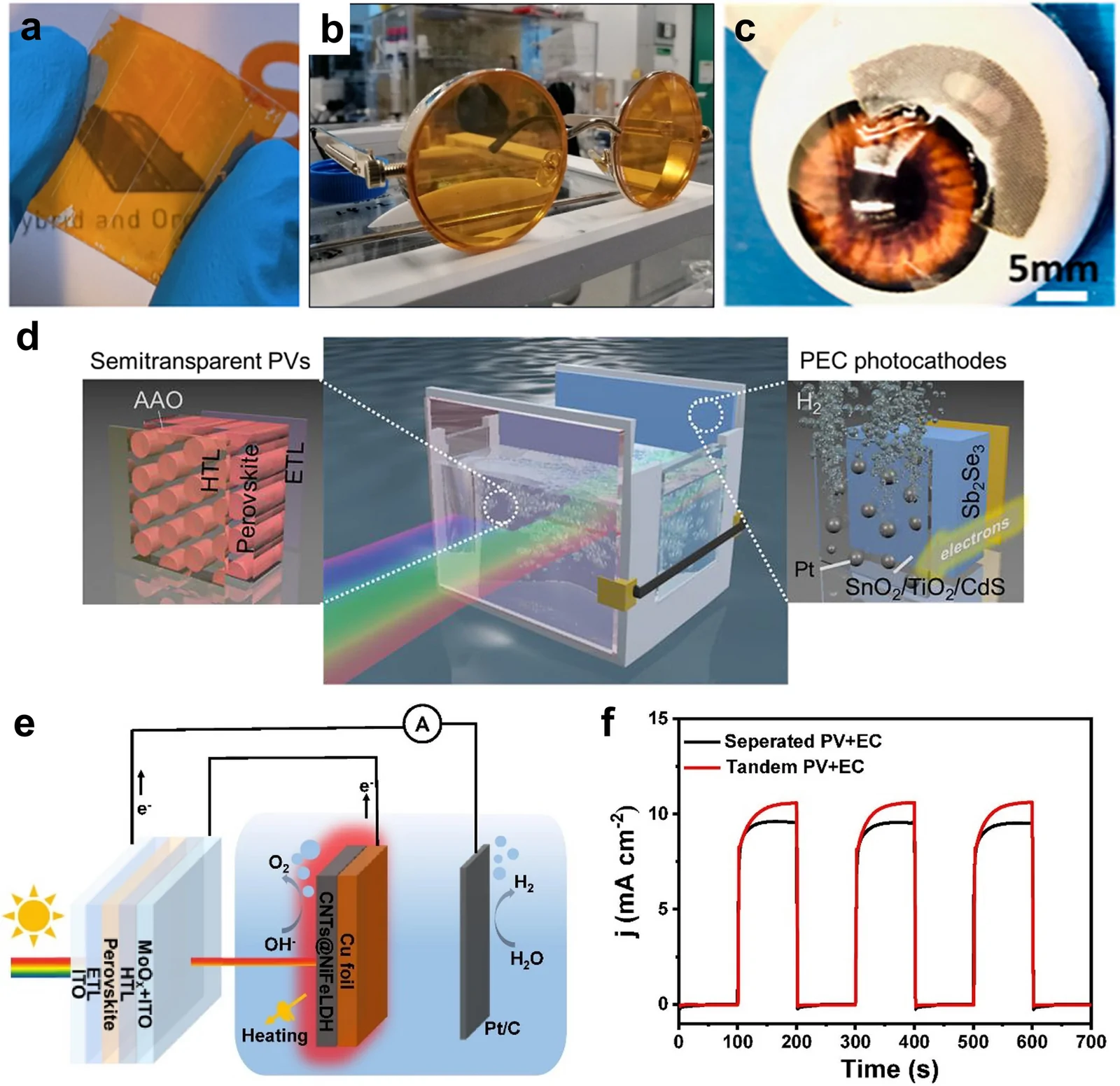
**a** Photograph of a flexible ST-PSC. Reproduced with permission [49]. Copyright 2024, American Chemical Society. **b** Photograph of eye glasses based on FAPbBr_3_ ST-PSCs. Reproduced with permission [255]. Copyright 2021, American Chemical Society. **c** Smart contact lenses with integrated solar cells. Reproduced under terms of the CC-BY-NC-ND license [256]. Copyright 2022, The Authors, published by American Chemical Society. **d** Schematic of the PEC system integrated with a ST-PSC for water splitting. Reproduced with permission [257]. Copyright 2020, Royal Society of Chemistry. **e** Schematic of the EC system integrated with a ST-PSC for water splitting. **f** I–t curves of the EC system under chopped simulated sunlight, illustrating the photothermal effect on the electrocatalyst. Reproduced with permission [258]. Copyright 2023, Elsevier

Another attractive feature of ST-PSCs is their potential for integration into wearable electronics and indoor applications. The deployment of ST-PSCs in lightweight, flexible form allows them to conform to curved or irregular surfaces, making them both comfortable to wear and easy to handle (Fig. 21a) [49]. When incorporated into wearable devices, ST-PSCs provide a continuous, self-sustaining power supply, reducing the inconvenience of frequent recharging and enhancing the overall user experience. For example, smart glasses and smart contact lenses can harvest sufficient power for recharging while maintaining clear visual perception during daily activities such as driving or reading (Fig. 21b) [255, 256]. ST-PSCs also show potential for applications such as camping tents, health-monitoring textiles, smart homes, and IoT systems, where they can be integrated as visually appealing power sources that provide continuous energy for daily use, thereby enhancing the quality of life (Fig. 21c). The power requirements of small electronic devices can be met even at PCE values as low as ~ 1% [24].

More importantly, ST-PSCs exhibit higher PCEs under indoor lighting than under standard 1 Sun illumination, although the total power output remains limited by the lower intensity of indoor light sources. Guerrero et al. reported that the PCE of their device increased by up to 50% under 1000 lx illumination compared to 1 Sun conditions [259]. Han et al. further confirmed this trend by observing that the efficiency of ST-PSCs under indoor lighting was approximately 60%–70% higher than under 1 Sun conditions [218]. The PCE enhancement is attributed to the spectral characteristics of indoor light, which is enriched in blue photons that can be effectively harvested by perovskites [260]. Overall, these findings underscore the strong potential of ST-PSCs for wearable and indoor energy-harvesting applications.

### PV-(P)EC

Photoelectrochemical (PEC) water splitting is a promising technology for producing hydrogen as a green and economical transportable fuel. A PEC cell typically comprises a semiconductor photoelectrode immersed in an aqueous electrolyte, where light absorption generates electron–hole pairs that drive the oxygen and hydrogen evolution reactions (OER and HER) [261]. ST-PSCs can also be connected in series with the photoelectrode to supply additional voltage for water splitting. Conventionally, an opaque PSC is placed behind the PEC reactor containing a wide-bandgap photoelectrode, allowing unabsorbed light from the photoelectrode to pass through the reactor and be harvested by the PSC. In contrast, ST-PSC can be placed in front of the PEC reactor, allowing low-energy light to pass through to the photoelectrode (Fig. 21d). This front-side placement broadens the range of usable photoelectrode materials in the PEC cell, enabling the integration of low-bandgap semiconductors such as silicon and Sb_2_Se_3_ [257].

The use of ST-PSC not only enhances the fraction of solar energy harvested, but also ensures that the PEC system does not rely on additional voltage from external source. Nonetheless, similar to tandem PV, the bandgap of perovskite in the ST-PSC must be tuned to maximize the photocurrent generated and hence the solar-to-hydrogen efficiency (*η*_STH_) of the PEC system, which is critical for surpassing the commercialization target of 10% [262]. For instance, silicon-based photoelectrode combined with ST-PSC containing perovskite with moderate bandgap of 1.62 eV enables a photocurrent of 11.5 mA cm^−2^ (corresponds to *η*_STH_ of 14.1%) for the PEC process. On the contrary, a higher photocurrent of 14.3 mA cm^−2^, which corresponds to *η*_STH_ of 17.6%, was obtained when perovskite with 1.75 eV bandgap was used [263].

In electrochemical (EC) system, ST-PSC can indirectly induce photothermal effect that boosts the overall water splitting process. Since perovskite does not absorb the below-bandgap photons, ST-PSC transmits most of the unabsorbed infrared light to the EC system, where carbon-based electrodes absorb it and generate localized heating (Fig. 21e). The resulting photothermal effect raises the temperature of the electrode and the nearby electrolyte, which reduces the Gibbs free energy required for water splitting and improves ionic transport at the electrode–electrolyte interface, thereby boosting the OER [264]. As shown in Fig. 21f, ST-PSC indirectly induces photothermal effect on a carbon nanotube/NiFe layered double hydroxide (CNT@NiFe LDH) electrode, which increases the *η*_STH_ from 11.7% to 13.2% [258]. These results demonstrate the strong potential of ST-PSC for integration into PEC and EC water splitting systems for green hydrogen production.

## Technoeconomic Overview

### Manufacturing Costs

**Fig. 22:**
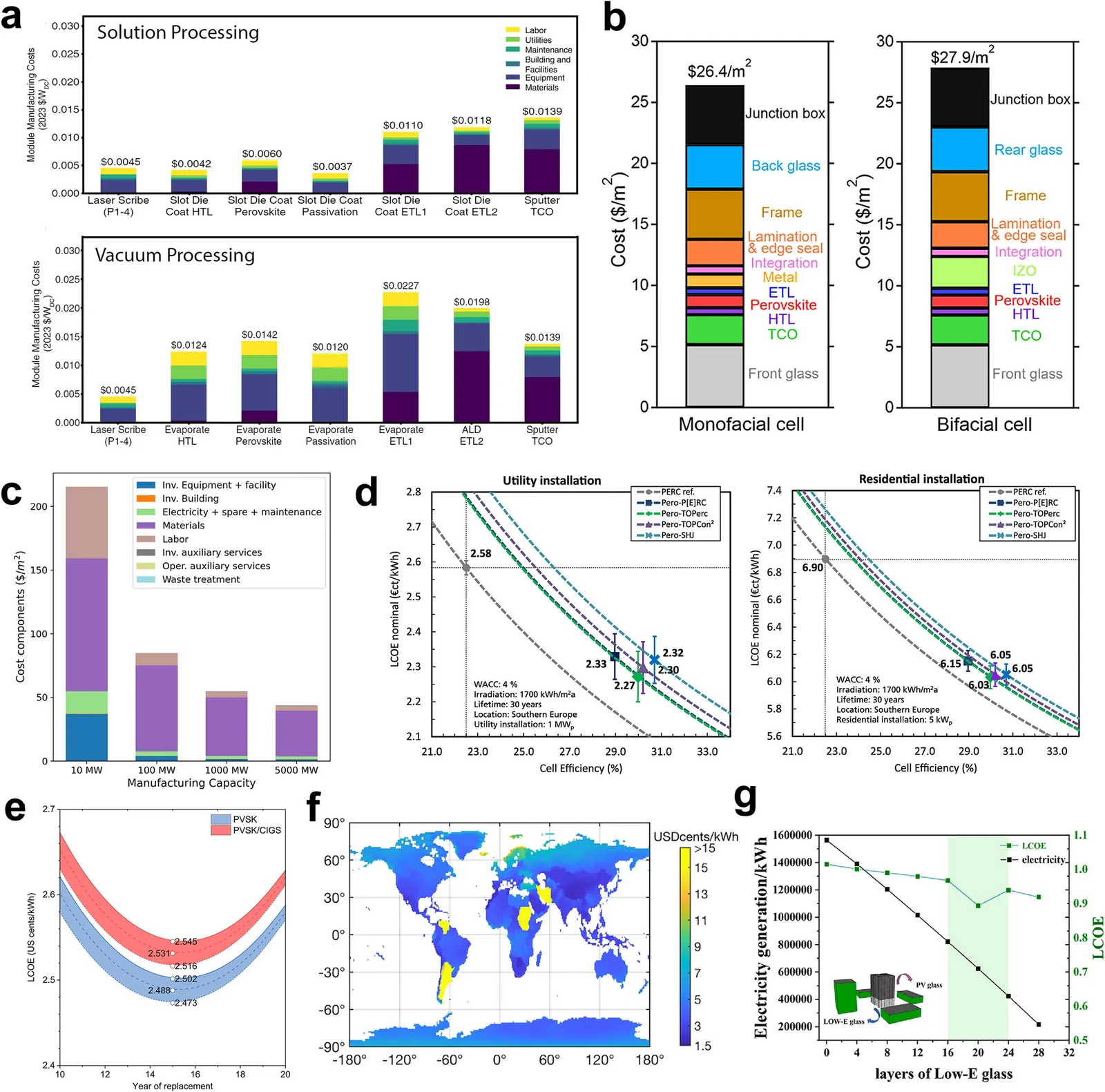
**a** Manufacturing cost of ST-PSC produced by solution- and vapor-based processes. Reproduced with permission [222]. Copyright 2024, Elsevier. **b** Manufacturing cost breakdown of individual components in monofacial (opaque) and bifacial (ST-PSC) cells. Reproduced with permission [17]. Copyright 2023, Elsevier. **c** Manufacturing cost breakdown of perovskite devices as a function of production capacity. Reproduced under terms of the CC-BY license [265]. Copyright 2025, The Authors, published by Elsevier. **d** LCOE of ST-PSC-based tandem systems for utility- and residential-scale applications. Reproduced under terms of the CC-BY-NC license [266]. Copyright 2020, The Authors, published by Wiley. **e** LCOE of tandem and ST-PSCs under module replacement scenarios. Reproduced with permission [267]. Copyright 2022, American Chemical Society. **f** Region-dependent LCOE of ST-PSC. Reproduced with permission [268]. Copyright 2023, Elsevier. **g** Electricity generation and LCOE of a simulated building integrating low-E glass and ST-PSC at different floor levels. Reproduced with permission [269]. Copyright 2025, Elsevier

The total cost of a photovoltaic system includes manufacturing, installation, operation, and other soft costs, with manufacturing currently dominating the cost of semitransparent perovskite solar cells (ST-PSCs). Manufacturing cost is mainly determined by material choice and fabrication methods. In general, ST-PSCs fabricated via vapor-based techniques are more costly than solution-processed devices due to the high cost of specialized equipment, lower throughput, and expensive precursors such as C60, TDMASn, and ceramic sputter targets. As shown in Fig. 22a, ST-PSCs produced entirely by vapor-based methods (evaporation, ALD, and sputtering) have an estimated manufacturing cost of about $0.100 W^−1^ excluding the substrate, which is nearly twice that of solution-processed devices using slot-die coating (~ $0.055 W^−1^) [222].

At the module level, more than half of the total cost originates from encapsulation components, including lamination, the frame, rear glass, and the junction box. At the device level (the stack from glass substrate to rear electrode), costs are dominated by the TCO-coated glass, followed by the rear electrode. ST-PSCs generally incur slightly higher manufacturing costs than opaque PSCs, as illustrated by the cost breakdown of monofacial opaque and bifacial ST-PSC modules in Fig. 22b [17]. This increase is mainly due to the expensive indium-based transparent rear electrodes, driven by limited indium supply [132] and strong global demand, particularly in the electronics sector [270]. Replacing these electrodes with indium-free alternatives and development of buffer-layer-free device can significantly reduce the costs. For example, carbon-based electrodes have been reported to lower material costs by ~ 70% relative to ITO, although further work is required to ensure scalability [75].

Meanwhile, economies of scale play a crucial role in determining ST-PSC production costs. As annual manufacturing capacity increases from the megawatt to gigawatt scale, the cost per unit area (USD m^−2^) typically decreases, with reported reductions of around 75%–80% [265]. As shown in Fig. 22c, material costs dominate the total manufacturing expenses across all production capacities. In contrast, the labor, equipment, and facility investments decrease substantially with increasing production volume, reflecting more efficient use of labor and the ability to spread fixed costs over more modules. Utilities and maintenance costs also decline, while components such as depreciation of buildings and auxiliary services, operational costs of auxiliary systems, and waste treatment remain minor regardless of scale. Although these data pertain to opaque PSCs, the trend is generally applicable to ST-PSCs.

### Levelized Cost of Electricity

In techno-economic analyses of PV systems, the LCOE is a widely accepted metric for evaluating cost competitiveness. It is defined as the total lifetime cost of building and operating a system divided by the total electricity generated, typically expressed in $/kWh [271]. Simply put, lower LCOE indicates a more cost-effective system for converting sunlight into electricity. LCOE of PV technologies including ST-PSCs are majorly influenced by the PCE, lifetime (or stability), and total system costs [168].

Despite higher manufacturing costs, the cost-effectiveness of ST-PSCs compared with opaque PSCs varies with the application. For bifacial power generation, ST-PSCs often achieve lower LCOE than opaque modules with comparable efficiency and lifetimes because they effectively capture light on both front and rear sides, thereby increasing the energy output and lowering the cost per kWh. For instance, a ST-PSC with a 20-year operational lifetime, 22% front-side efficiency, and 90% bifaciality factor can achieve ~ 15% lower LCOE than a comparable monofacial opaque PSC [17]. In tandem applications, incorporating ST-PSCs as the top subcell, particularly in 2 T configurations, enables lower LCOE in both utility- and residential-scale systems, as the efficiency gains of tandem architectures outweigh the additional manufacturing costs (Fig. 22d) [266]. With proper optimization, ST-PSCs used in 4 T tandem can also exhibit lower manufacturing costs and higher operational energy yield than 2 T tandems, thereby contributing to a further reduction of LCOE [222, 272, 273]. By contrast, in BIPV systems, high AVT requirements typically reduce PCE and increase LCOE, with practical deployment generally requiring a minimum module PCE of ~ 15%, corresponding to a peak AVT of ~ 55%. Nevertheless, higher AVT at lower PCE can remain economically viable, as benefits from building thermal energy management and enhanced aesthetics can partially offset the increased LCOE.

Generally, perovskite technology can potentially achieve lower LCOE than silicon, but short operational lifetimes substantially increase costs. Currently, achieving ST-PSCs with 20–30 years lifetimes remain challenging, thereby raising concerns about their market viability. Module replacement strategies can mitigate this issue, in which degraded ST-PSCs are replaced after a defined period of operation. Early replacement can increase the costs due to insufficient gains in power generation, whereas delayed replacement can reduce electricity output from the degraded device. Replacing ST-PSCs at an optimal time ensures that the increase in power output outweighs the additional costs, leading to a net reduction in the LCOE. Wang et al. suggested that, for single-junction or tandem systems with 30-year expected lifetime, replacing modules after 15 years allows the LCOE to reach its minimum value (Fig. 22e) [267].

Apart from the abovementioned factors, it is also important to note that the LCOE of ST-PSCs can vary substantially across regions. This variation arises not only from the differences in regional climate but also from location-dependent cost factors such as labor, utilities, tariffs, and policy incentives [222]. In particular, labor costs play a major role in influencing the LCOE when production process automation is comparable. Consequently, equatorial regions often exhibit lower LCOE than those at higher latitudes due to higher solar irradiance and often lower labor costs (Fig. 22f) [268]. In some regions, exceptionally high LCOE can also arise from adverse political or economic conditions. Understanding region-dependent LCOE is crucial for realistic techno-economic assessments and for optimizing ST-PSC deployment in specific markets.

More importantly, the LCOE of ST-PSCs depends on the installation scenario, with the lowest values achievable through optimized placement of the modules. In BIPV systems, upper floors generally receive more sunlight, while lower floors are often shaded by surrounding buildings. Installing ST-PSCs on shaded lower floors reduces energy generation and may increase indoor lighting demand, thereby lowering the net power output. A better strategy is to combine low-emissivity (low-E) glass on lower floors with ST-PSCs on upper floors. While ST-PSCs allow partial light transmission and simultaneously generate electricity, low-E glass reflects infrared radiation to reduce heat transfer [274]. This approach lowers initial investment costs while maintaining adequate indoor lighting, resulting in a significant LCOE reduction. For example, in a 32-story building (102.4 m height) with surrounding buildings up to 70 m, installing ST-PSCs above the 20th floor (above 64 m) can reduce the LCOE by ~ 12% (Fig. 22g) [269].

Generally, opaque PSCs are expected to have lower LCOE than current silicon PV for both utility- and residential-scale applications [275]. However, current industry data suggest that single-junction opaque PSCs would surpass silicon PV in LCOE only if module efficiency and lifetime exceed 25% and 25 years, respectively [270], a target that remains challenging. This indicates that PSCs are not intended to replace silicon PV but rather to complement it. In particular, the use of ST-PSCs not only provides dual functionality in applications such as BIPV and agrivoltaics, but also helps in reducing overall system LCOE through tandem or bifacial configurations.

## Challenges

### Performance

The PCE of ST-PSCs is generally lower than that of their opaque counterparts due to multiple interrelated factors. The use of thin perovskite layer or wide-bandgap compositions to achieve high transparency significantly limits photon absorption in the active layer [78]. In addition, replacing the opaque metal electrode with a transparent electrode at the rear side reduces light back-reflection and increases series resistance [49]. Under real-world conditions, elevated device temperatures can further increase the sheet resistance of the ITO rear electrode [82]. Bandgap tuning also alters the energy-level alignment, thereby affecting charge transfer efficiency at the perovskite/ETL and perovskite/HTL interfaces. Moreover, parasitic absorption by charge transport layers, buffer layers, and transparent electrodes, along with reflectance losses, can significantly reduce the performance of ST-PSCs and their integrated systems. Consequently, most ST-PSCs developed to date exhibit LUE values well below the theoretical limit.

The theoretical LUE for wavelength-selective absorbers such as organic small molecules and polymers is 20.6% [276–278]. In contrast, perovskites are non-wavelength selective and absorb broadly across the spectrum above their bandgap, limiting their maximum LUE to about 8%. Although this value might appear sufficient for some applications, a new challenge arises when considering the trade-off between transparency and power output. Increasing the transparency can indeed enhance the LUE, but the corresponding PCE could be too low to provide sufficient power to meet the demand of the intended system. For example, an ST-PSC with 4.3% LUE and only 8.9% PCE may perform worse for power generation in BIPV system compared to an ST-PSC with 3.7% LUE and 16.2% PCE [70]. Therefore, optimizing LUE remains a major challenge and requires a careful balance between electrical efficiency and optical transparency, which is achievable through the rational selection of materials, device architecture, and optimized fabrication processes.

### Stability

Although the stability of ST-PSCs has improved substantially in recent years through extensive material innovations and advanced encapsulation strategies, their operational lifetime remains below the industrial benchmark of 25 years. The degradation of ST-PSCs has significant implications across various applications. In tandem architectures, deterioration of the top ST-PSC reduces light harvesting, thereby limiting the achievable J_SC_. This effect is particularly critical in 2 T tandem configurations, where degraded top subcells directly constrain the performance of the bottom subcells due to the monolithic electrical coupling. In 4 T tandems, degraded ST-PSCs act as unwanted optical filters that hinder light transmission to the underlying devices. Furthermore, in off-grid systems such as BIPV and PV–PEC setups, rapid degradation of ST-PSCs can lead to severe power shortages and disrupt system operation. In agrivoltaic systems, spectral shifts caused by the degraded devices may adversely affect plant growth and yield uniformity.

As perovskite materials deteriorate, visual changes often occur, with films turning yellow due to the formation of PbI_2_ [279]. This discoloration diminishes the visual appeal of applications where aesthetic values are essential, such as BIPV, VIPV, and wearable devices. Additionally, the formation of small domains and additional phase boundaries in degraded perovskite layers can induce light scattering [280], which further obstructs light transmission and compromises the intended transparency of ST-PSCs. Replacing degraded modules after only a few years poses substantial technical and economic challenges. In BIPV systems, replacing window- or façade-integrated modules in high-rise buildings requires specialized labor and incurs high costs. For commercial properties, such maintenance may cause operational downtime, while in residential settings, it can intrude upon comfort and privacy. Strategies such as advanced encapsulation, surface passivation, and the use of 2D or quasi-2D perovskites have shown promising results in mitigating the stability issues and require further attention [19, 281].

Another critical issue that has received limited attention is the effect of reverse bias during the operation of the modules. Owing to the monolithic series interconnection, a subcell in an ST-PSC module can be driven into reverse bias when its photocurrent is substantially lower than other subcells, for example, due to partial shading [282]. In this condition, the subcell is subjected to a strong internal electric field, which can induce localized electrical breakdown, resulting in a pronounced increase in leakage current. The reverse-biased subcell therefore experiences Joule heating (conversion of electrical energy into heat due to the material resistance), resulting in a temperature rise [283]. The elevated temperature not only promotes phase segregation, but also accelerates perovskite degradation, causing irreversible performance loss [205, 284]. This issue is particularly critical for ST-PSCs deployed in BIPV, VIPV, and agrivoltaic systems, where partial shading is frequent due to surrounding structures and vegetation.

Moreover, thinner perovskite films exhibit poorer stability than their thicker counterparts due to the higher sensitivity to oxygen and humidity. Certain TCOs, such as IZO, also exhibit pronounced initial burn-in during early operation, likely due to the reaction between zinc and acidic by-products from perovskite decomposition [17]. Since the degradation mechanisms in ST-PSCs differ slightly from those in opaque devices, detailed studies on materials compatibility, interfacial stability, and encapsulation effectiveness are needed before large-scale commercialization can be achieved.

### Large-Scale Fabrication

Achieving a uniform, ultrathin perovskite layer over large areas poses significant challenge in ST-PSC fabrication. Currently, most studies still focus on solution chemistry, interfacial engineering, and deposition parameters based on spin coating, yet spin coating is unsuitable for large-scale production. Therefore, scalable solution-processing techniques such as slot-die coating, blade coating, and inkjet printing have been applied, although reports on their use for ST-PSCs are still limited [165]. However, solution-processing methods involve complex perovskite precursor formulations and intricate crystallization kinetics, which complicates the reproducible fabrication of uniform films over large areas [285, 286]. Key factors such as precursor solubility, solvent compatibility with underlying layers, solution dispersibility during coating, film pre- and post-treatment, and environmental conditions are challenging to optimize particularly for ultrathin films. Moreover, solution-based processes impose additional safety and environmental burdens on the industry due to the use of hazardous solvents, which increases the requirements for waste handling and process management. Fabrication of highly conductive transparent rear electrodes using scalable, solution-based routes also remains underdeveloped, with limited evidence of reproducibility and true scalability for large-area ST-PSC.

Meanwhile, vapor-based methods such as co-evaporation and sputtering offer precise thickness control and high process reproducibility, but they are generally slower and more costly than solution-based techniques [19]. Vapor-based approaches also attract strong industrial interest owing to their established use in thin-film PV technologies such as CdTe and CIGS, but laboratory-scale research has largely focused on the solution processing due to the lower capital expenditure (capex) [287]. As a result, experimental data and fundamental insights of vapor-based perovskite deposition for ST-PSCs remain inadequate. From technical perspective, precise control of organic halide adsorption on the substrate using vapor deposition is challenging [288], which can easily lead to the formation of defects and impurities [289]. At present, a combination of solution and vapor processing is necessary to fabricate different functional layers in ST-PSCs. However, switching from solution to vapor processing introduces batch operation, as vapor deposition requires vacuum conditions, which constrains production throughput. The dilemma over the optimal choice of method continues to engage the research community. Encouragingly, unlike spin coating, both scalable solution- and vapor-based techniques can be optimized to reduce precursor waste to negligible levels [287, 290].

In monolithic ST-PSC modules, module design through laser scribing becomes more challenging, as the lower conductivity of transparent electrodes slows down the lateral charge transport. Reducing the distance between scribing (i.e., increasing the number of device stripe) on a substrate can mitigate this effect and improve transparency, but the total active area will be reduced. Thus, careful control of module layout is essential to maximize the electrical performance while minimize the cost of large area devices.

### Cost per Power-Output

Several technoeconomic studies indicate that the use of ST-PSC in bifacial and tandem configurations, particularly 4 T designs, can achieve lower LCOE values than current PV technologies, provided that the bandgap of ST-PSC is properly tuned [17, 222, 266]. However, comprehensive technoeconomic analysis of ST-PSCs for other applications is scarce, making it difficult to assess their potential for cost competitiveness. Although thinner perovskite films reduce material usage in ST-PSC, the cost savings are minimal because glass substrates and TCOs dominate the total cost of the device. Extending device lifetime could help offset efficiency losses and maintain competitive LCOE values, but achieving operational stability is still challenging.

Another major cost concern is the reliance on indium-based TCOs such as ITO, which are used as both front and rear contacts in ST-PSCs. The scarcity and high price of indium, worsened by competition from consumer electronics, display, and LED industries, could significantly elevate production costs [132]. Although alternatives like IZTO, IGTO, and ICO have been explored, they still depend on indium and thus face similar supply issues. Non-indium-based TCOs, on the other hand, have yet to match the performance or scalability of ITO. The incorporation of buffer layers adds further process complexity and cost. Therefore, developing buffer-free architectures and indium-free transparent electrodes with low cost, high conductivity, and strong optical transmittance is essential for achieving sustainable and economically viable ST-PSC technology.

### Toxicity

State-of-the-art ST-PSCs with the highest performance currently rely on lead-halide perovskites. To address concerns about lead toxicity, tin-based perovskites have been explored as a potential alternative, with the corresponding ST-PSC recording a PCE of over 10% [291]. However, such performance is still lower than that of lead-based devices, and operational stability is poor due to the high tendency of Sn^2+^ to oxidize to Sn^4+^ [292]. Halide double perovskites (e.g., Cs_2_AgBiBr_6_) and perovskite-inspired materials (e.g., bismuth or antimony (Bi/Sb)-based chalcohalides) have also been developed as lead-free alternatives. Although these materials exhibit relatively wide band gaps (generally > 1.7 eV), which are theoretically suitable for ST-PSC, they suffer from poor charge transport properties, and their indirect band gaps result in low absorption coefficients [293, 294]. For example, an ST-PSC based on the Cs_2_AgBiBr_6_ double perovskite achieved only 2.3% PCE with 31.8% AVT, corresponding to LUE of ~ 0.7%, which is far below the practical level for commercialization [295]. As a result, lead-free perovskite alternatives remain far from being commercially viable, and the toxicity of lead continues to be a major concern.

Although ST-PSCs have significantly lower lead content than conventional PSCs due to the use of ultrathin perovskite layers, the presence of lead still raises health and environmental concerns and may conflict with certain regional regulations. For example, under the EU Restriction of Hazardous Substances (RoHS) directive, lead in homogeneous materials within electronic products is limited to 0.1 wt% [296]. The legislative definition of “homogeneous material” has sparked debate over permissible lead content. If the substrate is included, rigid glass-based devices typically remain below the RoHS threshold (~ 0.035 wt%), whereas flexible devices can exceed 2 wt%, despite containing similar absolute amounts of lead, due to their much lower substrate mass [297]. If only the functional layers are considered a homogeneous material, the weight percentage of lead would be significantly higher. At present, both rigid and flexible PV modules intended for permanent installation at fixed location are exempt from RoHS restrictions [296], but this exemption does not cover consumer electronics, which limits ST-PSC adoption in wearable and indoor applications.

Furthermore, the deployed lead-based ST-PSC could be subjected to replacement during their midlife, if future regulations require the use of lead-free alternatives. Even without changes in regulation, public concern is likely to persist, as in the case of CdTe-based PV modules [298]. This is because ST-PSC applications such as BIPV, VIPV, and agrivoltaics are often in close proximity to end users or food crops. In the event of physical damage caused by environmental mechanical stress (e.g., wind, snow, thermal cycling), extreme weathers (e.g., hailstorms, hurricanes), or fires, potential lead leakage could result in direct human or agricultural contamination.

To mitigate this risk, several lead-absorbing materials have been developed to chemically sequester the lead and keep it contained within the device. These materials can be applied as additional layers on the outer or inner surfaces of the encapsulation glass [299, 300] or incorporated directly into the encapsulant [301]. For instance, a low-cost shellac coating has been reported to retain 97.87% of lead in severely damaged devices [302]. In fire scenarios, the encapsulation glass itself can capture nearly all lead as PbO or PbO_2_ species, provided the device remains fully encapsulated [303]. However, while these materials allow reasonable light transmission and effective lead absorption, the high transparency requirements of ST-PSCs may limit the thickness that can be applied, which may reduce their lead adsorption capacity. Besides, the real-world data on lead containment and leakage, particularly for ST-PSC technology, are still limited. Therefore, it is essential to develop practical and effective encapsulation technology to ensure that the advantages of ST-PSCs outweigh their lead-related risks.

### Suitable Area Constraints

In scenarios where transparency is not essential, the deployment of ST-PSC becomes impractical, as conventional opaque PSC generally offer higher PCE at a lower cost. This restricts the total surface area available for their deployment and posing a challenge to their market competitiveness. Furthermore, the surfaces suitable for ST-PSC integration often do not face optimal solar angles. For instance, in BIPV applications, large façade areas are typically vertical or slanted, which reduces the effective solar irradiance and overall energy yield. Besides, variations in the angle of sunlight incidence can lead to a reduction in PCE, as reflection losses governed by Fresnel law become more pronounced. Additionally, the building orientation may result in ST-PSC receiving sunlight during only one portion of the day (either morning to afternoon or afternoon to evening). The available areas may also be affected by shading from surrounding structures or nearby trees and plants, causing substantial variability in power output. In VIPV applications, challenges arise not only from the limited surface area and unfavorable orientation of vehicular glass, but also from the dynamic motion of vehicles, which leads to frequent changes in the angle of sunlight incidence. Therefore, simulation studies are highly encouraged to guide ST-PSC system design to maximize the usable area, thereby increasing the energy output while preserving the desired light transmittance.

## Conclusion and Future Perspectives

ST-PSCs have achieved remarkable progress in uniting optical transparency with high PV performance, positioning them as a promising platform for multifunctional solar technologies. Building on this momentum, this review has outlined recent advances in both small- and large-area ST-PSCs, while highlighting their diverse prospects for future real-world integration. Although the theoretical LUE of ST-PSCs is constrained to approximately 8% due to their non–wavelength-selective nature, the attainable AVT already satisfies the requirements for most practical applications. The intrinsic tunability of transparency empowers ST-PSCs to bridge the gap between aesthetics and functionality, enabling integration into single-purpose systems such as bifacial, BIPV, and agrivoltaics, as well as multifunctional combined architectures like tandem and PV-PEC devices that unite energy conversion with additional functionalities. Recent demonstrations on both rigid and flexible substrates, along with significant progress in large-scale fabrication, further substantiate the feasibility of ST-PSCs for practical deployment. Looking ahead, several future perspectives are critical for the continued development of ST-PSCs.

**Fig. 23:**
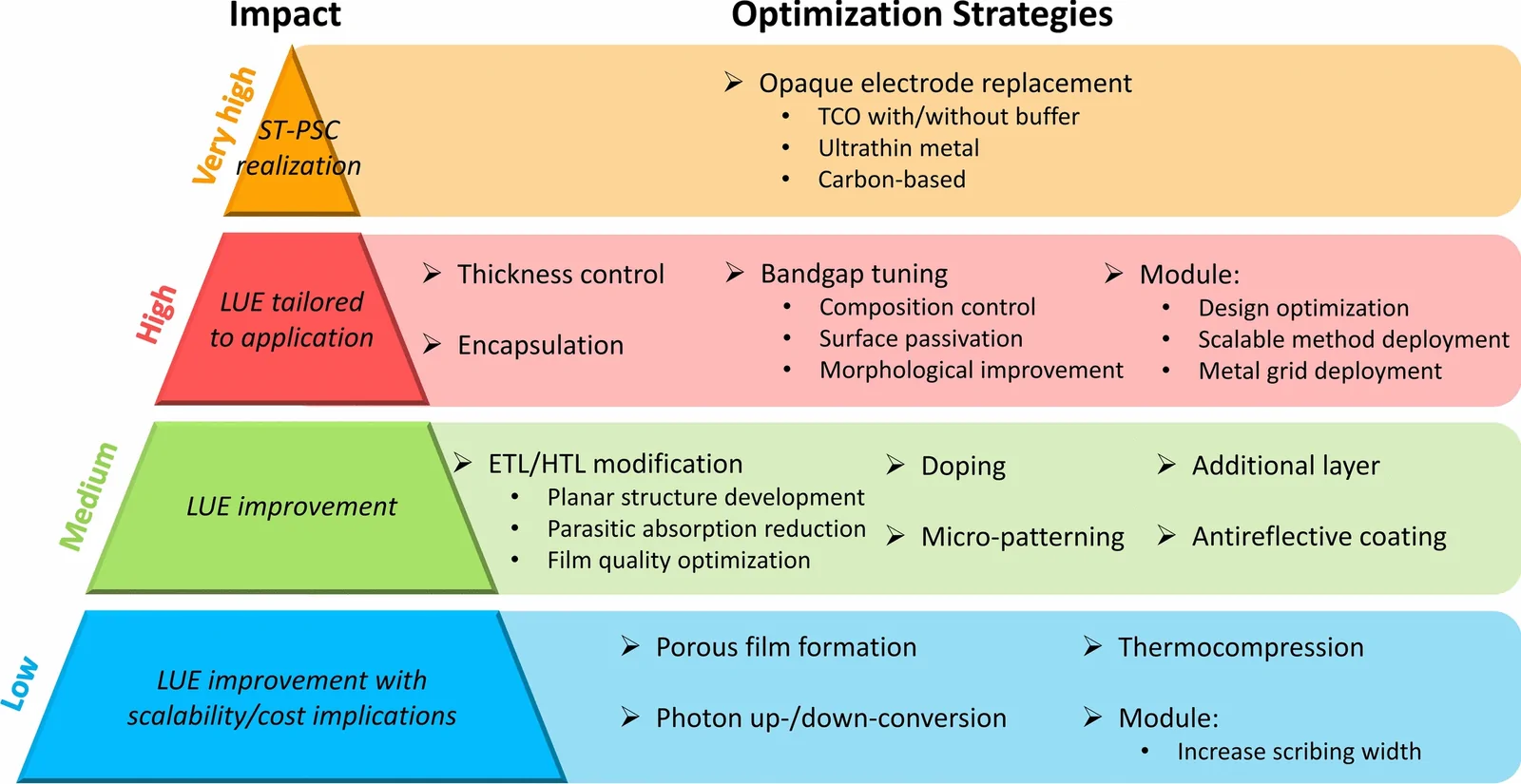
Ranking of optimization strategies according to their impact on ST-PSC performance

1. *Technical development*: Opaque PSCs have achieved a PCE of 27.0%, whereas ST-PSCs—even in nearly opaque bifacial configurations—reach only 23.3%, indicating substantial room for improvement, particularly in transparent rear electrodes. Further progress in this field requires a deeper understanding of the intricate interplay between charge transport, optical management, and fabrication compatibility within semitransparent architectures to simultaneously maximize the PCE, AVT, and LUE. Achieving this balance demands rational design strategies for the perovskite absorber, charge transport layers, and transparent electrodes, supported by low-damage, scalable deposition methods. At this stage, research should focus on maximizing LUE at the target AVT while simplifying fabrication complexity to ensure manufacturability. Figure 23 presents a guideline for optimizing ST-PSCs, highlighting key strategy and ranking them by their impact on LUE to distinguish the most critical approaches for achieving high-efficiency devices for the intended application.
2. *Module development*: To date, research on ST-PSC modules remains limited, and further work is needed to achieve high LUE. Future efforts should focus on overcoming key engineering challenges to enable reliable large-area deployment. Advances in laser patterning remain critical to minimize dead areas that do not contribute to efficiency or transparency, while series resistance can be further reduced through optimized transparent electrodes, metal grids, and subcell interconnections. Achieving uniform perovskite thickness and reproducible performance across large areas requires precise control of scalable deposition methods. Encapsulation strategies play a central role in ensuring long-term outdoor operation. In particular, module edges, which is vulnerable to moisture ingress, require robust sealing approaches such as laser edge cleaning combined with durable perimeter sealants or encapsulant films. For BIPV, VIPV, and wearable systems, sealing designs should ensure seamless integration with the host structures.
3. *Standardized reporting*: The figures of merit for ST-PSCs, including AVT and CRI, should be evaluated on complete devices rather than those without top electrodes to ensure standardized and comparable performance reporting. The calculation of AVT often lacks consistency, as the wavelength range used varies across different studies, leading to non-standardized LUE values. At the module level, effective AVT depends not only on the active perovskite area but also on opaque components (metal grids, busbars, interconnect lines), different layer stacks in scribed regions (P1–P3), and system-level elements (glass cover, frames, and junction boxes). Additionally, the influence of the encapsulation components, particularly their optical properties, on ST-PSC performance remains insufficiently explored. Therefore, it is recommended to use Eq. (2) with appropriate weighting based on the fractional area of each region to ensure consistency in reporting the transparency of ST-PSCs. Since high transparency of these components is critical for achieving optimal efficiency, their properties deserve greater attention to support the successful commercialization of ST-PSCs. Standardized AVT measurement and reporting are essential for systematic comparison of ST-PSC performance and for achieving compliance with regional regulations.
4. *Scaling up for industry*: Both solution- and vapor-based methods are compatible with scaling up each layer in ST-PSCs, although initial capex can strongly influence manufacturing cost in the short term. Accordingly, solution processing is better suited for start-ups and small firms due to its lower capex, whereas vapor-based approaches are more appropriate for established industrial players that can leverage existing equipment, trained personnel, and process know-how from prior PV technologies to reduce overall costs. In the long term, ST-PSC development should commit to a single, initially adopted approach (either solution or vapor based) to enable continuous fabrication and improved cost efficiency.
5. *Cost reduction*: ST-PSCs generally incur higher manufacturing costs than opaque PSCs due to buffer layers and indium-based electrodes. Buffer layer-free designs are therefore highly desirable, as they reduce material consumption, eliminate expensive deposition equipment, and shorten processing times. Previous demonstrations achieving ~ 20% PCE via soft sputtering provide strong evidence of the technical feasibility of this approach. Meanwhile, the high cost of ITO is expected to persist, highlighting the need for indium-free alternatives such as AZO, carbon-based films, or metal nanowires. Developing transparent electrodes via low-cost, scalable solution processing also offers significant potential for cost reduction. Although still in development stage, further advances in buffer-free and indium-free electrode design are critical to enable cost-effective ST-PSCs.
6. *Commercialization considerations*: Beyond material optimization, ST-PSCs must comply with established standards and certification protocols. Cells and modules should be properly encapsulated to withstand intensive stability testing (ISOS stage 3) and subsequently undergo standardized IEC tests (61,215, 61,646, 61,730) for performance, fire resistance, insulation, and electrical safety. For BIPV applications, particular attention should be paid to device stability under UV exposure, thermal cycling, damp-heat conditions, and mechanical stresses from wind, snow, and hail to ensure reliable multi-year operation. At this stage, standardized testing and certification deserve greater attention in research and development of ST-PSCs to ensure the devices meet the durability requirements for long-term outdoor deployment.
7. *Exploration of wider application*: ST-PSCs offer opportunities for applications that are challenging for conventional PV technologies. Continued development of 2 T and 4 T tandem architectures is recommended, while further exploration of 3 T configurations is warranted. Furthermore, the tandem device itself can be made semitransparent, which could benefit area-limited systems that require high-voltage operation with desired transparency. Bifacial ST-PSCs also require rigorous optimization, as they can maximize light utilization during day and night and potentially compete with tandem technologies in cost-per-power output. To expand market reach, ST-PSCs should be tailored for indoor and flexible designs, making the most of their ability to meet different system needs. Beyond these areas, niche high-technology applications, including space and underwater environments, offer additional opportunities for ST-PSC innovation. Closer collaboration among PV researchers, architects, IoT engineers, industry stakeholders, and policy makers will be key to translating ST-PSC technology into practical applications across multiple sectors.

With growing global interest, the next critical mission lies in resolving the remaining challenges to accelerate commercial implementation. We firmly believe that with systematic material and process optimization, ST-PSCs can evolve from laboratory prototypes into transparent power-generating devices that redefine the role of PV in a new era of sustainable energy.
